# Genetic Dynamic Analysis of the Influenza A H5N1 NS1 Gene in China

**DOI:** 10.1371/journal.pone.0101384

**Published:** 2014-07-08

**Authors:** Kaifa Wei, Yanhui Chen, Yina Lin, Yutian Pan

**Affiliations:** 1 School of Biological Sciences and Biotechnology, Minnan Normal University, Zhangzhou, China; 2 School of Life Sciences, Tsinghua University, Beijing, China; 3 The Engineering Technological Center of Mushroom Industry, Minnan Normal University, Zhangzhou, China; University of Texas Medical Branch, United States of America

## Abstract

The direct precursors of the A/Goose/Guangdong/1/1996 (GS/GD) virus lineage and its reassortants have been established geographically and ecologically. To investigate the variation and evolutionary dynamics of H5N1 viruses, whole-genome viral sequences (n = 164) were retrieved from the NCBI Influenza Virus Resource. Here, we present phylogenetic evidence for intrasubtype reassortments among H5N1 viruses isolated from China during 1996–2012. On the basis of phylogenetic analysis, we identified four major groups and further classified the reassortant viruses into three subgroups. Putative mosaic structures were mostly found in the viral ribonucleoprotein (vRNP) complexes and 91.0% (10/11) mosaics were obtained from terrestrial birds. Sequence variability and selection pressure analyses revealed that both surface glycoproteins (HA and NA) and nonstructural protein 1 (NS1) have higher dN/dS ratio and variability than other internal proteins. Furthermore, we detected 47 positively selected sites in genomic segments with the exception of PB2 and M1 genes. Hemagglutinin (HA) and neuraminidase (NA) are considered highly variable due to host immune pressure, however, it is not known what drives NS1 variability. Therefore, we performed a thorough analysis of the genetic variation and selective pressure of NS1 protein (462 available NS1 sequences). We found that most of positively selected sites and variable amino acids were located in the C-terminal effector domain (ED) of NS1. In addition, we focused on the NS1–RNA and NS1–protein interactions that were involved in viral replication mechanisms and host immune response. Transcriptomic analysis of H5N1-infected monkey lungs showed that certain PI3K-related genes were up-regulated.

## Introduction

The highly pathogenic influenza A virus subtype H5N1 (HPAI H5N1) was first isolated from a farmed goose in Guangdong province of China in 1996 [1]. HPAI H5N1 caused widespread poultry outbreaks and led to 18 cases of human infection in 1997 in Hong Kong, six of which were fatal [2]. Although the first wave of H5N1 infection was controlled by massive slaughter of poultry and compulsory mass vaccination, the virus was later found to circulate continuously in ducks in Southern China and underwent frequent and extensive reassortment, leading to the occurrence of a number of different genotypes [3]. In early 2004, the H5N1 virus has caused outbreaks in ducks, geese and chickens in 16 provinces of China, resulting in the establishment of multiple distinguishable sublineages [4]. Subsequently, more outbreaks have been reported in migratory waterfowls at Qinghai Lake in May 2005, and the virus continued to disseminate from Asia to Europe, the Middle East and Africa [5]. As of August 2013, 637 laboratory-confirmed human cases of H5N1 virus infection, including 378 fatalities, have been reported to the World Health Organization (WHO) from 15 countries (http://www.who.int/en/). Although sustained human-to-human transmission has not yet been reported, two recent studies have described the production of ferret-transmissible H5N1 avian influenza viruses [6], [7]. Additionally, the enzootic nature of H5N1 virus and the adaptive substitution in the virus could spark a new global pandemic [8].

There are five basic mechanisms determining changes in the genetic makeup and evolution of biological populations, including mutation, recombination, natural selection, genetic drift and migration [9]. Of the diverse array of RNA viruses, HPAI H5N1 displays noticeable features such as high genetic variability and rapid evolution. These significant traits can be ascribed to the rapid replication and high evolutionary rate of HPAI H5N1 (in the range of 1×10^−3^ to 8×10^−3^ nucleotide substitution per site per year [10]). Reassortments and point mutations are two important ways to generate novel influenza virus strains and contribute to viral evolution and virulence change [11]. Influenza surveillance in Southern China showed that the A/goose/Guangdong/1/96 (GS/GD) virus lineage has generated a plethora of genotypes since 2000 [12], [13]. As reported previously, homologous recombination plays an important role in the evolution of DNA and RNA viruses [14]. For negative sense single-stranded RNA (ssRNA) viruses (e.g., HPAI H5N1), multiple copies of the nucleoprotein (NP) molecules, a ssRNA genome segment and the polymerase complex (PB2, PB1 and PA) are packaged into each viral ribonucleoprotein (vRNP) particles. Therefore, template switching during viral replication, which has played an important role in the virulence or fitness of influenza A viruses (IAVs), is prevented [15]. Although there has been some debate about whether homologous recombination occurs in HPAI H5N1, Lam *et al*. reported that the majority of homologous recombinants were detected in H5N1 and H9N2 subtypes and the geographic distribution of the mosaic sequences was uneven, with over half of isolates sampled from China [16].

The IAVs are enveloped virus with an single-stranded negative-sense RNA genome belonging to the family *Orthomyxoviridae*. IAVs subtype H5N1, also known as A (H5N1), can cause illness in humans and many other animal species [4]. Among the molecular determinants of virulence in mammalian hosts are the polybasic cleavage site in HA, the polymorphism in vRNP complex, and the proapoptotic protein (PB1-F2) [17]. Point mutations associated with antiviral drug resistance, such as the S31N mutation in M2 and mutations at positions 119, 275, 293 and 295 of NA protein, have been observed by previous studies [18]. In addition, several amino acid changes in PA (T515 A), PB2 (E627K or D701N) and the nonstructural (NS1) protein (V149A) have been reported to determine viral virulence and regulate viral replication in their corresponding hosts. To restrict virus proliferation, virus-infected cells usually develop an effective antiviral immune response. However, IAVs have evolved multiple mechanisms to avoid these responses [19]. The viral NS1 protein, which contains an N-terminal double-strand RNA-binding domain (RBD) and a C-terminal effector domain (ED), is an antagonist of antiviral type-I interferon (IFN) response in the host. Moreover, NS1 reduces the antiviral effects of IFN-induced proteins, such as dsRNA-dependent protein kinase R (PKR), 2′5′-oligoadenylate synthetase (OAS)/RNase L and retinoic acid-inducible gene 1 (RIG-I) [20]. The NS1 protein also modulates viral infection and host cell signaling pathways by interacting with the host molecules [21], [22].

Given the critical role of PI3K/Akt signaling, it is not surprising that H5N1 viruses have evolved multiple strategies to activate PI3K/Akt signaling as a means to increase their replication efficiency [23]. Phosphatidylinositol 3-kinases (PI3Ks) are a family of cellular, heterodimeric enzymes that consist of a regulatory subunit (p85) and a catalytic subunit (p110). PI3K is activated by binding of the src-homology (SH) domain in the p85 subunit to autophosphorylated tyrosine kinase receptors [24]. The p110 subunit of PI3K phosphorylates the lipid substrate phosphatidylinositol-4,5-bisphosphate (PIP2) to produce phosphatidylinositol-3,4,5-trisphosphate (PIP3), leading to the specific membrane-recruitment of a diverse range of signaling proteins [25], [26]. In addition, both PI3K and its downstream effector (Akt) are important regulators of cell growth, proliferation and survival [27]. Recent studies suggested that the NS1 protein can interact with the PI3K either by binding to Crk/CrkL SH3 domains [28] or direct binding and activation of Akt [29]. Moreover, the ED of NS1 binds specifically to the inter-SH2 (iSH2) domain of p85β subunit, thereby leading to steric changes within p85β to release the inhibitory effect on p110 [30].

Each viral gene plays a significant role within the virus life cycle. Therefore, understanding the evolution and dynamics of each gene can provide new insights into the molecular mechanisms determining the genetic structure and evolution of HPAI H5N1 in China. Here, we examined the reassortment, recombination, sequence polymorphism and selection pressure of HPAI H5N1 in China from 1996–2012. Sequence-based analysis suggested that variation is more common in surface glycoproteins and NS1 protein, indicative of their vital role in viral life cycle. HA and NA are considered highly variable due to host immune pressure, however, it is not known what drives NS1 variability. Therefore, we performed a thorough analysis of the genetic variation and selective pressure of NS1 protein (462 available NS1 sequences). Activation of the host-cell PI3K pathway has recently been described as an additional direct method by which NS1 may limit induction of apoptosis, therefore, we investigated the downstream effects of the activation of PI3K pathway by measuring expression of 85 cellular genes in macaque lung tissues in response to the infection with an influenza strain A/Anhui/2/2005 (H5N1).

## Materials and Methods

### Sequence Data Collection and Alignment

Nucleotide and protein sequences of all genomic segments of 164 H5N1 influenza viruses isolated from avian and human hosts (sampled during 1996–2012) were downloaded from the NCBI Influenza Virus Resource in April 2013 (http://www.ncbi.nlm.nih.gov/genomes/FLU/FLU.html). Only full-length gene sequences were analyzed. Sequences from the same viral strain were removed such that one copy of the duplicate sequence was retained. The coding sequences of each genome segment were aligned using MUSCLE v3.6 [31] and manual editing of alignments were performed in MEGA 5 [32]. The alignments of eight gene segments (PB2 = 2277 nt; PB1 = 2271 nt; PA = 2148 nt; HA = 1656 nt; NP = 1494 nt; NA = 1407 nt; MP = 979 nt; NS = 835 nt) as well as four coding regions (M1, M2, NS1, and NS2) were used for analysis.

### Phylogenetic Analyses

Phylogenetic trees were reconstructed from the 12 alignment datasets using the maximum likelihood (ML) approach implemented in PhyML 3.0 [33]. In order to ensure the reliability of different phylogenetic groupings, we compared the ML topology with the topologies sampled in the Bayesian Monte Carlo Markov chain (BMCMC) analysis performed in MrBayse 3.2.1 [34], and with bootstrapping analyses of 1,000 pseudo-replicate datasets [32]. Early appearing and phylogenetically unresolved lineages were mostly composed of viruses isolated from earlier outbreaks (during 1996–2004). Here, we excluded poorly supported branches (e.g., earlier viruses), therefore, only four major groups were identified. Best-fit models of nucleotide substitution were selected by using jModeltest 0.1.1 based on Akaike Information Criterion (AIC) [35]. The following preferred models were used: GTR+I+G for PB2, PA, NP and M2, TVM+I+G for HA, MP, M1, TIM1+I+G for PB1, TrN+G for NA, TVM+G for NS, GIR+G for NS1 and TPM1uf+I+G for NS2. Phylogenetic trees were visualized with Figtree 1.3.1 [36]. In most cases, phylogenies were rooted to A/equine/Prague/1/1956 (H7N7), whereas the HA, NA and PB1 gene trees were rooted to duck/Hokkaido/51/96 (H1N1), A/chicken/Scotland/1959 (H5N1), and A/pintail duck/Alberta/628/79 (H6N8), respectively.

### Detecting Mosaic Sequences

We screened homologous recombination in each gene segment of HPAI H5N1 using various exploratory methods implemented in Recombination Detection Program (RDP) version 4.22 [37], including RDP, GENECONV and MAXCHI. Sequences with mosaic recombination signals were identified as those with Bonferroni-corrected p-values <0.05 in more than one detection method. Putative mosaic structures (four previously unreported mosaic sequences) were investigated using four small subsets of genome sequences (represented by consensus sequences) of H5N1 virus. Here, each of subsets included sequences of early viruses (n = 2), group 1 (n = 6), group 2 (n = 4), group 3 (n = 6), group 4 (n = 6) and putative recombinant viruses (n = 1). For each sample, the eight gene segment alignments were manually concatenated in the order of their length to generate a single alignment of full genome sequences, and the resulting alignment was analyzed using the bootscanning method implemented in the SimPlot v3.5.1 [38]. Finally, confirmed mosaic sequences were excluded from subsequent evolutionary analyses.

### Genetic Distance and Sequence Polymorphism Analyses

The 164 full-length HA sequences were used to estimate intergroup distances in MEGA 5.1 by the Jukes and Cantor method with 1,000 bootstraps [32]. Sequence polymorphism of all gene segments and subsequent tests were performed in DnaSP 5.0 software [39]. The number of haplotypes (Hp), nucleotide diversity (π) and average number of pairwise nucleotide differences within the population (K) were all calculated according to Nei [32]. Watterson's mutation parameter (θ) was calculated from the number of polymorphic sites (S) [40]. Eta (η) represents the total number of mutations. The rates of non-synonymous substitutions (Ka) and synonymous substitutions (Ks) were calculated according to Nei and Gojobori [41]. Neutrality tests including Tajima's test and Fu and Li's D and F tests were also conducted using the DnaSP [39].

### Detection of Selection Pressure

The maximum likelihood estimation (MLE) under the MG94 substitution codon model was used to detect the overall selection pressure of each gene segment. The selection pressure was investigated by estimating the ratio of non-synonymous to synonymous nucleotide substitutions (ω =  dN/dS) with the two-rate fixed effect likelihood (FEL) method available in the Hyphy 2.1.0 [42]. Positively selected sites were identified using single likelihood ancestor counting (SLAC), FEL and internal fixed effect likelihood (IFEL) methods with a significance level of 0.05 [42]. In all cases, dN/dS estimates were based on ML trees generated by PhyML and the best-fitting substitution models were also selected by Hyphy software.

### Amino Acid Variability Analysis

The amino acid variability of each segment was calculated according to the formula of Kabat [43]. First, the variability of each position was calculated as variability  =  N/F, where N represents the number of different amino acids at a given position, and F represents the frequency of the most common amino acid at that position. A completely conserved position has a variability of 1 (all sequences have same amino acid). Second, the variability was averaged across the positions to give an overall variability for the corresponding segment. In addition, the frequency of amino acids at each position was evaluated using the EMBOSS program PROPHECY [44]. The matrix obtained was converted into polymorphism frequency by setting a cut-off of 1% at each position.

### Homology Modeling for NS1

Homology models of NS1 protein were created using the SWISS-MODEL server [45] with the aim of producing homology models of four different NS1 isolates (AH/2/05, CK/GD/1/05, HK/156/97 and GS/GD/1/96). Crystal structures of the NS1-p85β and the NS1-p85β-p110 complexes as well as RBD and ED of NS1 are available from the Protein Data Bank (PDB) (http://www.rcsb.org/pdb/home/home.do). To visualize and edit the PDB models, interactive molecular graphics program Chimera v 1.8 was used [46], [47].

### Microarray-based Expression Analysis

For expression analysis, the microarray-derived gene expression data of PI3K/Akt signaling pathway components were downloaded from the GEO database with accession of GSE 37149 [48]. The data was normalized using a robust multi-chip average (RMA) algorithm. Log10-transformed expression values were loaded into R-2.15.2 and Bioconductor for expression analysis (http://www.bioconductor.org/). The limma package was applied to model the systematic parts of data by fitting a linear model in the function lmFit [49]. The heatmaps representing log10-transformed probe intensities were then generated with gplots package (http://www.bioconductor.org/).

## Results

### Phylogenetic Relationships among H5N1 Viruses in China

Phylogenetic trees were reconstructed from 12 separate gene datasets using the full genome of 164 HPAI H5N1 viruses obtained from GeneBank (Figure S1). Full details of the sequences used in this study are provided in Table S1. We performed a phylogenetic analysis of Chinese H5N1 viruses and identified four major groups for all gene segments (Figure S1) with the exception of M2 (poorly supported branches for group 1, 3 and 4). The phylogenetic trees obtained here were generally consistent with our previous study with slight differences in phylogenetic groupings [13]. Here, we chose to be conservative and excluded poorly-supported branches (e.g., earlier viruses) when grouping (see Materials and Methods for details). In order to correlate these groups with the novel international nomenclature system recently designed by the WHO/OIE/FAO H5N1 Evolution Working Group, we used all 164 HA gene sequences to estimate the intergroup distance. All groups exhibited values significantly above the minimal limit of 1.5% assessed by pairwise analysis (Table S2). Group 1 viruses, which were mostly isolated from chickens in Xinjiang and other northern provinces of China, can be further divided into two distinct subgroups (group 1A and group 1B).

**Figure 1:**
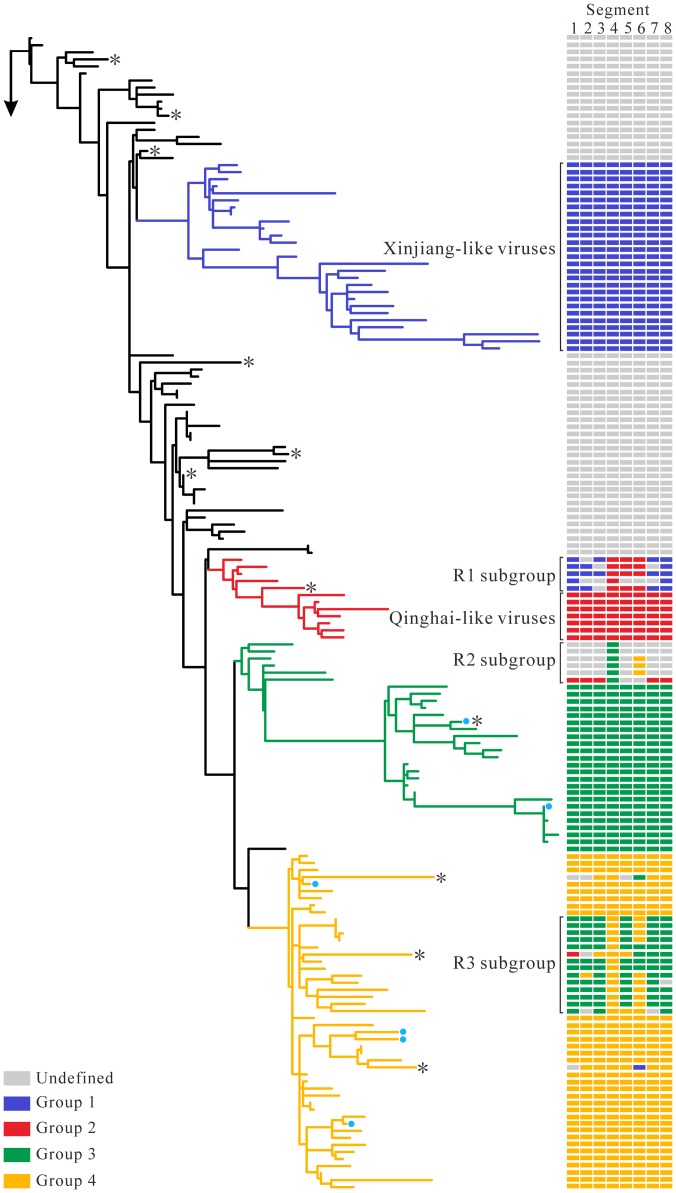
Maximum-likelihood phylogeny of the HA gene. The identical phylogeny with virus names is provided in Figure S1D. Coloured boxes adjacent to branch tips show the group classification of each gene segment of HPAI H5N1. Reassortant subgroups (R1, R2, R3) are indicated with square brackets. Six isolates sampled from southeast China are designated as blue circles. The asterisks denote the phylogenetic position of eleven recombinant viruses (CK/HuB/wj/97, CK/HB/108/02, CK/HB/718/01, DK/ZJ/bj/02, CK/GS/44/04, ML/GX/wt/04, CK/JX/25/04, DK/HN/8/08, DK/EC/108/08, CK/GZ/7/08, CK/SD/A-1/09).

Phylogenetic trees constructed by the ML, NJ and BMCMC methods (see Materials and Methods for details) revealed similar relationships, but genomic reassortment still resulted in isolates being positioned within different phylogenetic clades. Herein, three subgroups (R1, R2 and R3) were further identified based on branching inconsistencies observed from phylogenies. For HA, NP and NA genes, the R1 subgroup was most closely related to Qinghai-like viruses of group 2. However, the other phylogenetic pattern was observed in the remaining segments, which had a close relationship with the Xinjiang-like viruses of group 1 (Figure 1 and Figure S1). Furthermore, the placement of six isolates sampled from avian during 2003–2005 differed between HA and other gene segments (designated as R2 in Figure 1). Phylogenetic analysis of the HA gene showed that the R2 subgroup clustered inside group 3. However, unlike the HA gene, the remaining gene segments occupied either an intermediate position between group 1 and group 4 or clustered with early viruses (Figure 1, Figure S1 and Table S3). Phylogenetic analysis showed that R3 subgroup viruses belonged to group 3 for most gene segments, but no such evolutionary pattern was observed in HA and NA genes (Figure 1, Figure S1 and Table S3). Six isolates (shown in blue circles on branches) sampled from southeast China were most closely related to earlier viruses in all phylogenies with the exception of HA, which clustered with group 3 or group 4 viruses (Figure 1, Figure S1 and Table S3). In addition, the isolate DGWT/HN/79/05 (Figure S1) tended to cluster near the root in the MP, M1 and M2 phylogenies and showed a high degree of sequence similarity with the isolate DK/ZJ/2245/11, whereas this isolate belonged to group 1A in the remaining gene segments. These observations indicated another reassortment event and the complexity of the evolution of H5N1 viruses in China.

### Mosaic Genome Structure in HPAI H5N1

**Figure 2:**
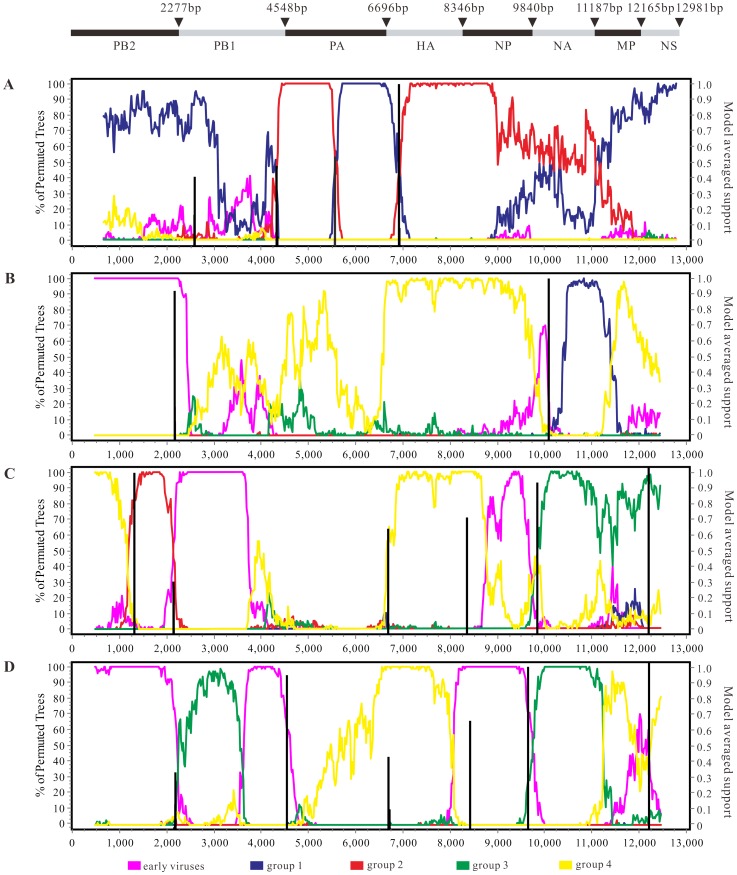
Bootscan analysis and GARD estimates of concatenated influenza virus genomes. CK/JX/25/04, CK/SD/A-1/09, CK/GZ/7/08 and DK/EC/108/08 were used as query sequences in (A), (B), (C) and (D), respectively. Schematic diagram of concatenated influenza virus genomes was showed at the top. Consensus sequences representing viral groups, window size of 1,000 bp and step size of 40 bp, were used for bootscan analysis.

A suite of methods implemented in RDP 4.22 identified 11 isolates that have a mosaic structure possibly resulting from recombination events, including one mosaic sequence detected in NA and 10 mosaic sequences in RNP segments. Interestingly, we found that 91.0% (10/11) mosaics identified here were isolated from terrestrial birds (Table 1), which was consistent with a previous report [50]. To investigate four previously unreported mosaic sequences, the selected datasets of manually concatenated full genomes of H5N1 viruses were analyzed (Figure 2). The CK/JX/25/04 strain fell within group 1 in three gene trees (PB2, MP and NS), but it was similar to group 2 viruses in HA, NA and NP phylogenies (Figure 2A, Figure S1 and Table S3). The mosaic structure of CK/SD/A-1/09 was evident in bootscanning analyses (Figure 2B, Figure S1 and Table S3). In most phylogenies, the CK/SD/A-1/09 strain formed a well-defined cluster with group 4, whereas this isolate was most closely related to earlier viruses and three domestic poultry viruses (CK/JS/18/08, CK/HB/A-8/09 and CK/hd/4/08) of group 1 in PB2 and NA genes, respectively. As shown in Figure 2C, the query sequence, CK/GZ/7/08, is closely related to group 2 viruses in part of the sequences of PB2. However, the CK/GZ/7/08 strain has a similar mosaic pattern with group 3 or group 4 viruses in other genomic regions apart from PB1. Phylogentic analysis of PB1 showed a long branch separating CK/GZ/7/08 from other H5N1 viruses (Figure S1). The concatenated aligned gene sequence of DK/EC/108/08 was characterized as a recombinant, which shared a high degree of sequence identity with that of group 4 in PA, HA, MP and NS genes and was more similar to the consensus sequence of group 3 or earlier viruses in other genomic regions (Figure 2D, Figure S1 and Table S3). Lam *et al*. has previously found that most of the mosaic sequences that belonged to subtype H5N1 were sampled from Mainland China [50]. One noticeable feature was that the majority of mosaic sequences identified here were sampled between 1997 and 2004. However, such events are not surprising given the increased sequencing efforts during this period as well as some experimental artifacts. Further analysis using a method called Genetic Algorithms for Recombination Detection (GARD) suggested that the breakpoints of four recombinant strains were detected in the NA gene and RNP subunits. The results showed that the mosaic breakpoints were located at nucleotide positions 2657, 4365, 5566 and 6915 of the sequence CK/JX/25/04, while only two breakpoints were found in sequence CK/SD/A-1/09 including positions 2175 and 10086. In addition, five (positions 1252, 2118, 8346, 9825 and 12174) and six (positions 2168, 4545, 6727, 8406, 9666 and 12440) well-supported breakpoints were detected in the query sequences CK/GZ/7/08 and DK/EC/108/08, respectively.

**Table 1:** Summary of the mosaic sequence identified in this study.

Strain name	Algorithm & Recombination P-Value			Segment
	RDP	GENECONV	MAXCHI	
CK/HuB/wl/97	1.04624E–10	1.63304E–08	5.08626E–07	PA
CK/HB/718/01	1.74863E–06	0.0004589	9.09778E–11	PB1
CK/HB/108/02	5.9039E–13	7.45188E–13	0.005966	PA
DK/ZJ/bj/02	2.388E–08	0.01401	3.055E–07	PA
CK/GS/44/04	5.02199E–13	2.20794E–09	1.72289E–12	PB2
ML/GX/wt/04	4.59292E–10	NS	9.73989E–07	PB2
CK/JX/25/04	6.215E–09	1.49817E–12	0.000001037	PA
CK/GZ/7/08	1.124E–07	3.195E–14	3.01415E–14	PB1
DK/EC/108/08	3.76E–23	1.83697E–21	6.14628E–13	PB1
DK/HN/8/08	4.14204E–08	1.58246E–06	0.009562379	NP
CK/SD/A-1/09	NS	0.011533897	0.005948611	NA

NS: No significant P-value was recorded for this recombination event.

### Polymorphism and Selective Pressure

In order to assess the polymorphism of eight genome segment alignment datasets, as well as four coding sequences (M1, M2, NS1, and NS2), we performed a series of statistical tests to obtain different features of molecular polymorphism in H5N1 viruses (Table S4). Previous studies suggested that the levels of DNA polymorphism observed for a specific gene region were strongly correlated with regional rates of recombination [51]. The polymorphism analysis revealed that the neutrality tests for the polymerase complex (PB2, PB1 and PA) were significant but associated with lower Ka/Ks ratios and higher diversity when compared to other genes (Table S4), suggesting a population in expansion rather than positive selection. Dugan V.G., *et al*. has reported that the fitness landscape for RNP subunits is determined by functional viability rather than by cross immunity, with less selective pressure to fix advantageous mutations [52]. In contrast to less selective pressure seen in the RNP subunits, the Tajima's test was significant with a high Ka/Ks ratio in HA, NA and NS gene segments. The significant feature was that the average Ka/Ks ratios were below 1.0 (Table 2 and Table S4) for all gene segments, most likely suggesting that they were subject to purifying selection [53].

**Table 2:** Estimation of selection pressure and sequence variability for H5N1 influenza virus.

Gene	Length (nt)	Positively selected site^a^			Sites under positive selection	*d_N_/d_S_* (95% CI)^b^	Variability (%)^c^
		SLAC	FEL	IFEL			
PB2	2277	None	None	None	0	0.102 (0.094–0.110)	1.559
PB1	2271	215, 384, 610	215, 384, 610	64, 215, 573, 577, 610	6	0.103 (0.094–0.111)	1.593
PA	2148	337, 544	101, 201, 237, 337, 544, 669, 712	237, 337, 544	7	0.162 (0.151–0.173)	1.810
HA	1656	138, 140, 156	115, 138, 140, 141, 156	45, 115, 138, 140, 141, 156	6	0.287 (0.266–0.308)	1.914
NP	1494	None	452, 482	452	2	0.089 (0.080–0.100)	1.470
NA	1407	2, 340	2, 46, 70, 73, 74, 84, 100, 427	8, 46, 74, 76, 100, 340	11	0.246 (0.226–0.268)	1.934
M1	756	None	None	None	0	0.129 (0.110–0.150)	1.385
M2	291	18, 82	18, 82	14, 18, 82	3	0.636 (0.531–0.753)	1.823
NS1	690	197	127, 197, 205	127, 171, 185, 197, 209, 212, 227	8	0.434 (0.395–0.477)	2.428
NS2	363	None	14, 34	14, 52, 111	4	0.398 (0.340–0.462)	2.098

a FEL, iFEL, SLAC significance levels are indicated by *P* values and site under positive selection (*P*<0.05) are detected by at least one method.

b The dN/dS ratios are estimated using the FEL method available in the Hyphy package.

c The variability of each segment is calculated at the amino acid level.

Further site-specific selection analysis helped to identify 47 positively selected sites that were detected by at least one of three methods (SLAC, FEL, and IFEL) (Table 2). Among 11 positively selected sites identified in the NA gene, sites 46 and 340 are located in the T-cell and B-cell antigenic regions, respectively [54]. Furthermore, three sites (sites 46, 74 and 340) previously identified as undergoing changes in selective pressure during host shifts from birds to humans were also detected here [55]. In HA, four residues located in or close to antigenic sites A and B (sites 115, 138, 140, 141) and site 156 were estimated to be a potential N-linkedglycosylation (NLG) site (Table 2). Site 45 was previously identified as a positively selected site in certain areas, such as China [56], suggesting that some sites under positive selection in H5N1 vary from one region to another. Here, we found that two sites (sites 14 and 18) under positive selection in M2 are located in the extracellular domain and one site (site 82) in the cytoplasmic domain (Table 2). However, the M1 protein, which plays an important role in virus assembly, is under strong negative selection pressure (mean dN/dS  = 0.129) and the positively selected site was not identified in M1 as expected. For the NS gene, 12 positively selected sites were detected in its two coding regions (NS1 and NS2) and mostly distributed in the ED of the NS1. In addition, evidence of positively selected sites in RNP segments was also discovered except for PB2, but the biological function of the residues is not well-understood (Table 2).

### Variability and Conservation in the NS1 Protein of the H5N1 Virus

**Figure 3:**
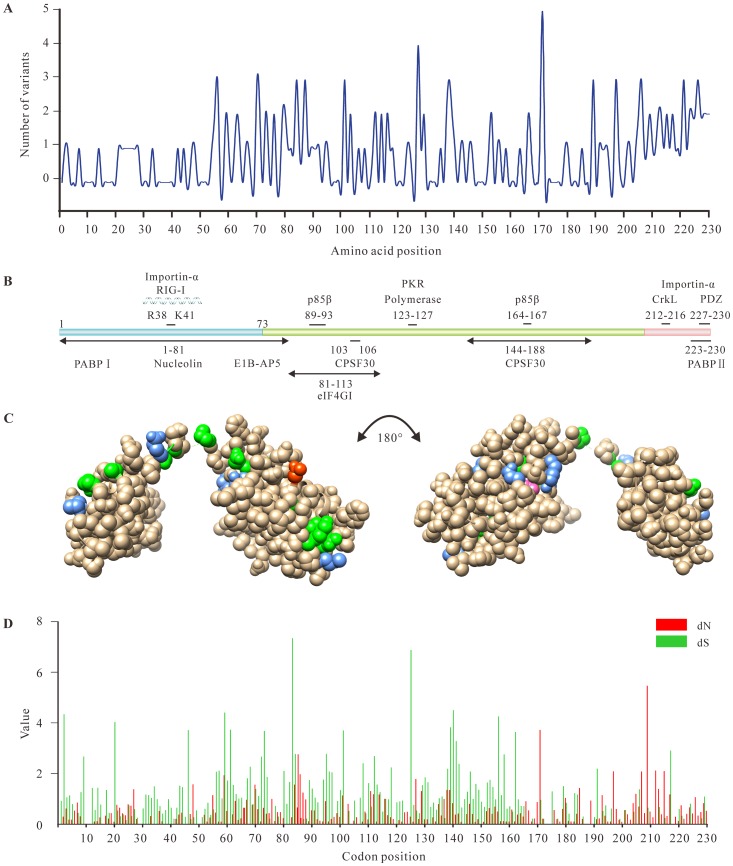
Sequence variation along the non-structure 1 protein (NS1) sequence. (A) Number of polymorphisms (variants occurring in more than 1% sequences examined) at each position. (B) Schematic representation of the NS1 protein of H5N1, together with its known interactors. (C) Variation within RNA binding domain (RBD) and effector domain (ED) of NS1. Position containing 2 polymorphisms are coloured green, 3 polymorphisms are coloured cornflower blue and 4 or above are coloured hot pink and red, respectively. Residue positions have been imposed upon the 3D structure of NS1 from the Protein Data Bank (3F5T). (D) Panel shows the distribution of non-synonymous (dN) and synonymous (dS) substitution (the number of substitutions per site) along the NS sequence.

Sequence variability showed that HA, NA and NS1 contribute the most to the variability of virus genomes (Table 2). It is well-known that high levels of variability of surface glycoproteins are due to the host immune selective pressure [57]. However, the evolutionary forces responsible for the sequence variation of the NS1 are unclear. NS1 protein is recognized as one of major determinants of viral virulence and pathogenicity [4]. Considering the contribution of NS1 to the genetic variability of H5N1 virus, we then focused on viral protein NS1 (Table S5), in which we identified 10 sites that are under selective pressure (Figure 3). As shown in Figure 3A, nearly one half of the amino acids (109/230) within the NS1 sequence were completely invariable while other variable amino acids were mostly focused to the C-terminal portion of NS1 protein (positions 74–230).

The functional RBD of NS1 consisting of 73 amino acids was relatively conserved. Four amino acids (Arg35, Arg37, Arg38 and Lys41) located at the nuclear localization signal (NLS) region of NS1 (Figure 3B and Table S6) were invariable due to their ability to bind to dsRNA. Most of the isolates possessed serine at position 42 in NS1 protein, a residue known to be associated with viral virulence [58]. Compared with the RBD, the ED exhibited a high level of variability in certain regions, suggesting that the immune responses by the host exert strong selective pressure on the ED (Figure 3C). A site-by-site analysis of variability within each of these regions provided additional evidence of selective pressure on the ED. Sixty percent of amino acids at positions 81–113 that can effectively interact with the eukaryotic initiation factor (eIF4GI) were variable [59], while no variability was found around residues 103 and 106. In addition, a striking loop (position 137 to 142), which may bind with the p85β regulatory subunit of PI3K [60], was variable except the amino acid at position 142 (Figure 3). Interestingly, previous reports suggested that NS1-138F was highly conserved in all IAVs [61], whereas position 138 can be present as cysteine, phenylalanine, tyrosine or serine residues in this study (Figure 3 and Table S6). The short C-terminal peptide motifs of 4–5 amino acids showed remarkable variability (Figure 3 and Table S6).

Considering that the variability plot only reflected non-synonymous nucleotide substitutions, we further calculated the ratio of non-synonymous/synonymous nucleotide substitution rates for 462 NS1 sequences. As shown in Figure 3D, dS was significantly higher than dN in the RBD (see Materials and Methods), but the results were reversed in the two regions of the ED, including residues 86–89 and residues 170–230. Although the NS1 protein exhibited evidence of purifying selection acting on the coding sequence (ω = 0.463), we also found 10 sites (codons 48, 86, 185, 197, 205, 207, 209, 212, 215 and 226) under positive selection in the NS1 gene by FEL and SLAC methods. As expected, of 10 positively selected sites detected here (Figure 3D), most of them were identified within the above mentioned regions of the ED and only one position (codon 48) was detected in the RBD, reflecting that selective pressure on ED was stronger than that on RBD.

### NS1 Structure Analysis and Host Innate Immune Response

The phylogenetic relationships of the NS genes have revealed two major gene lineages, referred to as alleles A and B. The NS1 gene of GS/GD/1/96 and several viruses isolated from duck and goose belonged to the B allele, while the remaining NS1 genes, including those of the 1997 human Hong Kong viruses, belonged to the A allele (Figure S2A and Figure S3). Of 462 NS1 gene sequences, 448 and 14 sequences belonged to allele A and allele B, respectively. Allele A and allele B NS1 proteins showed at least 96.0% and 77.9% amino acid identity, respectively, but the similarity between the alleles was only 63.4%.

Structurally, the NS1 protein of H5N1 virus has two well-characterized functional domains: RBD and ED. Sequence analysis revealed that the Arg38 and Lys41 were highly conserved in 462 available NS1 sequences (Figure S2B-C and Table S6), which were required for the RNA-binding activity of NS1 [62]. The pocket of the ED in the NS1 protein interacts with a number of host proteins. For example, the NS1-CPSF30 complex was confirmed to prevent CPSF30 from binding cellular pre-mRNAs [63]. Two amino acid residues (F103 and M106) are highly conserved in most of the NS1 proteins and crucial for stabilizing the NS1-CPSF30 complex (Table S6 and Figure S2D). Nonetheless, the F103L and M106I mutations were still detected in highly virulent human H5N1 isolates sampled from Hong Kong in 1997 (Figure S2B). Interestingly, although their NS1 proteins contain L (not F) at position 103 and I (not M) at position 106, they can interact with viral polymerase complex and the NP protein to stabilize the NS1-CPSF30 complex [64]. In addition, the allele A of NS1 protein contains Y instead of F at position 103 and this mutation at position 106 only occurs in the isolate DK/GD/07/2000 (Figure S2B). With respect to the role of the NS1 protein in virulence, we examined the distribution and frequency of the four C-terminal amino acids of 462 NS1 sequences and identified a PDZ domain ligand (PL) at the C terminus. The conserved sequence ESEV was found in most H5N1 viruses (70.1%), especially in avian and human isolates, but six types of PL motifs were not seen in mammalian isolates (Table S7). In addition, the viruses with the PL motif EPEV (n = 22) and mutation at position 92 (D92E) were mainly isolated from the 1997–1998 outbreaks in Hong Kong. Herein, a deletion of amino acids 80–84 was found in allele A NS1 protein sequences except a small branch of the A allele (highlighted in green within Figure S3) which contained five amino acid residues “AIASS” at the position 80–84 of the NS1 protein. However, allele B NS1 protein comprised the sequence TIASV or TIASL at the same region (Table S7).

Apart from the functions mentioned above, NS1 protein is capable of influencing the apoptotic process in the host cell by interacting with the p85β regulatory subunit of PI3K, thereby activating PI3K/Akt signaling [64], [65]. The p85β subunit contains one N-terminal SH3, one B-cell receptor homology (BH) and two SH2 domains [66]. Molecular modeling suggested that the NS1 SH3 binding motif 1 (SH3-bm-1) and residues 137–142 may interact with different NS1 binding domains or sites of p85β (Figure S2E–F). Moreover, p85β also interacts with the p110 catalytic subunit and results in the up-regulation of PI3K activity [30]. However, one of the H5N1 viruses (A/Chicken/Guangdong/1/2005) characterized by a single amino acid change (F to Y) at position 138 failed to activate the PI3K/Akt signaling pathway [67]. Additionally, although no direct interaction was detected between NS1 protein and p110, NS1 protein was close to three residues (Glu-542, Glu-545 and His-1047) in helical and kinase domain of p110 (Figure S2G).

### Expression Profile of PI3K/Akt Signaling Components Mediated by NS1 Protein

**Figure 4:**
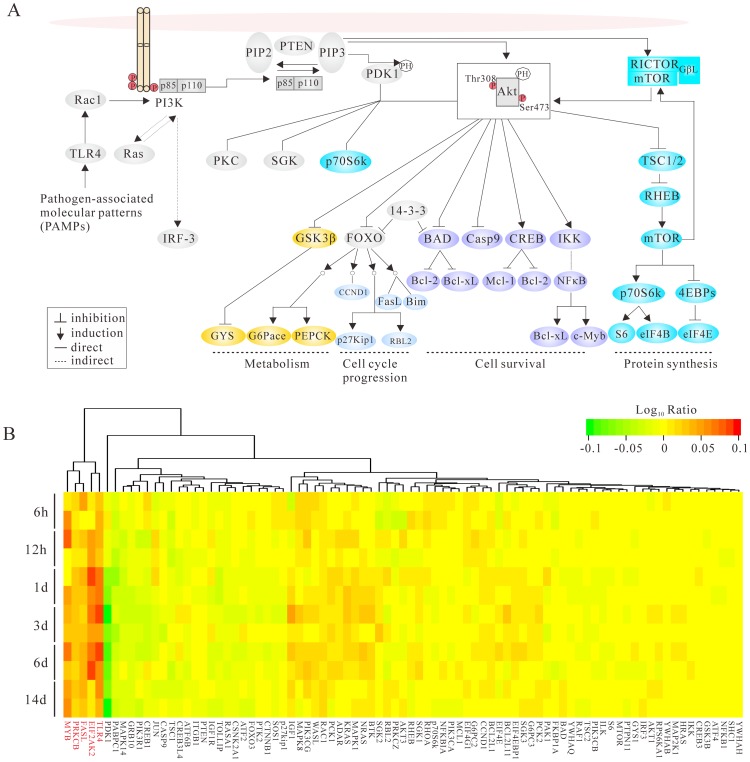
PI3K/Akt signaling and differential gene expression analysis. (A) Schematic diagram for the regulation of PI3K-Akt signaling pathway. (B) Overview of temporal differential gene expression in rhesus macaques infected with A/Anhui/2/2005 (H5N1) at different time points. A color scale indicating expression levels for the heat map is shown at the top right. Genes exhibited up-regulated expression pattern over time are highlighted in red.

The multifunctional NS1 protein is an important virulence factor of HPAI H5N1 and contributes significantly to disease pathogenesis by modulating a number of host-cell processes [68]. Members of the PI3K family control several cellular responses including cell growth, metabolism, proliferation and survival [69]. In addition, previous studies suggested that the PI3K was identified to be activated upon IAVs infection. Although a weak and transient induction of PI3K is caused by viral entry, a greater and more sustained activation of PI3K is activated by the viral NS1 protein to prevent premature apoptosis [70]. To understand the temporal and spatial transcription patterns of the relative genes of PI3K/Akt signaling pathway, hierarchical clustering was performed to visualize gene expression patterns. The microarray-derived gene expression data revealed that infected macaques were monitored for 14 days (6 h, 12 h, 1 d, 3 d, 6 d and 14 d). Datasets from six experiments infected with 10^7^ EID_50_ of A/Anhui/2/2005 (H5N1) in 4 mL of phosphate-buffered saline (PBS) and one mock-infected control inoculated with 4 mL of PBS have been analyzed [48]. The log10 (treated/control) ratio values are illustrated by a heat map (Figure 4), showing the fold change of each gene compared with the control. In this study, we investigated the role of NS1 protein in antiviral and apoptotic responses, especially in the PI3K/Akt signaling pathway and also examined the expression level of genes in P13K/Akt pathway at macaque lung tissues upon infection of an influenza strain A/Anhui/2/2005 (H5N1).

As illustrated in Figure 4, most of the genes show similar expression patterns for samples collected from the same time points, albeit some distinct differences (e.g., MYB gene). Among 85 key players involved in PI3K signaling (Figure 4 and Table S8), five genes exhibited up-regulated expression pattern over time (highlighted in red within Figure 4B), especially of TLR4 and EIF2AK2, but the reverse situation occurred in the PDK1 gene. The PTEN gene, whose major function was to buffer the PI3K signaling, showed down-regulation primarily at 12 h and 24 h. In addition, microarray analysis of lung tissue showed that some inactive proapoptotic factors (e.g., BAD, caspase-9, GSK-3β and FOXO) exhibited down-regulation from 12 to 24 h. Compared with mock-infected control, NF-κB gene was up-regulated at early phrase, but down-regulated on day 14. However, anti-apoptotic Bcl-2 family members, such as BCL2 and Bcl-xL genes exhibited sustained up-regulation starting from 6 h to 14 days. The mammalian target of rapamycin (mTOR), a circular antitumor target, which belonged to the PI3K-related protein kinase family, assembles into two complexes (mTORC1 and mTORC2) with different downstream effects. The p70S6K and EIF4EBP1 genes, two important substrates of mTORC1, showed elevated expression levels at 6 h.p.i and 24 h.p.i, respectively. However, the substrates of mTORC2 (e.g., Akt, SGK and PKC gene) exhibited expression levels during viral infection (Figure 4), implying a possible positive regulatory role. Here, transcriptomic analysis of HPAI H5N1-infected monkey lungs showed that certain PI3K-related genes are up-regulated. Nevertheless, it has yet to be established whether or not such up-regulation is directly caused by H5N1 AVI infection or the stable expression of NS1.

## Discussion

Reassortment occurs readily when a host cell or an animal is infected with two or more viruses and plays a prominent role in the virulence of the segmented influenza viruses [11]. The Chinese live markets, with rampant mixing of species including poultry and wildfowl, are ideal breeding grounds for genetic reassortment. In this study, we reassigned HPAI H5N1 viruses into four distinct groups and further classified the reassortant viruses into three subgroups. For R1 subgroup, the results obtained by several phylogenetic methods are in complete agreement that three gene segments (HA, NA and NP) originated from the Qinghai-like lineage and other segments descended from the Xinjiang-like viruses sampled from 2005 to 2006. Some discrepancies are observed between HA and the remaining seven segments in the R2 subgroup. The reassortant strains in the R3 subgroup resulted from acquiring genome segments from the group 3 or group 4 viruses (Figure 1 and Figure S1). In this study, we have confirmed the fluidity of the influenza virus gene pool by phylogenetic analyses. Although three reassortant subgroups were identified here, the exact number of reassortment events remains unclear due to frequent reports of H5N1 reassortment events in China [71]. Previous studies have demonstrated that some reassortants were found to be of high pathogenicity in chickens and ducks, which subsequently led to a virulence shift in avian influenza outbreak and the enhanced transmissibility between virus and host [18].

Given that mosaic genome structures can lead to significant topological incongruence during phylogenetic analyses and may influence the evolutionary analyses of genetic data [50], there is an urgent need to explore the mosaic structures within HPAI H5N1. Using a suite of approaches, we provide evidence that HPAI H5N1 viruses in China may undergo homologous recombination and found that the majority of mosaic sequences obtained from terrestrial birds were confirmed in RNP segments. It is also interesting to note that the geographic distribution of eleven recombinant viruses identified in this study was uneven, with six from eastern China and the remaining five from other regions (Table 1). To our knowledge, four recombinant isolates of HPAI H5N1 sampled from avian hosts, namely CK/JX/25/04, CK/SD/A-1/09, CK/GZ/7/08 and DK/EC/108/08, have not been previously reported and the fitness of these viruses are still unknown. Here, we identify 11 mosaic influenza sequences using phylogeny-based analysis, but it remains controversial whether these mosaic sequences represent natural homologous recombination [50].

Generally, regions of higher genetic recombination have higher levels of polymorphism [72]. In this study, genetic polymorphism analysis and neutrality tests for genomic datasets showed that the polymerase complex required for the transcription and replication of the viral genome, was characterized by high diversity and low ω (see Materials and Methods for details). This high diversity suggests a population in expansion rather than a positive selection. However, three gene segments (HA, NA and NS1) exhibited similar population dynamics, which have both higher dN/dS ratio and variability than other genes. The higher dN/dS ratio of NS1 (mean dN/dS  = 0.434) most likely reflect host immune system selective pressure that is antagonizing the IFN-induced host antiviral responses [73]. Furthermore, as a membrane ion channel protein, a higher dN/dS ratio for M2 compared with other internal proteins is expected. Despite the fact that the global ω for all gene segments was below 1, the site-specific selection analysis which is helpful in antiviral drug screening and vaccination showed that a number of positively selected sites were detected in the majority of gene segments, especially in surface glycoproteins and NS1 (Table 2). In contrast to higher dN/dS ratio and variability identified in HA, NA and NS1genes, strong conservation of amino acid sequence was observed in the remaining internal segments. These results suggest that genes with less selective pressure are more conducive to fixing advantageous mutations. In addition, sequence-based analysis showed that variation located in the ED (position 212–230), possibly due to structure requirement. Moreover, the site-by-site analysis revealed that most of positively selected sites were also seen in the ED (10/11), whose ω value was significantly higher than the RBD, suggesting that a higher selection intensity may operate on this region.

The evolutionary dynamics of a specific gene segment is valuable in understanding the structure-function relationships of that gene. In this study, our sequence analysis found that allele A of the NS1 protein differed from allele B by over 35% of their amino acids. Furthermore, early studies also suggested that NS1 protein can act as an essential determinant for influenza virus pathogenesis in a species-specific manner [74]. Residues from 81 to 113 in the ED form a trimeric complex to recruit the eukaryotic translation initiation factor 4F (eIF4F), and enhance the translation of viral mRNA [75]. Intriguingly, the amino acid composition within this region is relatively conserved for both allele A and allele B. Further analysis showed that the H5N1 viruses circulating in China have nine distinct C-terminal motifs in NS1, and the conserved PL motif ESEV accounted for 70.1% (324/462) of viruses in this study. Previous experiments suggested that the PL motif of HPAI H5N1 increased viral virulence in mice [76], while other studies demonstrated that this motif modulated viral replication in a strain- and host-dependent manner [77]. Infections with HPAI H5N1 viruses can induce a variety of intracellular signaling pathways and gene expression events. In particular, PI3K signaling, which can be activated by the viral NS1 protein during the late phase of the infection cycle, is involved in a wide variety of cellular signaling events [70]. The NS1 protein of HPAI H5N1 has several SH binding motifs that are required for interaction with cellular proteins [30]. Here, we demonstrated that the NS1 gene of H5N1 virus confers high levels of cytokine expression in macaque lung. Transcription analyses also revealed down-regulation of genes involved in the negative regulation of the PI3K/Akt signaling (e.g., PTEN, BAD, caspase-9, FOXO and GSK-3β) starting from 12 to 24 h. As shown in Figure 4, NF-κB gene was up-regulated early, indicating that NF-κB plays an important role in the antiviral response to H5N1 virus infection. However, the down-regulation of NF-κB gene was observed on day 14.p.i and this can be explained by the fact that H5N1 NS1 protein exerts great influence on disease pathogenesis through inhibiting the IKK-mediated NF-κB activation and production. Collectively, these results demonstrate that the PI3K/Akt signaling pathway are crucial for viral replication and co-activation of the antiviral response.

In summary, the fluidity of the influenza virus gene pool was responsible for the maintenance of H5N1 reassortants in China. The frequent reassortment of RNP subunits observed in the H5N1 viruses from China indicated their viral fitness landscape is determined by functional viability, with less selective pressure to fix advantageous mutations. We concluded that the immune selection pressure conferred both high variability and dN/dS ratio on the NS1 protein. In addition, most of positively selected sites were seen in the ED (10/11) of NS1, suggesting that a higher selection intensity may operate on this region. HPAI H5N1 has been endemic in poultry populations and evolved into diversified lineages in China. These viruses not only continue to circulate in avian species, but occasionally transmit to humans. Therefore, we suggested that it is imperative to make thorough preparations to update candidate vaccines for H5N1 virus as well as to conduct ongoing surveillance in domestic poultry and wild birds.

## Supporting Information

Figure S1 **Phylogenetic trees of H5N1 influenza viruses sampled from 1996–2012.** ML phylogenies reconstructed from (A) PB2 gene; (B) PB1 gene; (C) PA gene; (D) HA gene; (E) NP gene; (F) NA gene; (G) MP gene; (H) M1 gene; (I) M2 gene; (J) NS gene; (K) NS1 gene; (L) NS2 gene. Topology supports summarized from 100 ML bootstrap replications are shown. For major lineages, NJ bootstrap (100 replications) and posterior probability from BMCMC analyses (5000 tree) are shown for key nodes (ML/NJ/BMCMC). Putative recombinant viruses are designated by magenta circles. Reassortant subgroups (R1, R2 and R3) are indicated with solid lines. Arrows indicate the roots, and scale bars represent nucleotide substitutions per site.
(PDF)

Figure S2 **The structure features of H5N1 non-structural protein NS1.** (A) Phylogenetic analysis of the NS1 gene based on 462 nucleotide sequences of HPAI H5N1 isolates. (B) The NS1 amino acid sequence alignment for the four viruses (AH/2/05, CK/GD/1/05, HK/156/97 and GS/GD/1/96). The box indicates the previously identified important amino acid residues of NS1 protein. (C) Structural alignment of four H5N1 NS1 RBD (AH/2/05 (pink), CK/GD/1/05 (light green), HK/156/97 (salmon) and GS/GD/1/96 (sky blue)) with A/crow/Kyoto/T1/2004 (tan) H5N1 NS1 RBD. The amino acid residues at position 38 and 41 are labeled. (D) F3-binding pocket on NS1A (85-215). A hydrophobic pocket on the NS1A surface binds to the F3 Zn finger of F2F3. The NS1A amino acid residues presented by their molecular surface interact with the aromatic side chains of residues Y97, F98, and F102 of the F3 Zn finger of F2F3. (E) Schematic illustration of the binding domain structure of NS1 and two subunits of PI3K (p85β and p110). The same color coding is used throughout this article unless specified. Gray regions are linkers between domains. (F) Ribbon diagram of the NS1-p85β complex (Protein Data Bank code: 2V1Y for p85α iSH2 and 2GX9 for NS1) (G) Ribbon diagram of the NS1- p85β-p110 complex (Protein Data Bank code: 2RD0).
(PDF)

Figure S3 **Phylogenetic analysis of the NS1 gene based on 462 nucleotide sequences of HPAI H5N1 isolates.** A small branch of the A allele contained the sequence AIASS at position 80–84 is highlighted in green.
(PDF)

Table S1 **H5N1 influenza viruses used in this study and their GenBank accession numbers.**
(DOC)

Table S2 **Pairwise intergroup distance of HA gene.**
(DOC)

Table S3 **Sequence information and phylogenetic groupings of sequences used in this study.**
(DOC)

Table S4 **Estimates of polymorphism and neutrality tests.**
(DOC)

Table S5 **Accession numbers of 462 NS1 sequences used in this study.**
(DOC)

Table S6 **Amino acid polymorphisms of the non-structure 1 protein (NS1) sequences of H5N1 viruses.** The consensus sequence of the non-structure 1 protein (NS1) sequences of H5N1 viruses is shown in the right column. The 20 amino acids and gaps present in amino acid sequences are shown along the top of table. Orange square represents each occasion that a particular amino acid is found at that position in the sequence with a frequency greater than 1%. The total diversity at each position is shown in the column titled SUM. Grey represents invariant position. Yellow represents position where two alternative amino acids are found. Green represents position at which three alternative amino acids are found. Blue represents position at which four and red five or greater alternative amino acids are found.
(DOC)

Table S7 **Distribution of PL motifs in 462 influenza NS1 protein sequences.** PDZ-domain ligand sequences are listed in the PL column and the distribution of each PL sequence in avian and mammalian isolates is shown.
(DOC)

Table S8 **A list of selected genes involved in PI3K signaling.**
(DOC)
