# Supplementary material for: Genetic Dynamic Analysis of the Influenza A H5N1 NS1 Gene in China
Source: PLoS One. 2014 Jul 8;9(7):e101384. doi: 10.1371/journal.pone.0101384 (PMC4086889; doi:10.1371/journal.pone.0101384)
Supplement: Figure S1 — Phylogenetic trees of H5N1 influenza viruses sampled from 1996–2012. ML phylogenies reconstructed from (A) PB2 gene; (B) PB1 gene; (C) PA gene; (D) HA gene; (E) NP gene; (F) NA gene; (G) MP gene; (H) M1 gene; (I) M2 gene; (J) NS gene; (K) NS1 gene; (L) NS2 gene. Topology supports summarized from 100 ML bootstrap replications are shown. For major lineages, NJ bootstrap (100 replications) and posterior probability from BMCMC analyses (5000 tree) are shown for key nodes (ML/NJ/BMCMC). Putative recombinant viruses are designated by magenta circles. Reassortant subgroups (R1, R2 and R3) are indicated with solid lines. Arrows indicate the roots, and scale bars represent nucleotide substitutions per site. (PDF) [file pone.0101384.s001.pdf]

**Figure S1 Phylogenetic trees of H5N1 influenza viruses sampled from 1996-2012.** ML phylogenies reconstructed from (A) PB2 gene; (B) PB1 gene; (C) PA gene; (D) HA gene; (E) NP gene; (F) NA gene; (G) MP gene; (H) M1 gene; (I) M2 gene; (J) NS gene; (K) NS1 gene; (L) NS2 gene. Topology supports summarized from 100 ML bootstrap replications are shown. For major lineages, NJ bootstrap (100 replications) and posterior probability from BMCMC analyses (5000 tree) are shown for key nodes (ML/NJ/BMCMC). Putative recombinant viruses are designated by magenta circles. Reassortant subgroups (R1, R2 and R3) are indicated with solid lines. Arrows indicate the roots, and scale bars represent nucleotide substitutions per site.

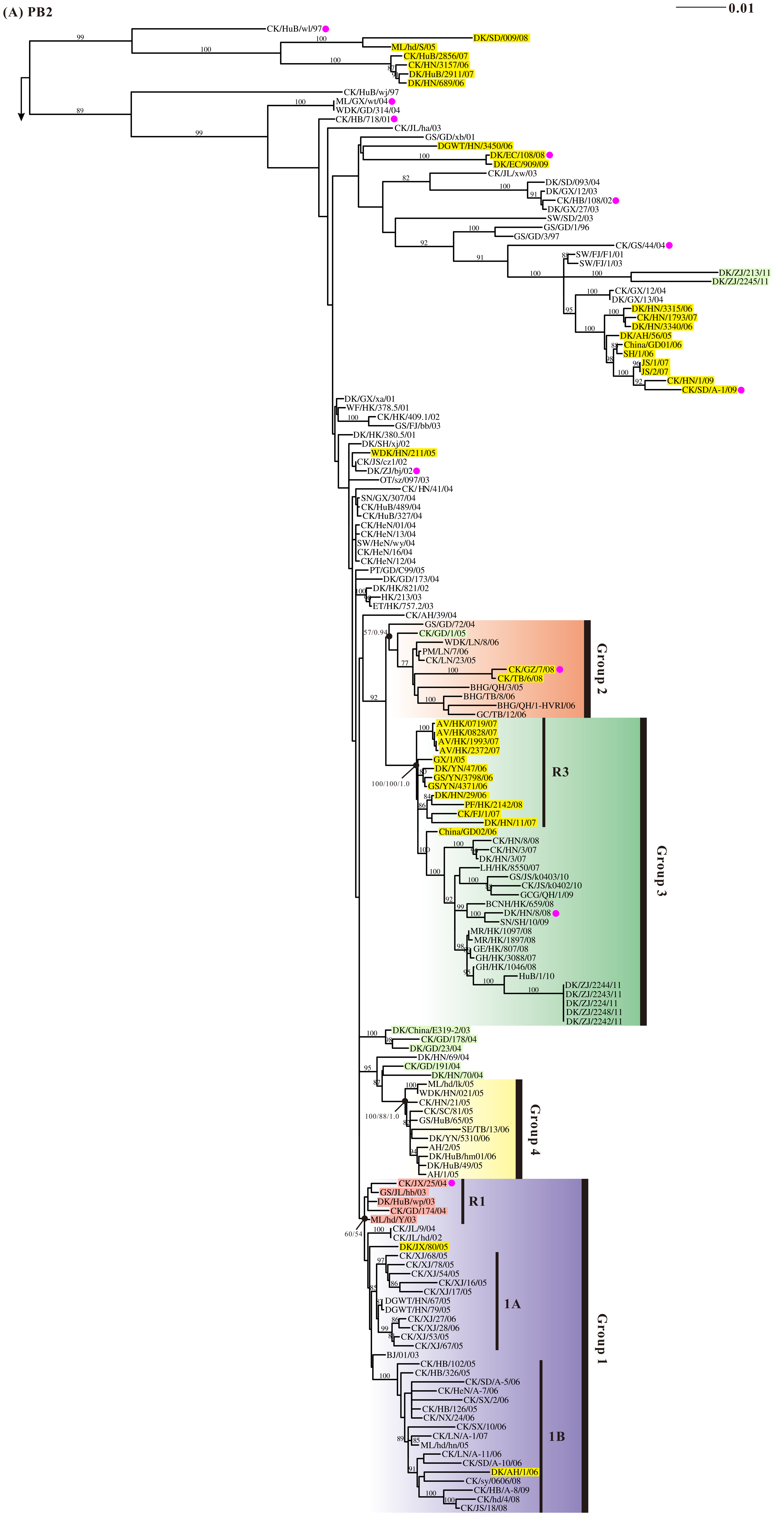

**(B) PB1**

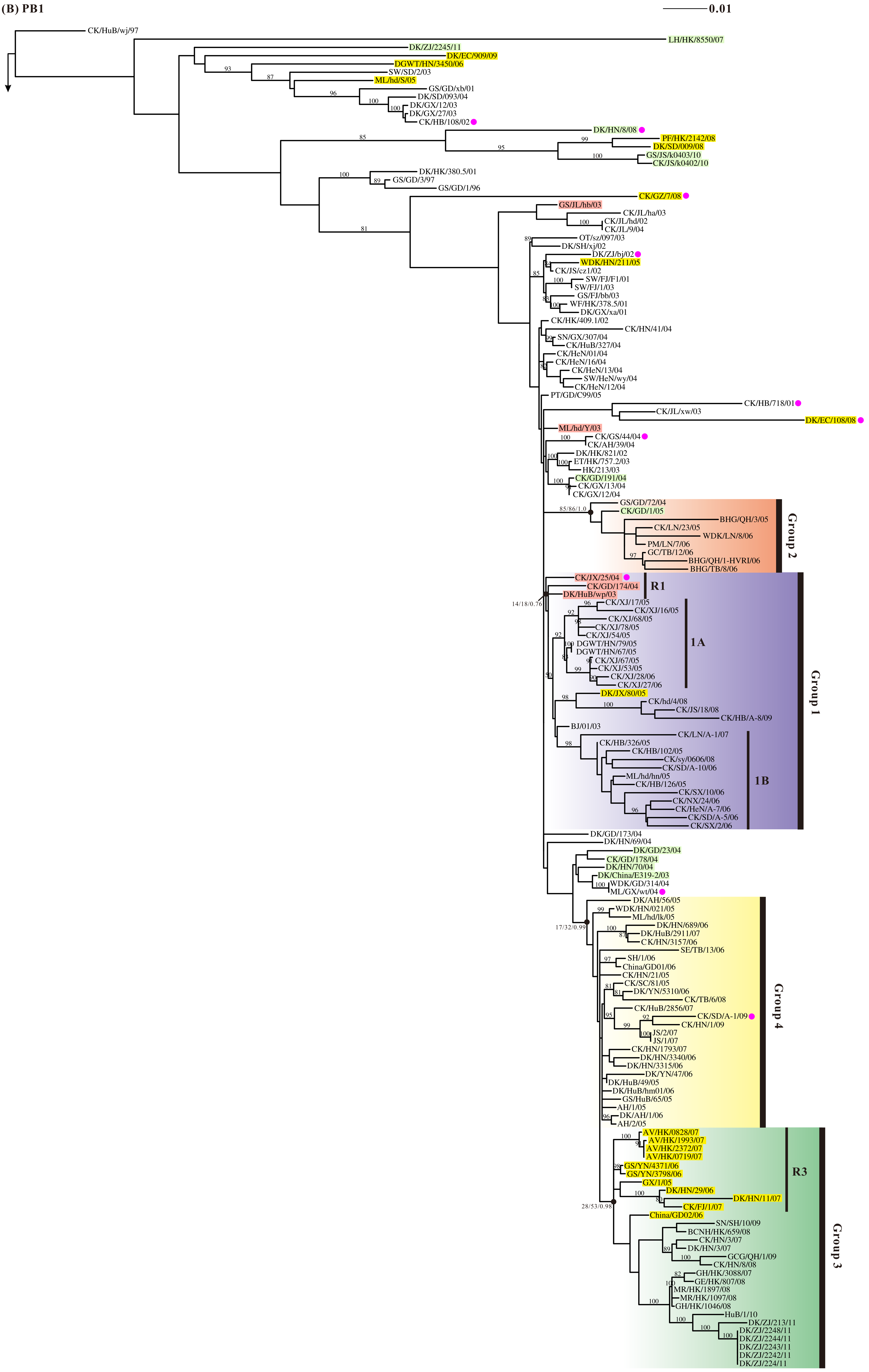
0.01
CK/HuB/wj/97
LH/HK/8550/07
DK/ZJ/2245/11
DK/EC/909/09
93
DGWT/HN/3450/06
SW/SD/2/03
87
ML/hd/S/05
96
GS/GD/xb/01
DK/SD/093/04
100
DK/GX/12/03
100
DK/GX/27/03
CK/HB/108/02
DK/HN/8/08
85
99
PF/HK/2142/08
95
DK/SD/009/08
100
GS/JS/k0403/10
CK/JS/k0402/10
100
DK/HK/380.5/01
89
GS/GD/3/97
GS/GD/1/96
CK/GZ/7/08
81
GS/JL/hb/03
CK/JL/ha/03
100
CK/JL/hd/02
CK/JL/9/04
89
OT/sz/097/03
DK/SH/xj/02
DK/ZJ/bj/02
WDK/HN/211/05
85
CK/JS/cz1/02
100
SW/FJ/F1/01
SW/FJ/1/03
GS/FJ/bb/03
100
WF/HK/378.5/01
DK/GX/xa/01
CK/HK/409.1/02
CK/HN/41/04
99
SN/GX/307/04
CK/HuB/327/04
CK/HeN/01/04
CK/HeN/16/04
CK/HeN/13/04
SW/HeN/wy/04
CK/HeN/12/04
PT/GD/C99/05
CK/HB/718/01
CK/JL/xw/03
DK/EC/108/08
ML/hd/Y/03
100
CK/GS/44/04
CK/AH/39/04
100
DK/HK/821/02
100
ET/HK/757.2/03
HK/213/03
100
CK/GD/191/04
CK/GX/13/04
CK/GX/12/04
85/86/1.0
GS/GD/72/04
CK/GD/1/05
BHG/QH/3/05
CK/LN/23/05
WDK/LN/8/06
PM/LN/7/06
97
GC/TB/12/06
BHG/QH/1-HVRI/06
BHG/TB/8/06
Group 2
CK/JX/25/04
CK/GD/174/04
R1
DK/HuB/wp/03
14/18/0.76
96
CK/XJ/17/05
CK/XJ/16/05
92
CK/XJ/68/05
CK/XJ/78/05
92
CK/XJ/54/05
100
DGWT/HN/79/05
1A
DGWT/HN/67/05
CK/XJ/67/05
99
CK/XJ/53/05
50
CK/XJ/28/06
CK/XJ/27/06
DK/JX/80/05
98
CK/hd/4/08
100
CK/JS/18/08
CK/HB/A-8/09
Group 1
BJ/01/03
CK/LN/A-1/07
98
CK/HB/326/05
CK/HB/102/05
CK/sy/0606/08
CK/SD/A-10/06
ML/hd/hn/05
1B
CK/HB/126/05
CK/SX/10/06
CK/NX/24/06
96
CK/HeN/A-7/06
CK/SD/A-5/06
CK/SX/2/06
DK/GD/173/04
DK/HN/69/04
DK/GD/23/04
CK/GD/178/04
DK/HN/70/04
DK/China/E319-2/03
100
WDK/GD/314/04
ML/GX/wt/04
DK/AH/56/05
99
WDK/HN/021/05
ML/hd/lk/05
17/32/0.99
100
DK/HN/689/06
87
DK/HuB/2911/07
CK/HN/3157/06
SE/TB/13/06
97
SH/1/06
China/GD01/06
CK/HN/21/05
81
CK/SC/81/05
81
DK/YN/5310/06
CK/TB/6/08
Group 4
CK/HuB/2856/07
95
92
CK/SD/A-1/09
99
CK/HN/1/09
100
JS/2/07
JS/1/07
CK/HN/1793/07
DK/HN/3340/06
DK/HN/3315/06
DK/YN/47/06
DK/HuB/49/05
DK/HuB/hm01/06
GS/HuB/65/05
AH/1/05
96
DK/AH/1/06
AH/2/05
100
AV/HK/0828/07
AV/HK/1993/07
AV/HK/2372/07
AV/HK/0719/07
98
GS/YN/4371/06
R3
GS/YN/3798/06
GX/1/05
100
DK/HN/29/06
80
DK/HN/11/07
CK/FJ/1/07
28/53/0.98
China/GD02/06
SN/SH/10/09
BCNH/HK/659/08
CK/HN/3/07
89
DK/HN/3/07
100
GCG/QH/1/09
CK/HN/8/08
Group 3
82
GH/HK/3088/07
100
GE/HK/807/08
MR/HK/1897/08
100
MR/HK/1097/08
GH/HK/1046/08
HuB/1/10
100
DK/ZJ/213/11
100
DK/ZJ/2248/11
100
DK/ZJ/2244/11
DK/ZJ/2243/11
DK/ZJ/2242/11
DK/ZJ/224/11

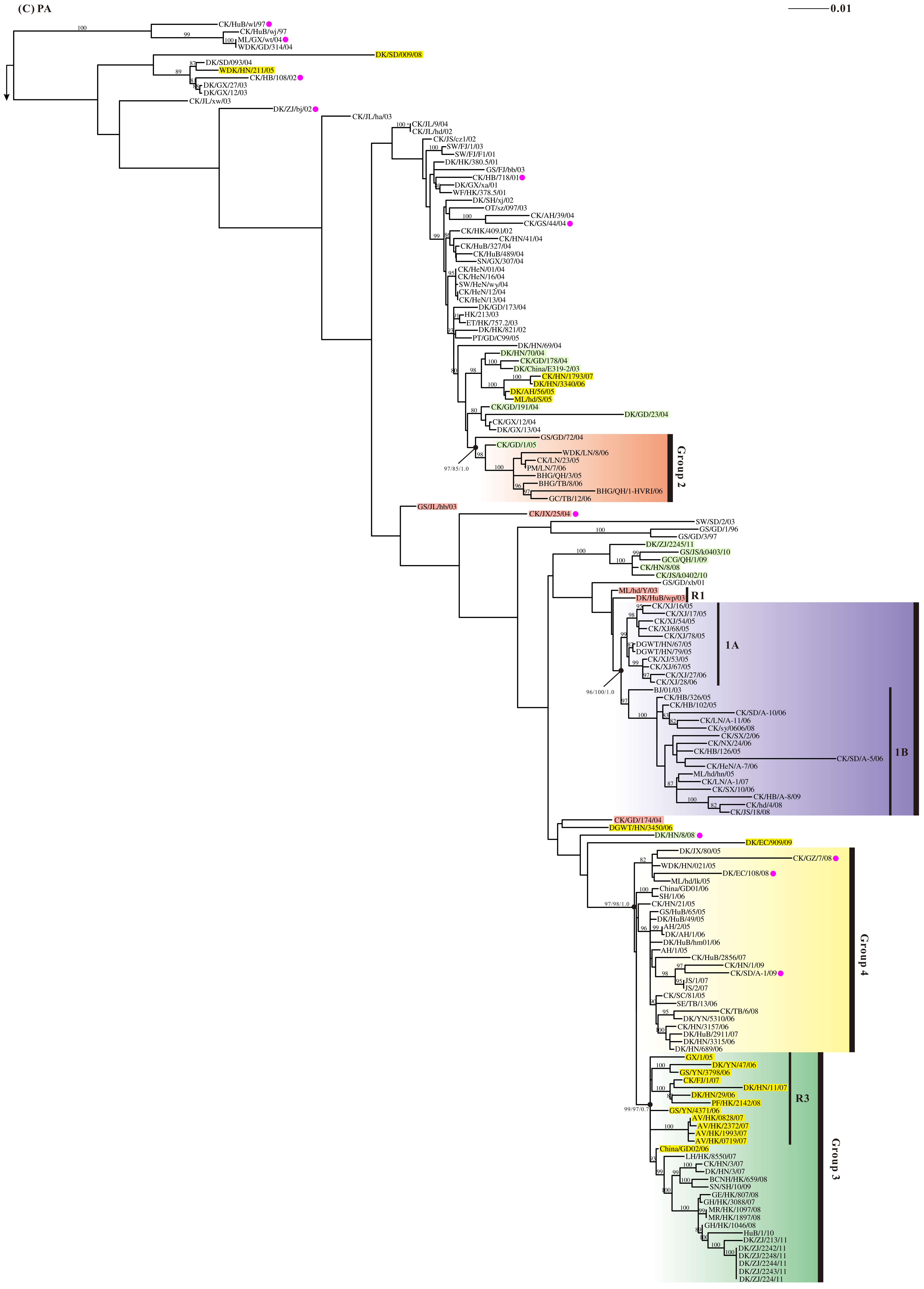

(C) PA
0.01
CK/HuB/wl/97
CK/HuB/wj/97
ML/GX/wt/04
WDK/GD/314/04
DK/SD/009/08
DK/SD/093/04
WDK/HN/211/05
CK/HB/108/02
DK/GX/27/03
DK/GX/12/03
CK/JL/xw/03
DK/ZJ/bj/02
CK/JL/ha/03
CK/JL/9/04
CK/JL/hd/02
CK/JS/cz1/02
SW/FJ/1/03
SW/FJ/F1/01
DK/HK/380.5/01
GS/FJ/bb/03
CK/HB/718/01
DK/GX/xa/01
WF/HK/378.5/01
DK/SH/xj/02
OT/sz/097/03
CK/AH/39/04
CK/GS/44/04
CK/HK/409.1/02
CK/HN/41/04
CK/HuB/327/04
CK/HuB/489/04
SN/GX/307/04
CK/HeN/01/04
CK/HeN/16/04
SW/HeN/wy/04
CK/HeN/12/04
CK/HeN/13/04
DK/GD/173/04
HK/213/03
ET/HK/757.2/03
DK/HK/821/02
PT/GD/C99/05
DK/HN/69/04
DK/HN/70/04
CK/GD/178/04
DK/China/E319-2/03
CK/HN/1793/07
DK/HN/3340/06
DK/AH/56/05
ML/hd/S/05
CK/GD/191/04
DK/GD/23/04
CK/GX/12/04
DK/GX/13/04
GS/GD/72/04
CK/GD/1/05
WDK/LN/8/06
CK/LN/23/05
PM/LN/7/06
BHG/QH/3/05
BHG/TB/8/06
BHG/QH/1-HVRI/06
GC/TB/12/06
97/85/1.0
Group 2
GS/JL/hb/03
CK/JX/25/04
SW/SD/2/03
GS/GD/1/96
GS/GD/3/97
DK/ZJ/2245/11
GS/JS/k0403/10
GCG/QH/1/09
CK/HN/8/08
CK/JS/k0402/10
GS/GD/xb/01
ML/hd/Y/03
DK/HuB/wp/03
R1
CK/XJ/16/05
CK/XJ/17/05
CK/XJ/54/05
CK/XJ/68/05
CK/XJ/78/05
DGWT/HN/67/05
DGWT/HN/79/05
CK/XJ/53/05
CK/XJ/67/05
CK/XJ/27/06
CK/XJ/28/06
1A
96/100/1.0
BJ/01/03
CK/HB/326/05
CK/HB/102/05
CK/SD/A-10/06
CK/LN/A-11/06
CK/sy/0606/08
CK/SX/2/06
CK/NX/24/06
CK/HB/126/05
CK/SD/A-5/06
CK/HeN/A-7/06
ML/hd/hn/05
CK/LN/A-1/07
CK/SX/10/06
CK/HB/A-8/09
CK/hd/4/08
CK/JS/18/08
1B
Group 1
CK/GD/174/04
DGWT/HN/3450/06
DK/HN/8/08
DK/EC/909/09
DK/JX/80/05
CK/GZ/7/08
WDK/HN/021/05
DK/EC/108/08
ML/hd/lk/05
China/GD01/06
SH/1/06
CK/HN/21/05
GS/HuB/65/05
DK/HuB/49/05
AH/2/05
DK/AH/1/06
DK/HuB/hm01/06
AH/1/05
CK/HuB/2856/07
CK/HN/1/09
CK/SD/A-1/09
JS/1/07
JS/2/07
CK/SC/81/05
SE/TB/13/06
CK/TB/6/08
DK/YN/5310/06
CK/HN/3157/06
DK/HuB/2911/07
DK/HN/3315/06
DK/HN/689/06
97/98/1.0
Group 4
GX/1/05
DK/YN/47/06
GS/YN/3798/06
CK/FJ/1/07
DK/HN/11/07
DK/HN/29/06
PF/HK/2142/08
GS/YN/4371/06
AV/HK/0828/07
AV/HK/2372/07
AV/HK/1993/07
AV/HK/0719/07
R3
99/97/0.7
China/GD02/06
LH/HK/8550/07
CK/HN/3/07
DK/HN/3/07
BCNH/HK/659/08
SN/SH/10/09
GE/HK/807/08
GH/HK/3088/07
MR/HK/1097/08
MR/HK/1897/08
GH/HK/1046/08
HuB/1/10
DK/ZJ/213/11
DK/ZJ/2242/11
DK/ZJ/2248/11
DK/ZJ/2244/11
DK/ZJ/2243/11
DK/ZJ/224/11
Group 3

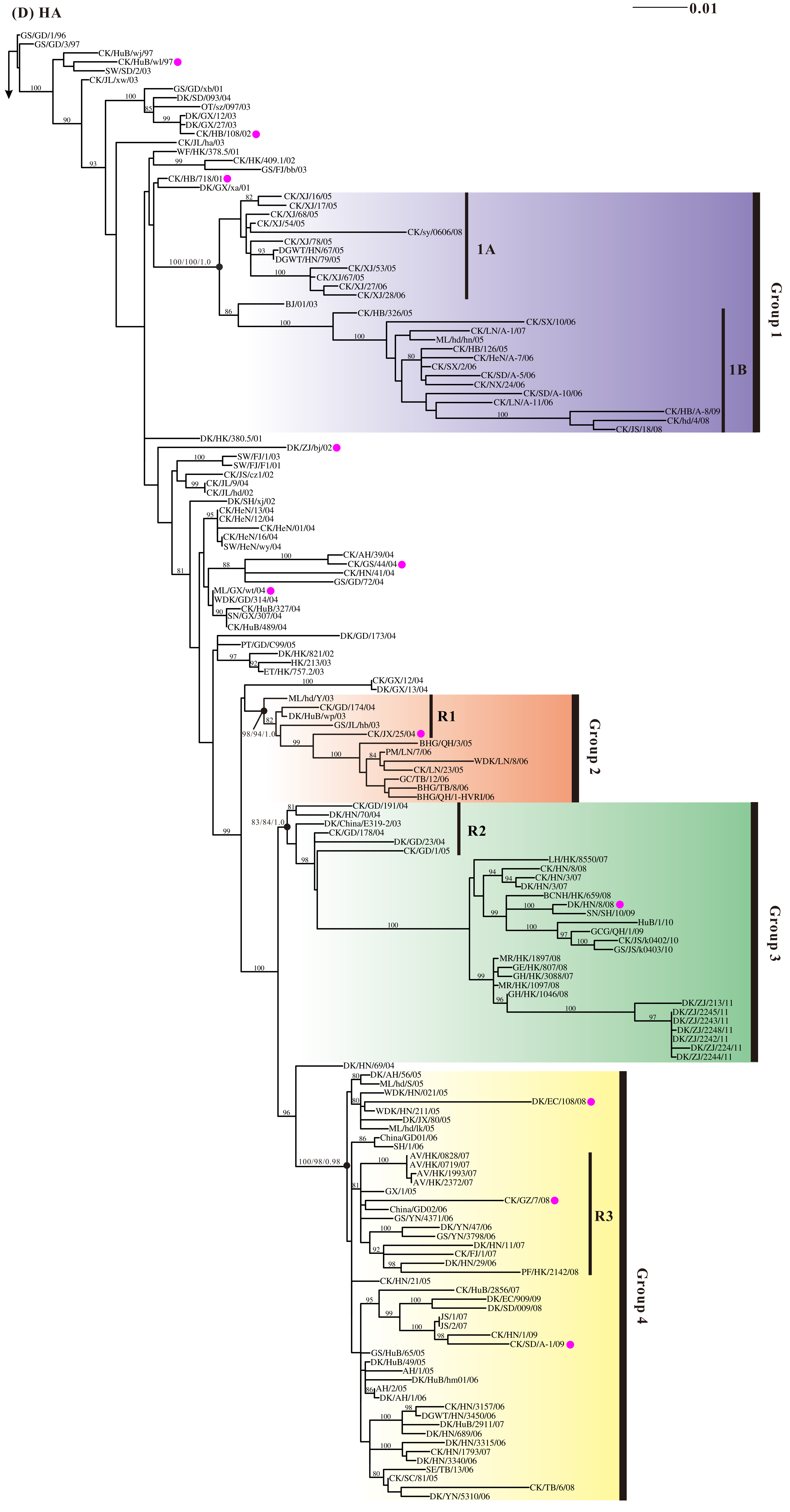

(D) HA
0.01
GS/GD/1/96
GS/GD/3/97
CK/HuB/wj/97
CK/HuB/wl/97
SW/SD/2/03
CK/JL/xw/03
GS/GD/xb/01
DK/SD/093/04
OT/sz/097/03
DK/GX/12/03
DK/GX/27/03
CK/HB/108/02
CK/JL/ha/03
WF/HK/378.5/01
CK/HK/409.1/02
GS/FJ/bb/03
CK/HB/718/01
DK/GX/xa/01
CK/XJ/16/05
CK/XJ/17/05
CK/XJ/68/05
CK/XJ/54/05
CK/sy/0606/08
CK/XJ/78/05
DGWT/HN/67/05
DGWT/HN/79/05
CK/XJ/53/05
CK/XJ/67/05
CK/XJ/27/06
CK/XJ/28/06
1A
BJ/01/03
CK/HB/326/05
CK/SX/10/06
CK/LN/A-1/07
ML/hd/hn/05
CK/HB/126/05
CK/HeN/A-7/06
CK/SX/2/06
CK/SD/A-5/06
CK/NX/24/06
CK/SD/A-10/06
CK/LN/A-11/06
CK/HB/A-8/09
CK/hd/4/08
CK/JS/18/08
1B
Group 1
100/100/1.0
DK/HK/380.5/01
DK/ZJ/bj/02
SW/FJ/1/03
SW/FJ/F1/01
CK/JS/cz1/02
CK/JL/9/04
CK/JL/hd/02
DK/SH/xj/02
CK/HeN/13/04
CK/HeN/12/04
CK/HeN/01/04
CK/HeN/16/04
SW/HeN/wy/04
CK/AH/39/04
CK/GS/44/04
CK/HN/41/04
GS/GD/72/04
ML/GX/wt/04
WDK/GD/314/04
CK/HuB/327/04
SN/GX/307/04
CK/HuB/489/04
DK/GD/173/04
PT/GD/C99/05
DK/HK/821/02
HK/213/03
ET/HK/757.2/03
CK/GX/12/04
DK/GX/13/04
ML/hd/Y/03
CK/GD/174/04
DK/HuB/wp/03
GS/JL/hb/03
CK/JX/25/04
R1
BHG/QH/3/05
PM/LN/7/06
WDK/LN/8/06
CK/LN/23/05
GC/TB/12/06
BHG/TB/8/06
BHG/QH/1-HVRI/06
Group 2
98/94/1.0
CK/GD/191/04
DK/HN/70/04
DK/China/E319-2/03
CK/GD/178/04
DK/GD/23/04
CK/GD/1/05
R2
83/84/1.0
LH/HK/8550/07
CK/HN/8/08
CK/HN/3/07
DK/HN/3/07
BCNH/HK/659/08
DK/HN/8/08
SN/SH/10/09
HuB/1/10
GCG/QH/1/09
CK/JS/k0402/10
GS/JS/k0403/10
MR/HK/1897/08
GE/HK/807/08
GH/HK/3088/07
MR/HK/1097/08
GH/HK/1046/08
DK/ZJ/213/11
DK/ZJ/2245/11
DK/ZJ/2243/11
DK/ZJ/2248/11
DK/ZJ/2242/11
DK/ZJ/224/11
DK/ZJ/2244/11
Group 3
DK/HN/69/04
DK/AH/56/05
ML/hd/S/05
WDK/HN/021/05
DK/EC/108/08
WDK/HN/211/05
DK/JX/80/05
ML/hd/lk/05
China/GD01/06
SH/1/06
AV/HK/0828/07
AV/HK/0719/07
AV/HK/1993/07
AV/HK/2372/07
GX/1/05
CK/GZ/7/08
China/GD02/06
GS/YN/4371/06
DK/YN/47/06
GS/YN/3798/06
DK/HN/11/07
CK/FJ/1/07
DK/HN/29/06
PF/HK/2142/08
R3
CK/HN/21/05
CK/HuB/2856/07
DK/EC/909/09
DK/SD/009/08
JS/1/07
JS/2/07
CK/HN/1/09
CK/SD/A-1/09
GS/HuB/65/05
DK/HuB/49/05
AH/1/05
DK/HuB/hm01/06
AH/2/05
DK/AH/1/06
CK/HN/3157/06
DGWT/HN/3450/06
DK/HuB/2911/07
DK/HN/689/06
DK/HN/3315/06
CK/HN/1793/07
DK/HN/3340/06
SE/TB/13/06
CK/SC/81/05
CK/TB/6/08
DK/YN/5310/06
Group 4
100/98/0.98

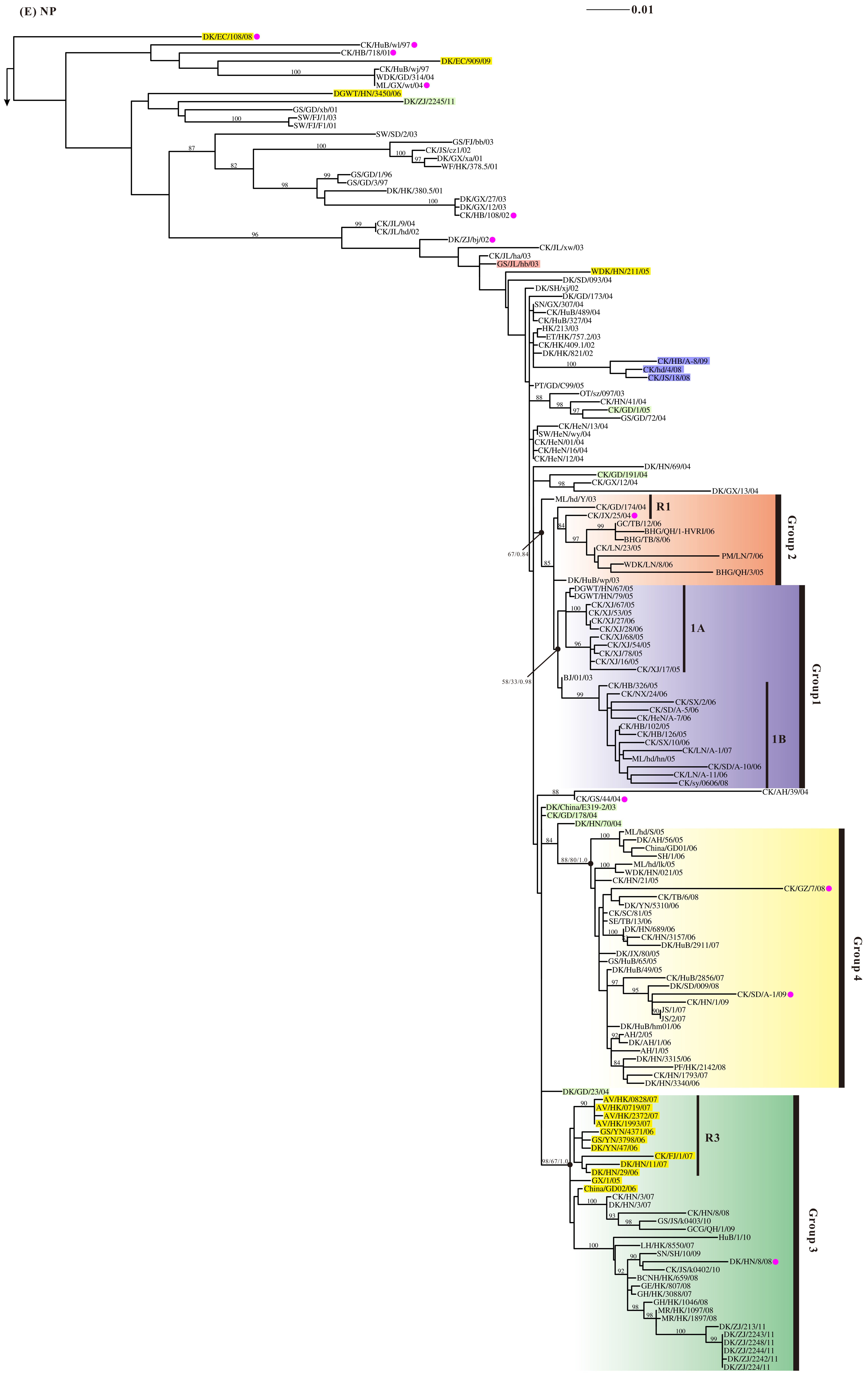

(E) NP
0.01
DK/EC/108/08
CK/HuB/wl/97
CK/HB/718/01
DK/EC/909/09
100
CK/HuB/wj/97
WDK/GD/314/04
ML/GX/wt/04
DGWT/HN/3450/06
DK/ZJ/2245/11
GS/GD/xb/01
100
SW/FJ/1/03
SW/FJ/F1/01
SW/SD/2/03
87
100
GS/FJ/bb/03
100
CK/JS/cz1/02
97
DK/GX/xa/01
WF/HK/378.5/01
82
99
GS/GD/1/96
98
GS/GD/3/97
DK/HK/380.5/01
100
DK/GX/27/03
DK/GX/12/03
CK/HB/108/02
99
CK/JL/9/04
CK/JL/hd/02
96
DK/ZJ/bj/02
CK/JL/xw/03
CK/JL/ha/03
GS/JL/hb/03
WDK/HN/211/05
DK/SD/093/04
DK/SH/xj/02
DK/GD/173/04
SN/GX/307/04
CK/HuB/489/04
CK/HuB/327/04
HK/213/03
ET/HK/757.2/03
CK/HK/409.1/02
DK/HK/821/02
100
CK/HB/A-8/09
CK/hd/4/08
CK/JS/18/08
PT/GD/C99/05
88
OT/sz/097/03
98
CK/HN/41/04
97
CK/GD/1/05
GS/GD/72/04
CK/HeN/13/04
SW/HeN/wy/04
CK/HeN/01/04
CK/HeN/16/04
CK/HeN/12/04
DK/HN/69/04
CK/GD/191/04
98
CK/GX/12/04
DK/GX/13/04
ML/hd/Y/03
CK/GD/174/04
R1
CK/JX/25/04
84
99
GC/TB/12/06
BHG/QH/1-HVRI/06
BHG/TB/8/06
97
CK/LN/23/05
PM/LN/7/06
WDK/LN/8/06
BHG/QH/3/05
Group 2
67/0.84
85
DK/HuB/wp/03
DGWT/HN/67/05
DGWT/HN/79/05
100
CK/XJ/67/05
CK/XJ/53/05
CK/XJ/27/06
CK/XJ/28/06
1A
CK/XJ/68/05
96
CK/XJ/54/05
CK/XJ/78/05
CK/XJ/16/05
CK/XJ/17/05
58/33/0.98
BJ/01/03
CK/HB/326/05
99
CK/NX/24/06
CK/SX/2/06
CK/SD/A-5/06
CK/HeN/A-7/06
CK/HB/102/05
CK/HB/126/05
1B
CK/SX/10/06
CK/LN/A-1/07
ML/hd/hn/05
CK/SD/A-10/06
CK/LN/A-11/06
CK/sy/0606/08
Group1
88
CK/AH/39/04
CK/GS/44/04
DK/China/E319-2/03
CK/GD/178/04
DK/HN/70/04
100
ML/hd/S/05
84
DK/AH/56/05
China/GD01/06
SH/1/06
88/80/1.0
100
ML/hd/lk/05
WDK/HN/021/05
CK/HN/21/05
CK/GZ/7/08
CK/TB/6/08
DK/YN/5310/06
CK/SC/81/05
SE/TB/13/06
100
DK/HN/689/06
CK/HN/3157/06
DK/HuB/2911/07
DK/JX/80/05
GS/HuB/65/05
DK/HuB/49/05
Group 4
CK/HuB/2856/07
97
DK/SD/009/08
95
CK/SD/A-1/09
CK/HN/1/09
90
JS/1/07
JS/2/07
DK/HuB/hm01/06
92
AH/2/05
DK/AH/1/06
AH/1/05
84
DK/HN/3315/06
PF/HK/2142/08
CK/HN/1793/07
DK/HN/3340/06
DK/GD/23/04
90
AV/HK/0828/07
AV/HK/0719/07
AV/HK/2372/07
AV/HK/1993/07
GS/YN/4371/06
R3
GS/YN/3798/06
DK/YN/47/06
98/67/1.0
CK/FJ/1/07
DK/HN/11/07
DK/HN/29/06
GX/1/05
China/GD02/06
CK/HN/3/07
100
DK/HN/3/07
CK/HN/8/08
93
98
GS/JS/k0403/10
GCG/QH/1/09
HuB/1/10
100
LH/HK/8550/07
90
SN/SH/10/09
DK/HN/8/08
92
CK/JS/k0402/10
BCNH/HK/659/08
GE/HK/807/08
GH/HK/3088/07
98
GH/HK/1046/08
98
MR/HK/1097/08
MR/HK/1897/08
Group 3
100
DK/ZJ/213/11
99
DK/ZJ/2243/11
DK/ZJ/2248/11
DK/ZJ/2244/11
DK/ZJ/2242/11
DK/ZJ/224/11

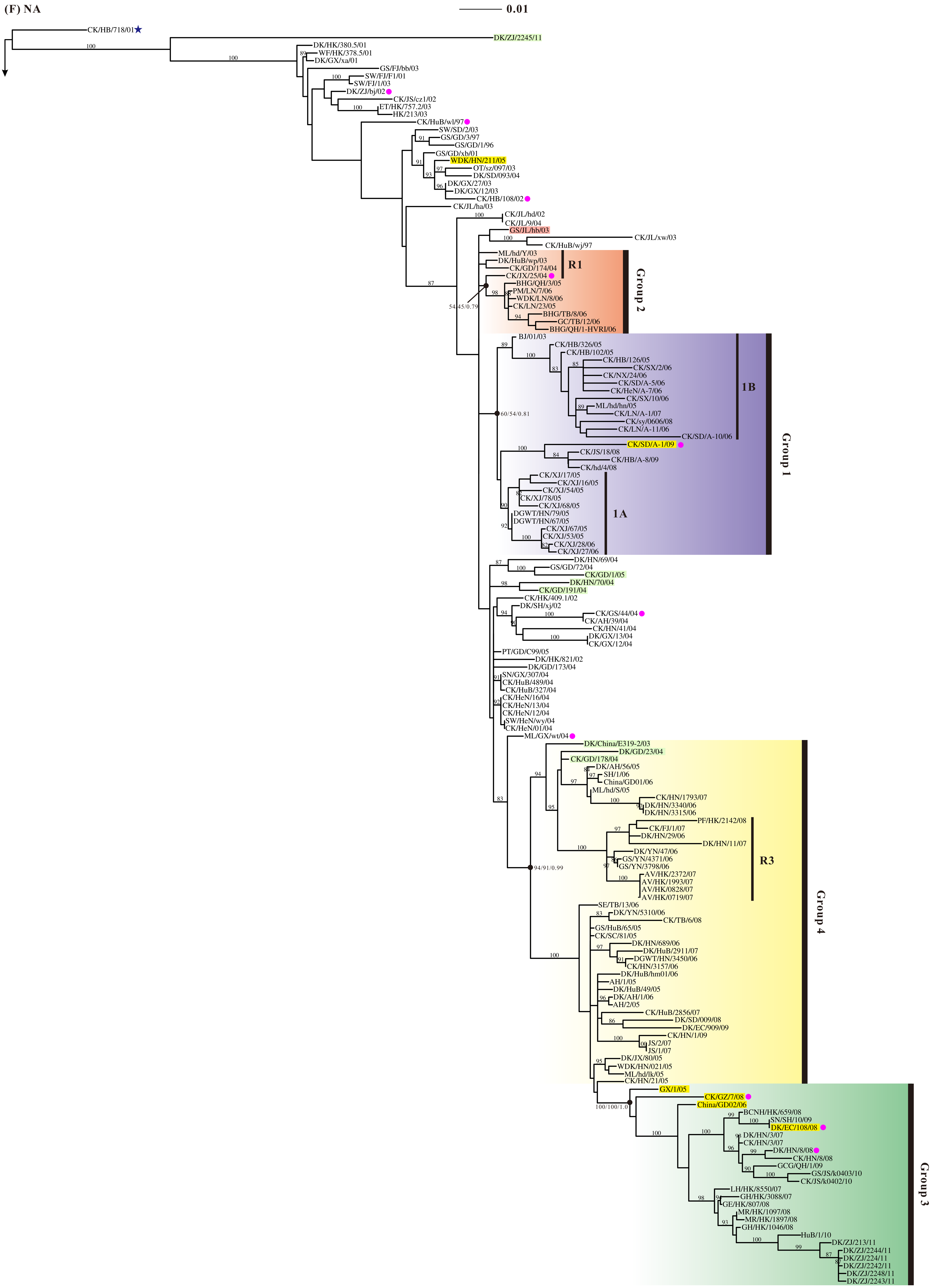
(F) NA
0.01
CK/HB/718/01
DK/ZJ/2245/11
DK/HK/380.5/01
WF/HK/378.5/01
DK/GX/xa/01
GS/FJ/bb/03
SW/FJ/F1/01
SW/FJ/1/03
DK/ZJ/bj/02
CK/JS/cz1/02
ET/HK/757.2/03
HK/213/03
CK/HuB/wl/97
SW/SD/2/03
GS/GD/3/97
GS/GD/1/96
GS/GD/xb/01
WDK/HN/211/05
OT/sz/097/03
DK/SD/093/04
DK/GX/27/03
DK/GX/12/03
CK/HB/108/02
CK/JL/ha/03
CK/JL/hd/02
CK/JL/9/04
GS/JL/hb/03
CK/JL/xw/03
CK/HuB/wj/97
ML/hd/Y/03
DK/HuB/wp/03
CK/GD/174/04
CK/JX/25/04
R1
BHG/QH/3/05
PM/LN/7/06
WDK/LN/8/06
CK/LN/23/05
BHG/TB/8/06
GC/TB/12/06
BHG/QH/1-HVRI/06
Group 2
54/45/0.79
BJ/01/03
CK/HB/326/05
CK/HB/102/05
CK/HB/126/05
CK/SX/2/06
CK/NX/24/06
CK/SD/A-5/06
CK/HeN/A-7/06
CK/SX/10/06
ML/hd/hn/05
CK/LN/A-1/07
CK/sy/0606/08
CK/LN/A-11/06
CK/SD/A-10/06
1B
60/54/0.81
CK/SD/A-1/09
CK/JS/18/08
CK/HB/A-8/09
CK/hd/4/08
CK/XJ/17/05
CK/XJ/16/05
CK/XJ/54/05
CK/XJ/78/05
CK/XJ/68/05
DGWT/HN/79/05
DGWT/HN/67/05
CK/XJ/67/05
CK/XJ/53/05
CK/XJ/28/06
CK/XJ/27/06
1A
Group 1
DK/HN/69/04
GS/GD/72/04
CK/GD/1/05
DK/HN/70/04
CK/GD/191/04
CK/HK/409.1/02
DK/SH/xj/02
CK/GS/44/04
CK/AH/39/04
CK/HN/41/04
DK/GX/13/04
CK/GX/12/04
PT/GD/C99/05
DK/HK/821/02
DK/GD/173/04
SN/GX/307/04
CK/HuB/489/04
CK/HuB/327/04
CK/HeN/16/04
CK/HeN/13/04
CK/HeN/12/04
SW/HeN/wy/04
CK/HeN/01/04
ML/GX/wt/04
DK/China/E319-2/03
DK/GD/23/04
CK/GD/178/04
DK/AH/56/05
SH/1/06
China/GD01/06
ML/hd/S/05
CK/HN/1793/07
DK/HN/3340/06
DK/HN/3315/06
PF/HK/2142/08
CK/FJ/1/07
DK/HN/29/06
DK/HN/11/07
DK/YN/47/06
GS/YN/4371/06
GS/YN/3798/06
R3
AV/HK/2372/07
AV/HK/1993/07
AV/HK/0828/07
AV/HK/0719/07
94/91/0.99
SE/TB/13/06
DK/YN/5310/06
CK/TB/6/08
GS/HuB/65/05
CK/SC/81/05
DK/HN/689/06
DK/HuB/2911/07
DGWT/HN/3450/06
CK/HN/3157/06
DK/HuB/hm01/06
AH/1/05
DK/HuB/49/05
DK/AH/1/06
AH/2/05
CK/HuB/2856/07
DK/SD/009/08
DK/EC/909/09
CK/HN/1/09
JS/2/07
JS/1/07
DK/JX/80/05
WDK/HN/021/05
ML/hd/lk/05
CK/HN/21/05
Group 4
GX/1/05
CK/GZ/7/08
China/GD02/06
100/100/1.0
BCNH/HK/659/08
SN/SH/10/09
DK/EC/108/08
DK/HN/3/07
CK/HN/3/07
DK/HN/8/08
CK/HN/8/08
GCG/QH/1/09
GS/JS/k0403/10
CK/JS/k0402/10
LH/HK/8550/07
GH/HK/3088/07
GE/HK/807/08
MR/HK/1097/08
MR/HK/1897/08
GH/HK/1046/08
HuB/1/10
DK/ZJ/213/11
DK/ZJ/2244/11
DK/ZJ/2224/11
DK/ZJ/2242/11
DK/ZJ/2248/11
DK/ZJ/2243/11
Group 3

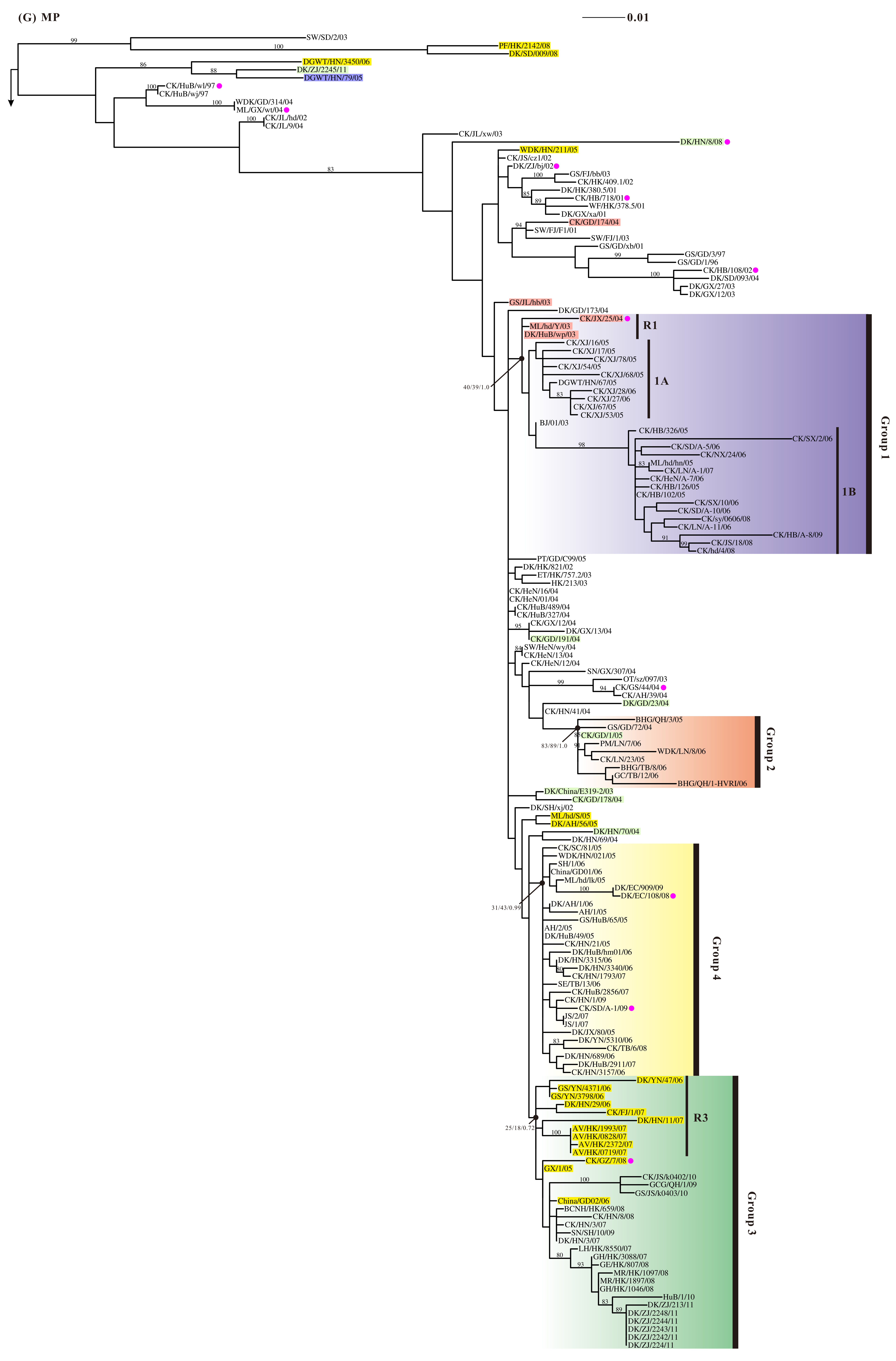

(G) MP
0.01
SW/SD/2/03
PF/HK/2142/08
DK/SD/009/08
DGWT/HN/3450/06
DK/ZJ/2245/11
DGWT/HN/79/05
CK/HuB/wl/97
CK/HuB/wj/97
WDK/GD/314/04
ML/GX/wt/04
CK/JL/hd/02
CK/JL/9/04
CK/JL/xw/03
DK/HN/8/08
WDK/HN/211/05
CK/JS/cz1/02
DK/ZJ/bj/02
GS/FJ/bb/03
CK/HK/409.1/02
DK/HK/380.5/01
CK/HB/718/01
WF/HK/378.5/01
DK/GX/xa/01
CK/GD/174/04
SW/FJ/F1/01
SW/FJ/1/03
GS/GD/xb/01
GS/GD/3/97
GS/GD/1/96
CK/HB/108/02
DK/SD/093/04
DK/GX/27/03
DK/GX/12/03
GS/JL/hb/03
DK/GD/173/04
CK/JX/25/04
ML/hd/Y/03
DK/HuB/wp/03
R1
CK/XJ/16/05
CK/XJ/17/05
CK/XJ/78/05
CK/XJ/54/05
CK/XJ/68/05
DGWT/HN/67/05
CK/XJ/28/06
CK/XJ/27/06
CK/XJ/67/05
CK/XJ/53/05
1A
40/39/1.0
BJ/01/03
CK/HB/326/05
CK/SX/2/06
CK/SD/A-5/06
CK/NX/24/06
ML/hd/hn/05
CK/LN/A-1/07
CK/HeN/A-7/06
CK/HB/126/05
CK/HB/102/05
CK/SX/10/06
CK/SD/A-10/06
CK/sy/0606/08
CK/LN/A-11/06
CK/HB/A-8/09
CK/JS/18/08
CK/hd/4/08
1B
Group 1
PT/GD/C99/05
DK/HK/821/02
ET/HK/757.2/03
HK/213/03
CK/HeN/16/04
CK/HeN/01/04
CK/HuB/489/04
CK/HuB/327/04
CK/GX/12/04
DK/GX/13/04
CK/GD/191/04
SW/HeN/wy/04
CK/HeN/13/04
CK/HeN/12/04
SN/GX/307/04
OT/sz/097/03
CK/GS/44/04
CK/AH/39/04
DK/GD/23/04
CK/HN/41/04
BHG/QH/3/05
GS/GD/72/04
CK/GD/1/05
83/89/1.0
PM/LN/7/06
WDK/LN/8/06
CK/LN/23/05
BHG/TB/8/06
GC/TB/12/06
BHG/QH/1-HVRI/06
Group 2
DK/China/E319-2/03
CK/GD/178/04
DK/SH/xj/02
ML/hd/S/05
DK/AH/56/05
DK/HN/70/04
DK/HN/69/04
CK/SC/81/05
WDK/HN/021/05
SH/1/06
China/GD01/06
ML/hd/lk/05
DK/EC/909/09
DK/EC/108/08
31/43/0.99
DK/AH/1/06
AH/1/05
GS/HuB/65/05
AH/2/05
DK/HuB/49/05
CK/HN/21/05
DK/HuB/hm01/06
DK/HN/3315/06
DK/HN/3340/06
CK/HN/1793/07
SE/TB/13/06
CK/HuB/2856/07
CK/HN/1/09
CK/SD/A-1/09
JS/2/07
JS/1/07
DK/JX/80/05
DK/YN/5310/06
CK/TB/6/08
DK/HN/689/06
DK/HuB/2911/07
CK/HN/3157/06
Group 4
DK/YN/47/06
GS/YN/4371/06
GS/YN/3798/06
DK/HN/29/06
CK/FJ/1/07
DK/HN/11/07
25/18/0.72
AV/HK/1993/07
AV/HK/0828/07
AV/HK/2372/07
AV/HK/0719/07
R3
CK/GZ/7/08
GX/1/05
CK/JS/k0402/10
GCG/QH/1/09
GS/JS/k0403/10
China/GD02/06
BCNH/HK/659/08
CK/HN/8/08
CK/HN/3/07
SN/SH/10/09
DK/HN/3/07
LH/HK/8550/07
GH/HK/3088/07
GE/HK/807/08
MR/HK/1097/08
MR/HK/1897/08
GH/HK/1046/08
HuB/1/10
DK/ZJ/213/11
DK/ZJ/2248/11
DK/ZJ/2244/11
DK/ZJ/2243/11
DK/ZJ/2242/11
DK/ZJ/224/11
Group 3

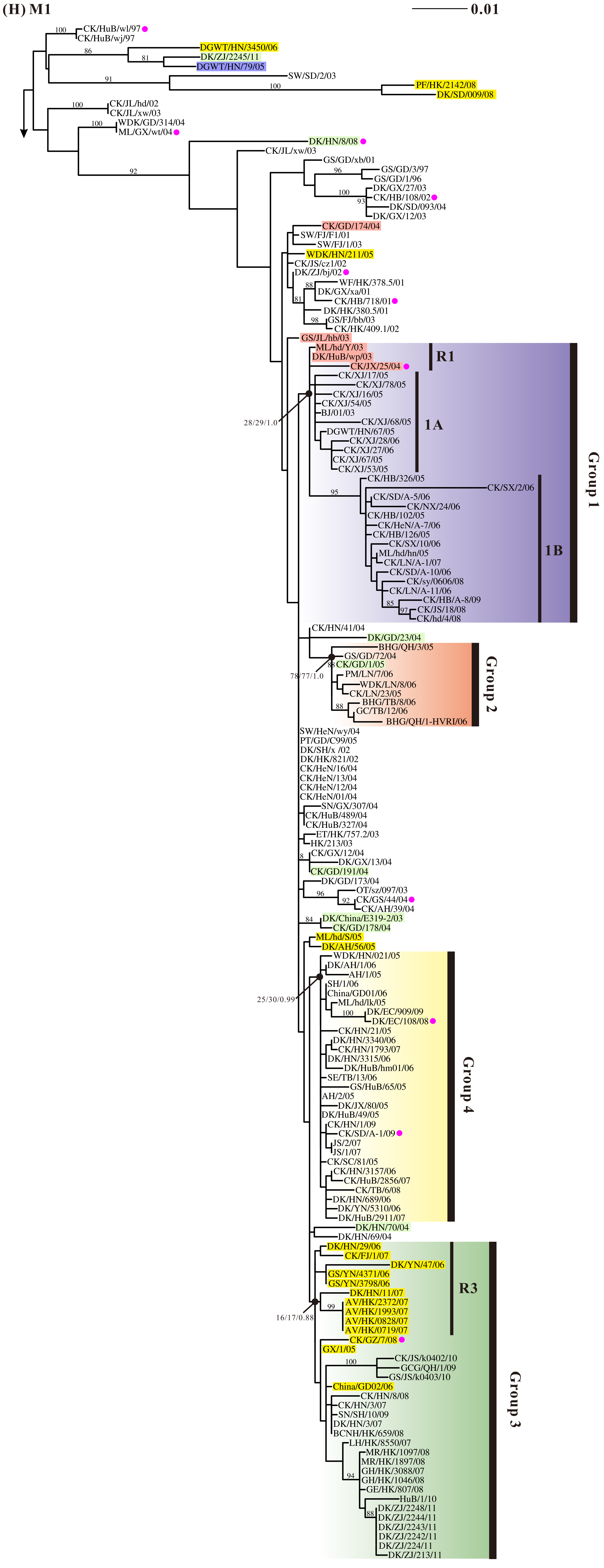

(H) M1
0.01
CK/HuB/wl/97
CK/HuB/wj/97
DGWT/HN/3450/06
DK/ZJ/2245/11
DGWT/HN/79/05
SW/SD/2/03
PF/HK/2142/08
DK/SD/009/08
CK/JL/hd/02
CK/JL/xw/03
WDK/GD/314/04
ML/GX/wt/04
DK/HN/8/08
CK/JL/xw/03
GS/GD/xb/01
GS/GD/3/97
GS/GD/1/96
DK/GX/27/03
CK/HB/108/02
DK/SD/093/04
DK/GX/12/03
CK/GD/174/04
SW/FJ/F1/01
SW/FJ/1/03
WDK/HN/211/05
CK/JS/cz1/02
DK/ZJ/bj/02
WF/HK/378.5/01
DK/GX/xa/01
CK/HB/718/01
DK/HK/380.5/01
GS/FJ/bb/03
CK/HK/409.1/02
GS/JL/hb/03
ML/hd/Y/03
DK/HuB/wp/03
CK/JX/25/04
R1
CK/XJ/17/05
CK/XJ/78/05
CK/XJ/16/05
CK/XJ/54/05
BJ/01/03
CK/XJ/68/05
DGWT/HN/67/05
CK/XJ/28/06
CK/XJ/27/06
CK/XJ/67/05
CK/XJ/53/05
1A
28/29/1.0
CK/HB/326/05
CK/SX/2/06
CK/SD/A-5/06
CK/NX/24/06
CK/HB/102/05
CK/HeN/A-7/06
CK/HB/126/05
CK/SX/10/06
ML/hd/hn/05
CK/LN/A-1/07
CK/SD/A-10/06
CK/sy/0606/08
CK/LN/A-11/06
CK/HB/A-8/09
CK/JS/18/08
CK/hd/4/08
1B
Group 1
CK/HN/41/04
DK/GD/23/04
BHG/QH/3/05
GS/GD/72/04
CK/GD/1/05
PM/LN/7/06
WDK/LN/8/06
CK/LN/23/05
BHG/TB/8/06
GC/TB/12/06
BHG/QH/1-HVRI/06
78/77/1.0
Group 2
SW/HeN/wy/04
PT/GD/C99/05
DK/SH/x /02
DK/HK/821/02
CK/HeN/16/04
CK/HeN/13/04
CK/HeN/12/04
CK/HeN/01/04
SN/GX/307/04
CK/HuB/489/04
CK/HuB/327/04
ET/HK/757.2/03
HK/213/03
CK/GX/12/04
DK/GX/13/04
CK/GD/191/04
DK/GD/173/04
OT/sz/097/03
CK/GS/44/04
CK/AH/39/04
DK/China/E319-2/03
CK/GD/178/04
ML/hd/S/05
DK/AH/56/05
WDK/HN/021/05
DK/AH/1/06
AH/1/05
SH/1/06
China/GD01/06
ML/hd/lk/05
DK/EC/909/09
DK/EC/108/08
25/30/0.99
CK/HN/21/05
DK/HN/3340/06
CK/HN/1793/07
DK/HN/3315/06
DK/HuB/hm01/06
SE/TB/13/06
GS/HuB/65/05
AH/2/05
DK/JX/80/05
DK/HuB/49/05
CK/HN/1/09
CK/SD/A-1/09
JS/2/07
JS/1/07
CK/SC/81/05
CK/HN/3157/06
CK/HuB/2856/07
CK/TB/6/08
DK/HN/689/06
DK/YN/5310/06
DK/HuB/2911/07
Group 4
DK/HN/70/04
DK/HN/69/04
DK/HN/29/06
CK/FJ/1/07
DK/YN/47/06
GS/YN/4371/06
GS/YN/3798/06
DK/HN/11/07
AV/HK/2372/07
AV/HK/1993/07
AV/HK/0828/07
AV/HK/0719/07
CK/GZ/7/08
R3
16/17/0.88
GX/1/05
CK/JS/k0402/10
GCG/QH/1/09
GS/JS/k0403/10
China/GD02/06
CK/HN/8/08
CK/HN/3/07
SN/SH/10/09
DK/HN/3/07
BCNH/HK/659/08
LH/HK/8550/07
MR/HK/1097/08
MR/HK/1897/08
GH/HK/3088/07
GH/HK/1046/08
GE/HK/807/08
HuB/1/10
DK/ZJ/2248/11
DK/ZJ/2244/11
DK/ZJ/2243/11
DK/ZJ/2242/11
DK/ZJ/224/11
DK/ZJ/213/11
Group 3

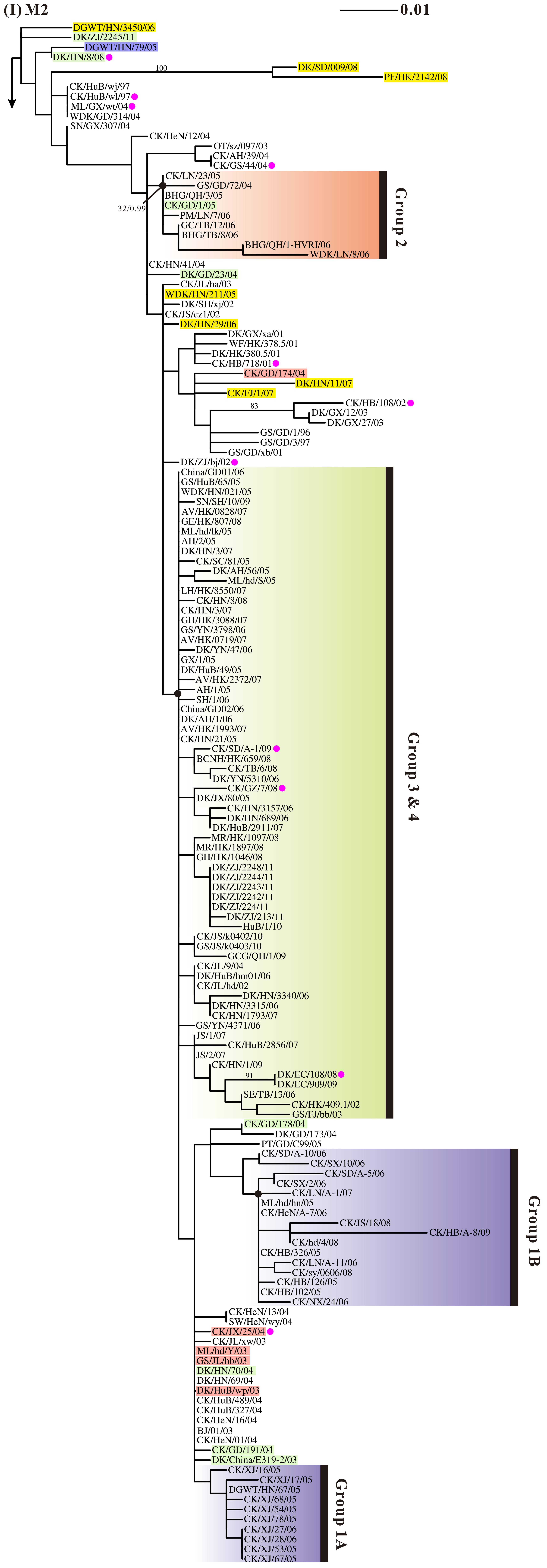

(I) M2
0.01
DGWT/HN/3450/06
DK/ZJ/2245/11
DGWT/HN/79/05
DK/HN/8/08
100
DK/SD/009/08
PF/HK/2142/08
CK/HuB/wj/97
CK/HuB/wl/97
ML/GX/wt/04
WDK/GD/314/04
SN/GX/307/04
CK/HeN/12/04
OT/sz/097/03
CK/AH/39/04
CK/GS/44/04
CK/LN/23/05
GS/GD/72/04
BHG/QH/3/05
CK/GD/1/05
32/0.99
PM/LN/7/06
GC/TB/12/06
BHG/TB/8/06
BHG/QH/1-HVRI/06
WDK/LN/8/06
Group 2
CK/HN/41/04
DK/GD/23/04
CK/JL/ha/03
WDK/HN/211/05
DK/SH/xj/02
CK/JS/cz1/02
DK/HN/29/06
DK/GX/xa/01
WF/HK/378.5/01
DK/HK/380.5/01
CK/HB/718/01
CK/GD/174/04
DK/HN/11/07
CK/FJ/1/07
83
CK/HB/108/02
DK/GX/12/03
DK/GX/27/03
GS/GD/1/96
GS/GD/3/97
GS/GD/xb/01
DK/ZJ/bj/02
China/GD01/06
GS/HuB/65/05
WDK/HN/021/05
SN/SH/10/09
AV/HK/0828/07
GE/HK/807/08
ML/hd/lk/05
AH/2/05
DK/HN/3/07
CK/SC/81/05
DK/AH/56/05
ML/hd/S/05
LH/HK/8550/07
CK/HN/8/08
CK/HN/3/07
GH/HK/3088/07
GS/YN/3798/06
AV/HK/0719/07
DK/YN/47/06
GX/1/05
DK/HuB/49/05
AV/HK/2372/07
AH/1/05
SH/1/06
China/GD02/06
DK/AH/1/06
AV/HK/1993/07
CK/HN/21/05
CK/SD/A-1/09
BCNH/HK/659/08
CK/TB/6/08
DK/YN/5310/06
CK/GZ/7/08
DK/JX/80/05
CK/HN/3157/06
DK/HN/689/06
DK/HuB/2911/07
MR/HK/1097/08
MR/HK/1897/08
GH/HK/1046/08
DK/ZJ/2248/11
DK/ZJ/2244/11
DK/ZJ/2243/11
DK/ZJ/2242/11
DK/ZJ/224/11
DK/ZJ/213/11
HuB/1/10
CK/JS/k0402/10
GS/JS/k0403/10
GCG/QH/1/09
CK/JL/9/04
DK/HuB/hm01/06
CK/JL/hd/02
DK/HN/3340/06
DK/HN/3315/06
CK/HN/1793/07
GS/YN/4371/06
JS/1/07
CK/HuB/2856/07
JS/2/07
CK/HN/1/09
91
DK/EC/108/08
DK/EC/909/09
SE/TB/13/06
CK/HK/409.1/02
GS/FJ/bb/03
Group 3 & 4
CK/GD/178/04
DK/GD/173/04
PT/GD/C99/05
CK/SD/A-10/06
CK/SX/10/06
CK/SD/A-5/06
CK/SX/2/06
CK/LN/A-1/07
ML/hd/hn/05
CK/HeN/A-7/06
CK/JS/18/08
CK/HB/A-8/09
CK/hd/4/08
CK/HB/326/05
CK/LN/A-11/06
CK/sy/0606/08
CK/HB/126/05
CK/HB/102/05
CK/NX/24/06
Group 1B
CK/HeN/13/04
SW/HeN/wy/04
CK/JX/25/04
CK/JL/xw/03
ML/hd/Y/03
GS/JL/hb/03
DK/HN/70/04
DK/HN/69/04
DK/HuB/wp/03
CK/HuB/489/04
CK/HuB/327/04
CK/HeN/16/04
BJ/01/03
CK/HeN/01/04
CK/GD/191/04
DK/China/E319-2/03
CK/XJ/16/05
CK/XJ/17/05
DGWT/HN/67/05
CK/XJ/68/05
CK/XJ/54/05
CK/XJ/78/05
CK/XJ/27/06
CK/XJ/28/06
CK/XJ/53/05
CK/XJ/67/05
Group 1A

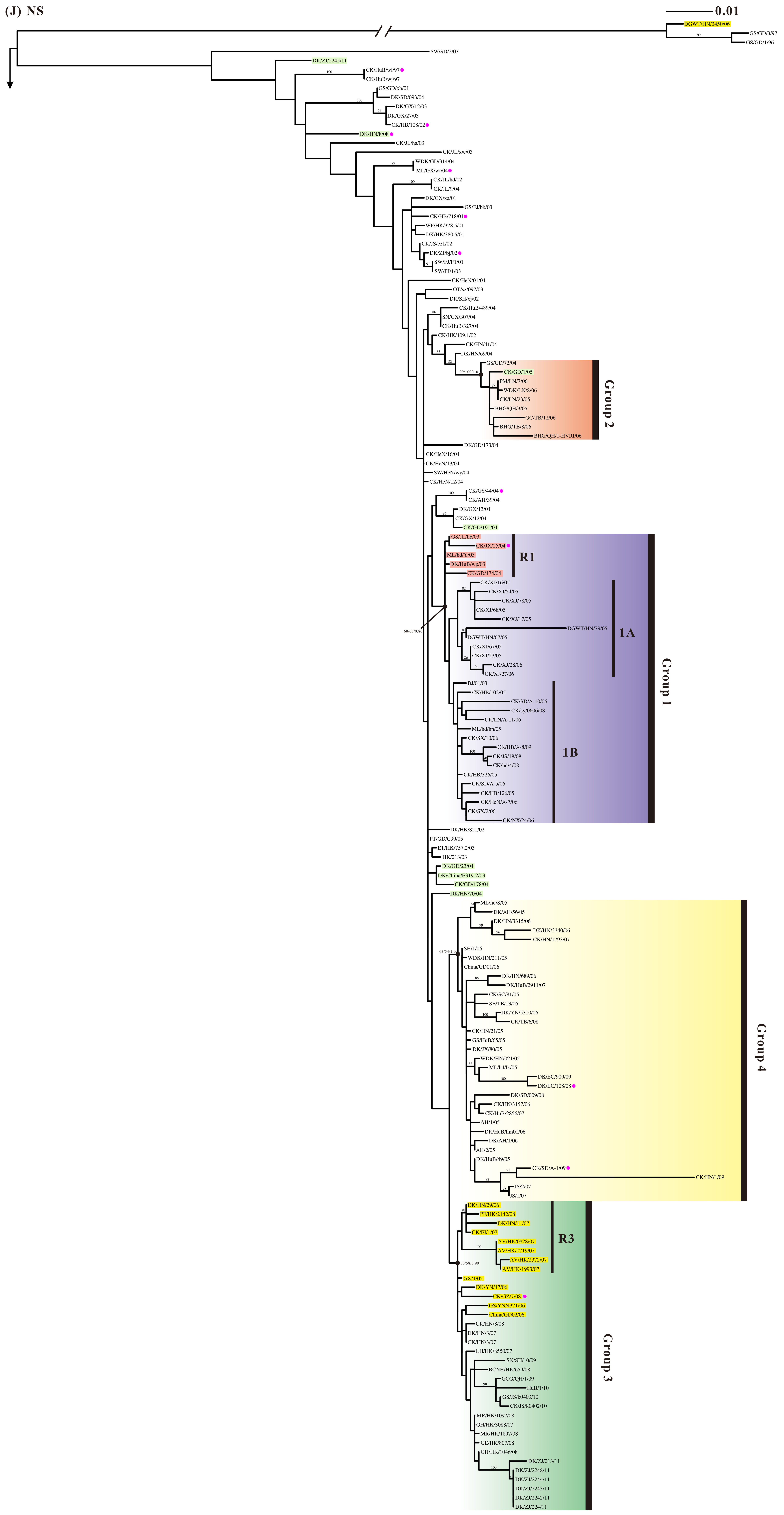
(J) NS
0.01
DGWT/HN/3450/06
GS/GD/3/97
GS/GD/1/96
SW/SD/2/03
DK/ZJ/2245/11
CK/HuB/wl/97
CK/HuB/wj/97
GS/GD/xb/01
DK/SD/093/04
DK/GX/12/03
DK/GX/27/03
CK/HB/108/02
DK/HN/8/08
CK/JL/ha/03
CK/JL/xw/03
WDK/GD/314/04
ML/GX/wt/04
CK/JL/hd/02
CK/JL/9/04
DK/GX/xa/01
GS/FJ/bb/03
CK/HB/718/01
WF/HK/378.5/01
DK/HK/380.5/01
CK/JS/cz1/02
DK/ZJ/bj/02
SW/FJ/F1/01
SW/FJ/1/03
CK/HeN/01/04
OT/sz/097/03
DK/SH/xj/02
CK/HuB/489/04
SN/GX/307/04
CK/HuB/327/04
CK/HK/409.1/02
CK/HN/41/04
DK/HN/69/04
GS/GD/72/04
CK/GD/1/05
PM/LN/7/06
WDK/LN/8/06
CK/LN/23/05
BHG/QH/3/05
GC/TB/12/06
BHG/TB/8/06
BHG/QH/1-HVRI/06
Group 2
DK/GD/173/04
CK/HeN/16/04
CK/HeN/13/04
SW/HeN/wy/04
CK/HeN/12/04
CK/GS/44/04
CK/AH/39/04
DK/GX/13/04
CK/GX/12/04
CK/GD/191/04
GS/JL/hb/03
CK/JX/25/04
ML/hd/Y/03
DK/HuB/wp/03
CK/GD/174/04
R1
CK/XJ/16/05
CK/XJ/54/05
CK/XJ/78/05
CK/XJ/68/05
CK/XJ/17/05
DGWT/HN/79/05
DGWT/HN/67/05
CK/XJ/67/05
CK/XJ/53/05
CK/XJ/28/06
CK/XJ/27/06
1A
BJ/01/03
CK/HB/102/05
CK/SD/A-10/06
CK/sy/0606/08
CK/LN/A-11/06
ML/hd/hn/05
CK/SX/10/06
CK/HB/A-8/09
CK/JS/18/08
CK/hd/4/08
CK/HB/326/05
CK/SD/A-5/06
CK/HB/126/05
CK/HeN/A-7/06
CK/SX/2/06
CK/NX/24/06
1B
Group 1
DK/HK/821/02
PT/GD/C99/05
ET/HK/757.2/03
HK/213/03
DK/GD/23/04
DK/China/E319-2/03
CK/GD/178/04
DK/HN/70/04
ML/hd/S/05
DK/AH/56/05
DK/HN/3315/06
DK/HN/3340/06
CK/HN/1793/07
SH/1/06
WDK/HN/211/05
China/GD01/06
DK/HN/689/06
DK/HuB/2911/07
CK/SC/81/05
SE/TB/13/06
DK/YN/5310/06
CK/TB/6/08
CK/HN/21/05
GS/HuB/65/05
DK/JX/80/05
WDK/HN/021/05
ML/hd/lk/05
DK/EC/909/09
DK/EC/108/08
DK/SD/009/08
CK/HN/3157/06
CK/HuB/2856/07
AH/1/05
DK/HuB/hm01/06
DK/AH/1/06
AH/2/05
DK/HuB/49/05
CK/SD/A-1/09
CK/HN/1/09
JS/2/07
JS/1/07
Group 4
DK/HN/29/06
PF/HK/2142/08
DK/HN/11/07
CK/FJ/1/07
AV/HK/0828/07
AV/HK/0719/07
AV/HK/2372/07
AV/HK/1993/07
R3
GX/1/05
DK/YN/47/06
CK/GZ/7/08
GS/YN/4371/06
China/GD02/06
CK/HN/8/08
DK/HN/3/07
CK/HN/3/07
LH/HK/8550/07
SN/SH/10/09
BCNH/HK/659/08
GCG/QH/1/09
HuB/1/10
GS/JS/k0403/10
CK/JS/k0402/10
MR/HK/1097/08
GH/HK/3088/07
MR/HK/1897/08
GE/HK/807/08
GH/HK/1046/08
DK/ZJ/213/11
DK/ZJ/2248/11
DK/ZJ/2244/11
DK/ZJ/2243/11
DK/ZJ/2242/11
DK/ZJ/224/11
Group 3

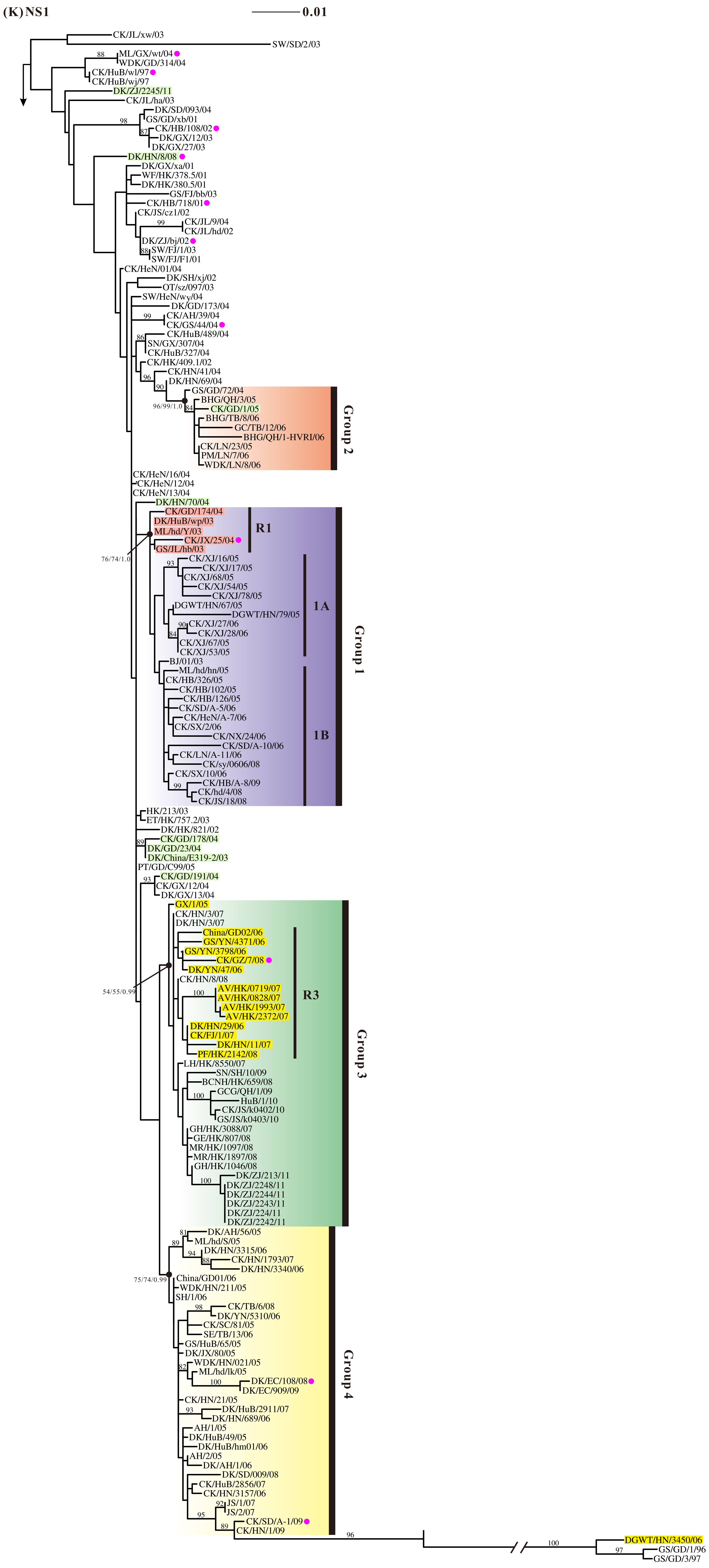
(K) NS1
0.01
CK/JL/xw/03
SW/SD/2/03
88
ML/GX/wt/04
WDK/GD/314/04
CK/HuB/wl/97
CK/HuB/wj/97
DK/ZJ/2245/11
CK/JL/ha/03
DK/SD/093/04
98
GS/GD/xb/01
87
CK/HB/108/02
DK/GX/12/03
DK/GX/27/03
DK/HN/8/08
DK/GX/xa/01
WF/HK/378.5/01
DK/HK/380.5/01
GS/FJ/bb/03
CK/HB/718/01
CK/JS/cz1/02
99
CK/JL/9/04
CK/JL/hd/02
DK/ZJ/bj/02
88
SW/FJ/1/03
SW/FJ/F1/01
CK/HeN/01/04
DK/SH/xj/02
OT/sz/097/03
SW/HeN/wy/04
DK/GD/173/04
99
CK/AH/39/04
CK/GS/44/04
86
CK/HuB/489/04
SN/GX/307/04
CK/HuB/327/04
CK/HK/409.1/02
96
CK/HN/41/04
90
DK/HN/69/04
GS/GD/72/04
96/99/1.0
BHG/QH/3/05
84
CK/GD/1/05
BHG/TB/8/06
GC/TB/12/06
BHG/QH/1-HVRI/06
CK/LN/23/05
PM/LN/7/06
WDK/LN/8/06
Group 2
CK/HeN/16/04
CK/HeN/12/04
CK/HeN/13/04
DK/HN/70/04
CK/GD/174/04
DK/HuB/wp/03
ML/hd/Y/03
CK/JX/25/04
GS/JL/hb/03
R1
76/74/1.0
93
CK/XJ/16/05
CK/XJ/17/05
CK/XJ/68/05
CK/XJ/54/05
CK/XJ/78/05
DGWT/HN/67/05
DGWT/HN/79/05
90
CK/XJ/27/06
84
CK/XJ/28/06
CK/XJ/67/05
CK/XJ/53/05
1A
Group 1
BJ/01/03
ML/hd/hn/05
CK/HB/326/05
CK/HB/102/05
CK/HB/126/05
CK/SD/A-5/06
CK/HeN/A-7/06
CK/SX/2/06
CK/NX/24/06
CK/SD/A-10/06
CK/LN/A-11/06
CK/sy/0606/08
CK/SX/10/06
99
CK/HB/A-8/09
CK/hd/4/08
CK/JS/18/08
1B
HK/213/03
ET/HK/757.2/03
DK/HK/821/02
89
CK/GD/178/04
DK/GD/23/04
DK/China/E319-2/03
PT/GD/C99/05
93
CK/GD/191/04
CK/GX/12/04
DK/GX/13/04
GX/1/05
CK/HN/3/07
DK/HN/3/07
China/GD02/06
GS/YN/4371/06
GS/YN/3798/06
CK/GZ/7/08
DK/YN/47/06
54/55/0.99
CK/HN/8/08
100
AV/HK/0719/07
AV/HK/0828/07
AV/HK/1993/07
AV/HK/2372/07
DK/HN/29/06
CK/FJ/1/07
DK/HN/11/07
PF/HK/2142/08
R3
Group 3
LH/HK/8550/07
SN/SH/10/09
BCNH/HK/659/08
100
GCG/QH/1/09
HuB/1/10
CK/JS/k0402/10
GS/JS/k0403/10
GH/HK/3088/07
GE/HK/807/08
MR/HK/1097/08
MR/HK/1897/08
GH/HK/1046/08
100
DK/ZJ/213/11
DK/ZJ/2248/11
DK/ZJ/2244/11
DK/ZJ/2243/11
DK/ZJ/224/11
DK/ZJ/2242/11
81
DK/AH/56/05
89
ML/hd/S/05
94
DK/HN/3315/06
88
CK/HN/1793/07
DK/HN/3340/06
75/74/0.99
China/GD01/06
WDK/HN/211/05
SH/1/06
98
CK/TB/6/08
DK/YN/5310/06
CK/SC/81/05
SE/TB/13/06
GS/HuB/65/05
DK/JX/80/05
82
WDK/HN/021/05
ML/hd/lk/05
100
DK/EC/108/08
DK/EC/909/09
Group 4
CK/HN/21/05
93
DK/HuB/2911/07
DK/HN/689/06
AH/1/05
DK/HuB/49/05
DK/HuB/hm01/06
AH/2/05
DK/AH/1/06
DK/SD/009/08
CK/HuB/2856/07
CK/HN/3157/06
92
JS/1/07
95
JS/2/07
89
CK/SD/A-1/09
CK/HN/1/09
96
100
DGWT/HN/3450/06
97
GS/GD/1/96
GS/GD/3/97

**(L) NS2**

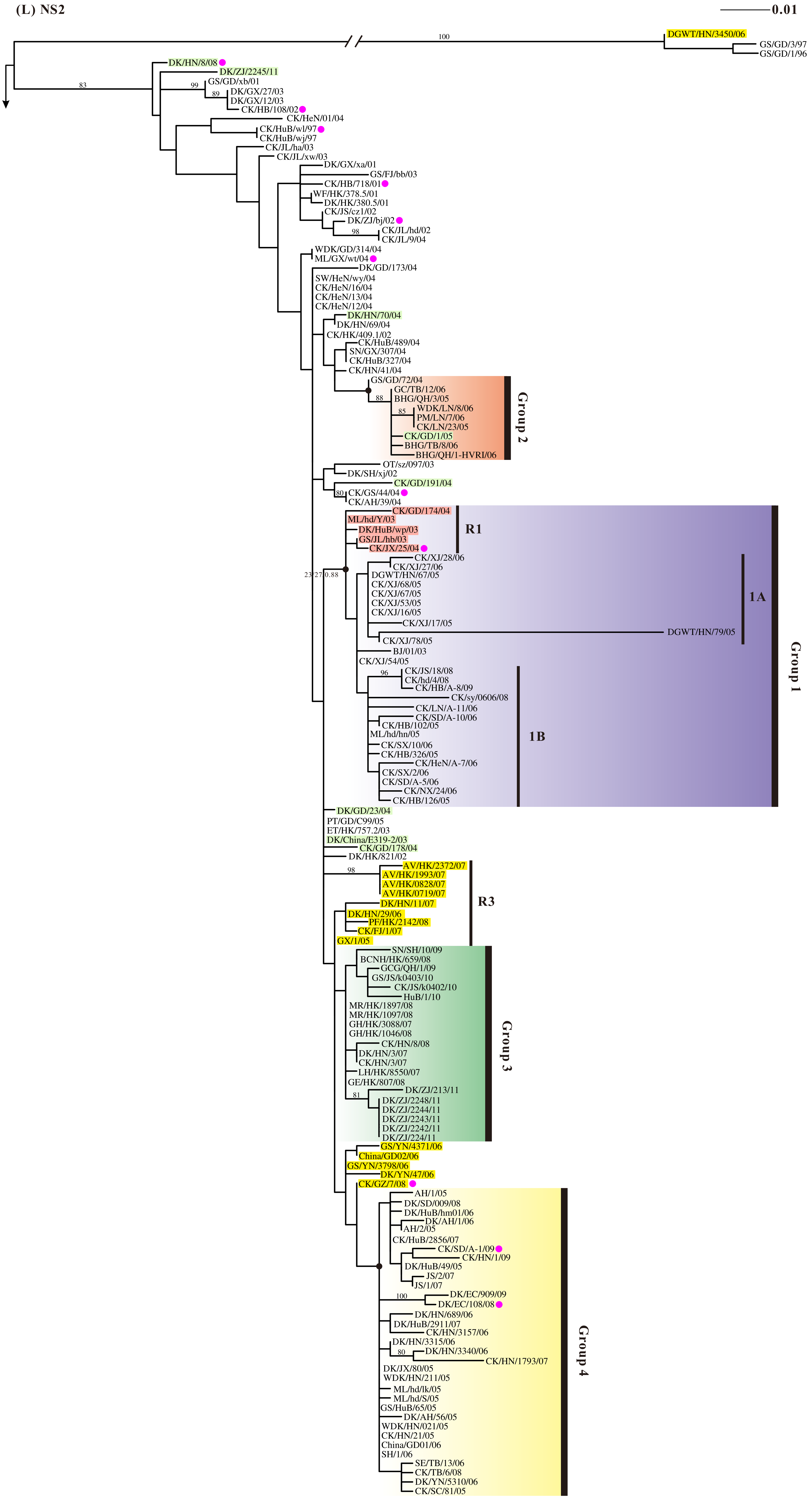
0.01
100
DGWT/HN/3450/06
GS/GD/3/97
GS/GD/1/96
83
DK/HN/8/08
DK/ZJ/2245/11
99
GS/GD/xb/01
89
DK/GX/27/03
DK/GX/12/03
CK/HB/108/02
CK/HeN/01/04
CK/HuB/wl/97
CK/HuB/wj/97
CK/JL/ha/03
CK/JL/xw/03
DK/GX/xa/01
GS/FJ/bb/03
CK/HB/718/01
WF/HK/378.5/01
DK/HK/380.5/01
CK/JS/cz1/02
DK/ZJ/bj/02
98
CK/JL/hd/02
CK/JL/9/04
WDK/GD/314/04
ML/GX/wt/04
DK/GD/173/04
SW/HeN/wy/04
CK/HeN/16/04
CK/HeN/13/04
CK/HeN/12/04
DK/HN/70/04
DK/HN/69/04
CK/HK/409.1/02
CK/HuB/489/04
SN/GX/307/04
CK/HuB/327/04
CK/HN/41/04
GS/GD/72/04
GC/TB/12/06
88
BHG/QH/3/05
85
WDK/LN/8/06
PM/LN/7/06
CK/LN/23/05
CK/GD/1/05
BHG/TB/8/06
BHG/QH/1-HVRI/06
Group 2
OT/sz/097/03
DK/SH/xj/02
CK/GD/191/04
80
CK/GS/44/04
CK/AH/39/04
CK/GD/174/04
ML/hd/Y/03
DK/HuB/wp/03
GS/JL/hb/03
CK/JX/25/04
R1
23/27/0.88
CK/XJ/28/06
CK/XJ/27/06
DGWT/HN/67/05
CK/XJ/68/05
CK/XJ/67/05
CK/XJ/53/05
CK/XJ/16/05
CK/XJ/17/05
DGWT/HN/79/05
CK/XJ/78/05
1A
BJ/01/03
CK/XJ/54/05
96
CK/JS/18/08
CK/hd/4/08
CK/HB/A-8/09
CK/sy/0606/08
CK/LN/A-11/06
CK/SD/A-10/06
CK/HB/102/05
ML/hd/hn/05
CK/SX/10/06
CK/HB/326/05
CK/HeN/A-7/06
CK/SX/2/06
CK/SD/A-5/06
CK/NX/24/06
CK/HB/126/05
1B
Group 1
DK/GD/23/04
PT/GD/C99/05
ET/HK/757.2/03
DK/China/E319-2/03
CK/GD/178/04
DK/HK/821/02
98
AV/HK/2372/07
AV/HK/1993/07
AV/HK/0828/07
AV/HK/0719/07
DK/HN/11/07
DK/HN/29/06
PF/HK/2142/08
CK/FJ/1/07
GX/1/05
R3
SN/SH/10/09
BCNH/HK/659/08
GCG/QH/1/09
GS/JS/k0403/10
CK/JS/k0402/10
HuB/1/10
MR/HK/1897/08
MR/HK/1097/08
GH/HK/3088/07
GH/HK/1046/08
CK/HN/8/08
DK/HN/3/07
CK/HN/3/07
LH/HK/8550/07
GE/HK/807/08
DK/ZJ/213/11
81
DK/ZJ/2248/11
DK/ZJ/2244/11
DK/ZJ/2243/11
DK/ZJ/2242/11
DK/ZJ/224/11
Group 3
GS/YN/4371/06
China/GD02/06
GS/YN/3798/06
DK/YN/47/06
CK/GZ/7/08
AH/1/05
DK/SD/009/08
DK/HuB/hm01/06
DK/AH/1/06
AH/2/05
CK/HuB/2856/07
CK/SD/A-1/09
CK/HN/1/09
DK/HuB/49/05
JS/2/07
JS/1/07
100
DK/EC/909/09
DK/EC/108/08
DK/HN/689/06
DK/HuB/2911/07
CK/HN/3157/06
DK/HN/3315/06
80
DK/HN/3340/06
CK/HN/1793/07
DK/JX/80/05
WDK/HN/211/05
ML/hd/lk/05
ML/hd/S/05
GS/HuB/65/05
DK/AH/56/05
WDK/HN/021/05
CK/HN/21/05
China/GD01/06
SH/1/06
SE/TB/13/06
CK/TB/6/08
DK/YN/5310/06
CK/SC/81/05
Group 4
