# Supplementary material for: Genetic Dynamic Analysis of the Influenza A H5N1 NS1 Gene in China
Source: PLoS One. 2014 Jul 8;9(7):e101384. doi: 10.1371/journal.pone.0101384 (PMC4086889; doi:10.1371/journal.pone.0101384)

Figure S2 The structure features of H5N1 non-structural protein NS1. (A) Phylogenetic analysis of the NS1 gene based on 462 nucleotide sequences of HPAI H5N1 isolates. (B) The NS1 amino acid sequence alignment for the four viruses (AH/2/05, CK/GD/1/05, HK/156/97 and GS/GD/1/96). The box indicates the previously identified important amino acid residues of NS1 protein. (C) Structural alignment of four H5N1 NS1 RBD (AH/2/05 (pink), CK/GD/1/05 (light green), HK/156/97 (salmon) and GS/GD/1/96 (sky blue)) with A/crow/Kyoto/T1/2004 (tan) H5N1 NS1 RBD. The amino acid residues at position 38 and 41 are labeled. (D) F3-binding pocket on NS1A (85-215). A hydrophobic pocket on the NS1A surface binds to the F3 Zn finger of F2F3. The NS1A amino acid residues presented by their molecular surface interact with the aromatic side chains of residues Y97, F98, and F102 of the F3 Zn finger of F2F3. (E) Schematic illustration of the binding domain structure of NS1 and two subunits of PI3K (p85β and p110). The same color coding is used throughout this article unless specified. Gray regions are linkers between domains. (F) Ribbon diagram of the NS1-p85β complex (Protein Data Bank code: 2V1Y for p85α iSH2 and 2GX9 for NS1) (G) Ribbon diagram of the NS1- p85β-p110 complex (Protein Data Bank code:2RD0).

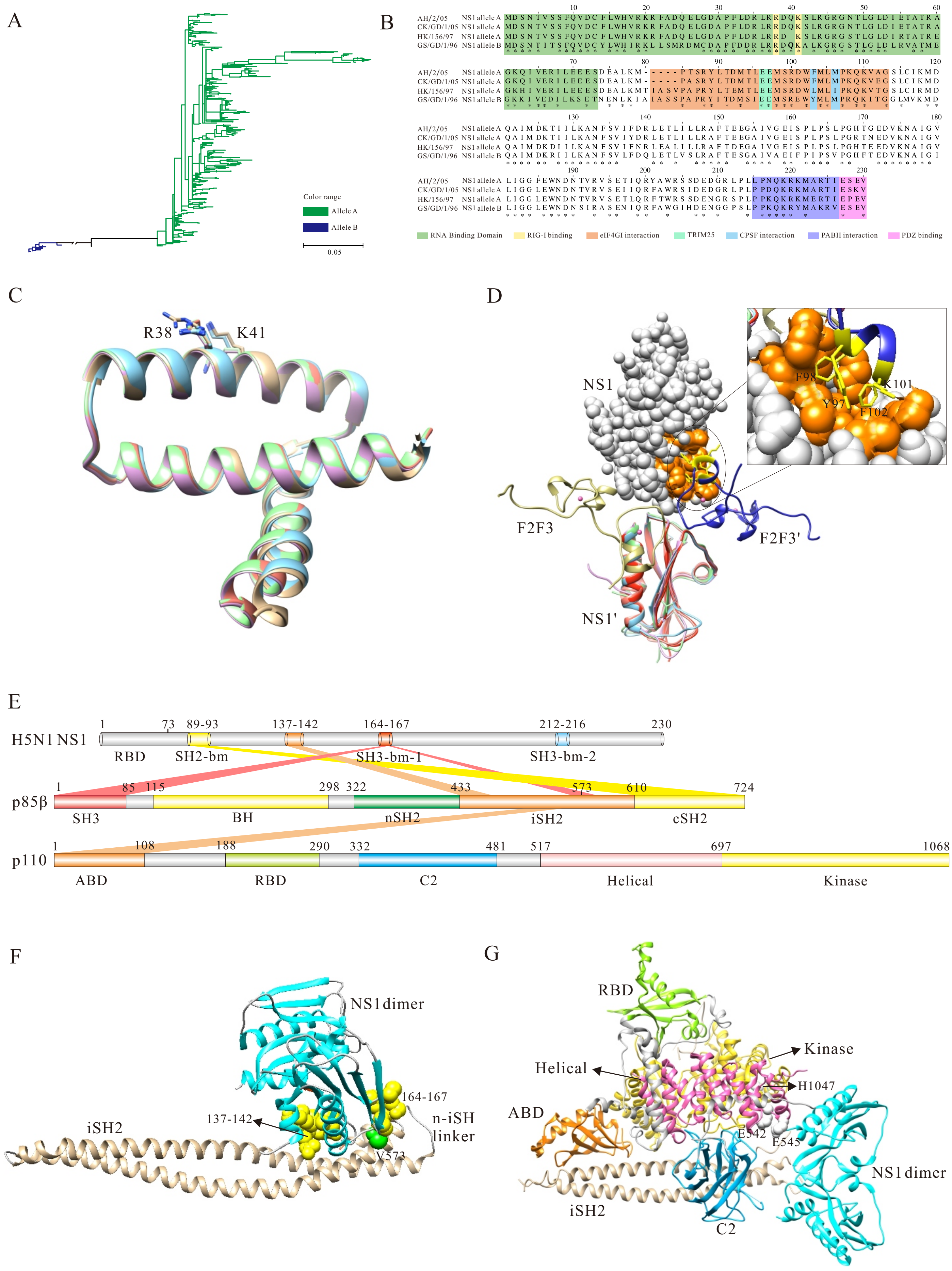

Supplement: Figure S2 — The structure features of H5N1 non-structural protein NS1. (A) Phylogenetic analysis of the NS1 gene based on 462 nucleotide sequences of HPAI H5N1 isolates. (B) The NS1 amino acid sequence alignment for the four viruses (AH/2/05, CK/GD/1/05, HK/156/97 and GS/GD/1/96). The box indicates the previously identified important amino acid residues of NS1 protein. (C) Structural alignment of four H5N1 NS1 RBD (AH/2/05 (pink), CK/GD/1/05 (light green), HK/156/97 (salmon) and GS/GD/1/96 (sky blue)) with A/crow/Kyoto/T1/2004 (tan) H5N1 NS1 RBD. The amino acid residues at position 38 and 41 are labeled. (D) F3-binding pocket on NS1A (85-215). A hydrophobic pocket on the NS1A surface binds to the F3 Zn finger of F2F3. The NS1A amino acid residues presented by their molecular surface interact with the aromatic side chains of residues Y97, F98, and F102 of the F3 Zn finger of F2F3. (E) Schematic illustration of the binding domain structure of NS1 and two subunits of PI3K (p85β and p110). The same color coding is used throughout this article unless specified. Gray regions are linkers between domains. (F) Ribbon diagram of the NS1-p85β complex (Protein Data Bank code: 2V1Y for p85α iSH2 and 2GX9 for NS1) (G) Ribbon diagram of the NS1- p85β-p110 complex (Protein Data Bank code: 2RD0). (PDF) [file pone.0101384.s002.pdf]
