# Supplementary material for: Genetic Dynamic Analysis of the Influenza A H5N1 NS1 Gene in China
Source: PLoS One. 2014 Jul 8;9(7):e101384. doi: 10.1371/journal.pone.0101384 (PMC4086889; doi:10.1371/journal.pone.0101384)
Supplement: Table S1 — H5N1 influenza viruses used in this study and their GenBank accession numbers. (DOC) [file pone.0101384.s004.doc]

| **Table S1 H5N1 influenza viruses used in this study and their GenBank accession numbers.** | | | | | | | | | | | | | |
| --- | --- | --- | --- | --- | --- | --- | --- | --- | --- | --- | --- | --- | --- |
| **Strain name** | **Abbreviation** | **GenBank access numbers** | | | | | | | | | | | |
|  |  | **PB2** | **PB1** | **PA** | **HA** | **NP** | **NA** | **MP** | **M1** | **M2** | **NS** | **NS1** | **NS2** |
| A/Anhui/1/2005 | AH/1/05 | ADG59510 | ADG59466 | ADG59414 | ADG59080 | ADG59278 | ADG59213 | HM172159 | ADG59177 | ADG59178 | HM172266 | ADG59295 | ADG59296 |
| A/Anhui/2/2005 | AH/2/05 | ADG59523 | ADG59431 | ADG59382 | ADG59048 | ADG59237 | ADG59189 | HM172120 | ADG59099 | ADG59100 | HM172264 | ADG59291 | ADG59292 |
| A/Beijing/01/2003 | BJ/01/03 | ABQ58976 | ABQ58977 | ABQ58978 | ABQ58979 | ABQ58980 | ABQ58981 | EF587280 | ABQ58982 | ABQ58983 | EF587281 | ABQ58984 | ABQ58985 |
| A/China/GD01/2006 | China/GD01/06 | ABI16501 | ABI16502 | ABI16503 | ABI16504 | ABI16505 | ABI16506 | DQ835316 | ABI16508 | ABI16507 | DQ835317 | ABI16510 | ABI16509 |
| A/China/GD02/2006 | China/GD02/06 | ABX57880 | ABX57879 | ABX57878 | ABX57871 | ABX57877 | ABX57872 | EU263984 | ABX57875 | ABX57876 | EU263983 | ABX57873 | ABX57874 |
| A/Duck/Hong Kong/380.5/2001 | DK/HK/380.5/01 | ACZ36505 | ACZ36550 | ACZ36595 | AAL75847 | ACZ36716 | AAL75848 | AY075035 | AAL75849 | AAL75850 | AY075036 | AAL75851 | AAL75852 |
| A/Guangxi/1/2005 | GX/1/05 | ADG59485 | ADG59441 | ADG59387 | ADG59086 | ADG59281 | ADG59235 | HM172149 | ADG59157 | ADG59158 | HM172298 | ADG59359 | ADG59360 |
| A/Hong Kong/213/2003 | HK/213/03 | BAE07198 | BAE07199 | BAE07200 | BAE07201 | BAE07202 | BAE07203 | AB212057 | BAE07204 |  | AB212059 | BAE07206 |  |
| A/Hubei/1/2010 | HuB/1/10 | AEO89177 | AEO89178 | AEO89180 | AEO89181 | AEO89182 | AEO89183 | CY098761 | AEO89184 | AEO89185 | CY098762 | AEO89186 | AEO89187 |
| A/Jiangsu/1/2007 | JS/1/07 | ACB87582 | ACB87581 | ACB87580 | ACB87573 | ACB87577 | ACB87576 | EU434687 | ACB87574 | ACB87575 | EU434690 | ACB87578 | ACB87579 |
| A/Jiangsu/2/2007 | JS/2/07 | ACB87572 | ACB87571 | ACB87570 | ACB87563 | ACB87567 | ACB87566 | EU434695 | ACB87564 | ACB87565 | EU434698 | ACB87568 | ACB87569 |
| A/Shanghai/1/2006 | SH/1/06 | BAH24016 | BAH24017 | BAH24019 | BAH10637 | BAH24020 | BAH24021 | AB462298 | BAH24023 | BAH24022 | AB462299 | BAH24025 | BAH24024 |
| A/avian/Hong Kong/0719/2007 | AV/HK/0719/07 | ACY79780 | ACY79893 | ACY80006 | ACY80658 | ACY80334 | ACY79679 | GU050318 | ACY80499 | ACY80500 | GU050321 | ACY80179 | ACY80180 |
| A/avian/Hong Kong/0828/2007 | AV/HK/0828/07 | ACY79781 | ACY79894 | ACY80007 | ACY80659 | ACY80335 | ACY79680 | GU050326 | ACY80501 | ACY80502 | GU050329 | ACY80181 | ACY80182 |
| A/avian/Hong Kong/1993/2007 | AV/HK/1993/07 | ACY79783 | ACY79896 | ACY80009 | ACY80661 | ACY80337 | ACY79682 | GU050342 | ACY80505 | ACY80506 | GU050345 | ACY80185 | ACY80186 |
| A/avian/Hong Kong/2372/2007 | AV/HK/2372/07 | ACY79782 | ACY79895 | ACY80008 | ACY80660 | ACY80336 | ACY79681 | GU050334 | ACY80503 | ACY80504 | GU050337 | ACY80183 | ACY80184 |
| A/bar-headed goose/Qinghai/1-HVRI/2006 | BHG/QH/1-HVRI/06 | ADG59487 | ADG59437 | ADG59404 | ADG59061 | ADG59242 | ADG59207 | HM172132 | ADG59123 | ADG59124 | HM172284 | ADG59331 | ADG59332 |
| A/bar-headed goose/Qinghai/3/2005 | BHG/QH/3/05 | ADG59561 | ADG59557 | ADG59554 | ADG59526 | ADG59541 | ADG59538 | HM172457 | ADG59529 | ADG59530 | HM172470 | ADG59547 | ADG59548 |
| A/bar-headed goose/Tibet/8/2006 | BHG/TB/8/06 | ADG59491 | ADG59444 | ADG59390 | ADG59063 | ADG59273 | ADG59209 | HM172128 | ADG59115 | ADG59116 | HM172277 | ADG59317 | ADG59318 |
| A/black-crowned night hero/Hong Kong/659/2008 | BCNH/HK/659/08 | ACJ26315 | ACJ26316 | ACJ26318 | ACJ26319 | ACJ26320 | ACJ26321 | CY036232 | ACJ26322 | ACJ26323 | CY036233 | ACJ26324 | ACJ26325 |
| A/chicken/Anhui/39/2004 | CK/AH/39/04 | ADG59502 | ADG59462 | ADG59392 | ADG59071 | ADG59261 | ADG59204 | HM172123 | ADG59105 | ADG59106 | HM172287 | ADG59337 | ADG59338 |
| A/chicken/Fujian/1/2007 | CK/FJ/1/07 | ADG59493 | ADG59445 | ADG59407 | ADG59072 | ADG59247 | ADG59193 | HM172122 | ADG59103 | ADG59104 | HM172272 | ADG59307 | ADG59308 |
| A/chicken/Gansu/44/2004 | CK/GS/44/04 | ADG59478 | ADG59460 | ADG59385 | ADG59051 | ADG59252 | ADG59211 | HM172124 | ADG59107 | ADG59108 | HM172286 | ADG59335 | ADG59336 |
| A/chicken/Guangdong/1/2005 | CK/GD/1/05 | ACG50703 | ACG50704 | ACG50697 | ACG50699 | ACG50698 | ACG50700 | EU874901 | ACG50701 | ACG50702 | EU874904 | ACG50706 | ACG50707 |
| A/chicken/Guangdong/174/04 | CK/GD/174/04 | AAT37560 | AAT37561 | AAT37562 | AAT37563 | AAT37564 | AAT37565 | AY609315 | AAT37566 | AAT37567 | AY609316 | AAT37568 | AAT37569 |
| A/chicken/Guangdong/178/04 | CK/GD/178/04 | AAW59395 | AAW59396 | AAW59397 | AAW59398 | AAW59399 | AAW59402 | AY737298 | AAW59400 | AAW59401 | AY737300 | AAW59404 | AAW59403 |
| A/chicken/Guangdong/191/04 | CK/GD/191/04 | AAW59387 | AAW59388 | AAW59389 | AAW59390 | AAW59391 | AAW59392 | AY737292 | AAW59393 | AAW59394 | AY737285 | AAW59385 | AAW59386 |
| A/chicken/Guangxi/12/2004 | CK/GX/12/04 | ABD14794 | ABD14799 | ABD14804 | ABD14809 | ABD14814 | ABD14819 | DQ366333 | ABD14824 |  | DQ366334 | ABD14789 |  |
| A/chicken/Guizhou/7/2008 | CK/GZ/7/08 | ADG59496 | ADG59442 | ADG59395 | ADG59055 | ADG59283 | ADG59212 | HM172157 | ADG59173 | ADG59174 | HM172279 | ADG59321 | ADG59322 |
| A/chicken/Hebei/102/2005 | CK/HB/102/05 | ABX10504 | ABX10503 | ABX10502 |  | ABX10499 | ABX10498 | EF175672 | ABO93192 | ABO93191 | EU243136 | ABX10500 | ABX10501 |
| A/chicken/Hebei/108/02 | CK/HB/108/02 | ABC74412 | ABC74413 | ABC74407 | ABC69150 | ABC74406 | ABC73059 | DQ351860 | ABC74397 | ABC74396 | DQ351861 | ABC74399 | ABC74398 |
| A/chicken/Hebei/126/2005 | CK/HB/126/05 | ABX10489 | ABX10490 | ABX10491 | ABX10496 | ABX10494 | ABX10495 | EF175673 | ABO93194 | ABO93193 | EU243143 | ABX10492 | ABX10493 |
| A/chicken/Hebei/326/2005 | CK/HB/326/05 | ABC74411 | ABC74415 | ABC74408 | ABC69148 | ABC74405 | ABC73061 | DQ351859 | ABC74395 | ABC74394 | DQ351862 | ABC74401 | ABC74400 |
| A/chicken/Hebei/718/2001 | CK/HB/718/01 | ABC74410 | ABC74414 | ABC74409 | ABC69149 | ABC74404 | ABC73060 | DQ351858 | ABC74393 | ABC74392 | DQ351863 | ABC74403 | ABC74402 |
| A/chicken/Hebei/A-8/2009 | CK/HB/A-8/09 | ADG59483 | ADG59438 | ADG59396 | ADG59057 | ADG59241 | ADG59221 | HM172137 | ADG59133 | ADG59134 | HM172271 | ADG59305 | ADG59306 |
| A/chicken/Henan/01/2004 | CK/HeN/01/04 | AAX53565 | AAX53559 | AAX53552 | AAX53504 | AAX53531 | AAX53525 | AY950237 | AAX53511 | AAX53512 | AY950258 | AAX53538 | AAX53539 |
| A/chicken/Henan/12/2004 | CK/HeN/12/04 | AAX53566 | AAX53560 | AAX53554 | AAX53506 | AAX53533 | AAX53527 | AY950239 | AAX53515 | AAX53516 | AY950260 | AAX53543 | AAX53542 |
| A/chicken/Henan/13/2004 | CK/HeN/13/04 | AAX53567 | AAX53561 | AAX53555 | AAX53507 | AAX53534 | AAX53528 | AY950240 | AAX53517 | AAX53518 | AY950261 | AAX53545 | AAX53544 |
| A/chicken/Henan/16/2004 | CK/HeN/16/04 | AAX53568 | AAX53562 | AAX53556 | AAX53508 | AAX53535 | AAX53529 | AY950241 | AAX53519 | AAX53520 | AY950262 | AAX53547 | AAX53546 |
| A/chicken/Henan/A-7/2006 | CK/HeN/A-7/06 | ADG59506 | ADG59446 | ADG59412 | ADG59056 | ADG59260 | ADG59225 | HM172127 | ADG59113 | ADG59114 | HM172291 | ADG59345 | ADG59346 |
| A/chicken/Hong Kong/409.1/2002 | CK/HK/409.1/02 | ACZ36507 | ACZ36552 | ACZ36597 | ACZ36884 | ACZ36718 | ACZ36760 | GU186673 | ACZ36815 | ACZ36816 | GU186676 | ACZ36655 | ACZ36656 |
| A/chicken/Huadong/4/2008 | CK/hd/4/08 | AFR53946 | AFR53947 | AFR53948 | ADR78657 | AFR53949 | ADR78658 | JX523360 | AFR53950 | AFR53951 | JX523361 | AFR53952 | AFR53953 |
| A/chicken/Hubei/2856/2007 | CK/HuB/2856/07 | ACN39435 | ACN39460 | ACN39482 | ACN39419 | ACN39498 | ACN39514 | FJ784883 | ACN39365 | ACN39366 | FJ784867 | ACN39397 | ACN39398 |
| A/chicken/Hubei/327/2004 | CK/HuB/327/04 | AAT90829 | AAT90830 | AAT90831 | AAT90832 | AAT90833 | AAT90834 | AY684709 | AAT90836 | AAT90835 | AY684710 | AAT90838 | AAT90837 |
| A/chicken/Hubei/489/2004 | CK/HuB/489/04 | AAV48551 |  | AAV48550 | AAV48546 | AAV48549 | AAV48545 | AY770077 | AAV48543 | AAV48544 | AY770080 | AAV48547 | AAV48548 |
| A/chicken/Hubei/wj/1997 | CK/HuB/wj/97 | ABI96728 | ABI96727 | ABI96724 | ABI96729 | ABI96721 | ABI96720 | DQ997119 | ABI96725 | ABI96726 | DQ997117 | ABI96722 | ABI96723 |
| A/chicken/Hubei/wl/1997 | CK/HuB/wl/97 | ABI96740 |  | ABI96746 | ABI96741 | ABI96743 | ABI96742 | DQ997138 | ABI96747 | ABI96748 | DQ997136 | ABI96744 | ABI96745 |
| A/chicken/Hunan/1/2009 | CK/HN/1/09 | ADG59497 | ADG59439 | ADG59405 | ADG59050 | ADG59279 | ADG59219 | HM172150 | ADG59159 | ADG59160 | HM172268 | ADG59299 | ADG59300 |
| A/chicken/Hunan/1793/2007 | CK/HN/1793/07 | ACN39431 | ACN39452 | ACN39478 | ACN39415 | ACN39494 | ACN39510 | FJ784879 | ACN39357 | ACN39358 | FJ784863 | ACN39389 | ACN39390 |
| A/chicken/Hunan/21/2005 | CK/HN/21/05 | ADG59499 | ADG59465 | ADG59425 | ADG59088 | ADG59257 | ADG59194 | HM172141 | ADG59141 | ADG59142 | HM172293 | ADG59349 | ADG59350 |
| A/chicken/Hunan/3/2007 | CK/HN/3/07 | ACZ05934 | ACZ05920 | ACZ05906 | ACZ05892 | ACZ05878 | ACZ05864 | GU182145 | ACZ05849 | ACZ05850 | GU182146 | ACZ05821 | ACZ05822 |
| A/chicken/Hunan/3157/2006 | CK/HN/3157/06 | ACN39438 | ACN39466 | ACN39485 | ACN39422 | ACN39501 | ACN39517 | FJ784886 | ACN39371 | ACN39372 | FJ784870 | ACN39403 | ACN39404 |
| A/chicken/Hunan/41/2004 | CK/HN/41/04 | ADG59500 | ADG59476 | ADG59391 | ADG59090 | ADG59248 | ADG59197 | HM172130 | ADG59119 | ADG59120 | HM172303 | ADG59369 | ADG59370 |
| A/chicken/Hunan/8/2008 | CK/HN/8/08 | ACZ05932 | ACZ05918 | ACZ05904 | ACZ05890 | ACZ05876 | ACZ05862 | GU182162 | ACZ05845 | ACZ05846 | GU182161 | ACZ05817 | ACZ05818 |
| A/chicken/Jiangsu/18/2008 | CK/JS/18/08 | ADG59489 | ADG59440 | ADG59424 | ADG59068 | ADG59258 | ADG59195 | HM172134 | ADG59127 | ADG59128 | HM172275 | ADG59313 | ADG59314 |
| A/chicken/Jiangsu/cz1/2002 | CK/JS/cz1/02 | ABI96766 | ABI96765 | ABI96772 | ABI96767 | ABI96769 | ABI96768 | DQ997179 | ABI96764 | ABI96763 | DQ997185 | ABI96771 | ABI96770 |
| A/chicken/Jiangsu/k0402/2010 | CK/JS/k0402/10 | AFC98316 | AFC98314 | AFC98312 | AFC98298 | AFC98306 | AFC98304 | JQ638675 | AFC98300 | AFC98301 | JQ638681 | AFC98308 | AFC98309 |
| A/chicken/Jiangxi/25/2004 | CK/JX/25/04 | ADG59563 | ADG59559 | ADG59555 | ADG59527 | ADG59543 | ADG59539 | HM172459 | ADG59533 | ADG59534 | HM172471 | ADG59549 | ADG59550 |
| A/chicken/Jilin/9/2004 | CK/JL/9/04 | AAT76157 | AAT76165 | AAT76164 | AAT76166 | AAT76161 | AAT76160 | AY653194 | AAT76159 | AAT76158 | AY653197 | AAT76163 | AAT76162 |
| A/chicken/Jilin/ha/2003 | CK/JL/ha/03 | ABI98913 | ABI98912 | ABI98918 | ABI98911 | ABI98915 | ABI98914 |  |  | ABI98910 | DQ997273 | ABI98916 | ABI98917 |
| A/chicken/Jilin/hd/2002 | CK/JL/hd/02 | ABI98926 | ABI98925 | ABI98924 | ABI98919 | ABI98921 | ABI98920 | DQ997290 | ABI98927 | ABI98928 | DQ997286 | ABI98922 | ABI98923 |
| A/chicken/Jilin/xw/2003 | CK/JL/xw/03 | ABI98937 | ABI98936 | ABI98943 | ABI98938 | ABI98940 | ABI98939 | DQ997552 | ABI98944 | ABI98945 | DQ997550 | ABI98941 | ABI98942 |
| A/chicken/Liaoning/23/2005 | CK/LN/23/05 | ADG59562 | ADG59558 | ADG59553 | ADG59525 | ADG59542 | ADG59537 | HM172458 | ADG59531 | ADG59532 | HM172469 | ADG59545 | ADG59546 |
| A/chicken/Liaoning/A-1/2007 | CK/LN/A-1/07 | ADG59505 | ADG59443 | ADG59410 | ADG59054 | ADG59284 | ADG59230 | HM172163 | ADG59185 | ADG59186 |  |  |  |
| A/chicken/Liaoning/A-11/2006 | CK/LN/A-11/06 | ADG59503 | ADG59434 | ADG59427 | ADG59053 | ADG59274 | ADG59220 | HM172148 | ADG59155 | ADG59156 | HM172308 | ADG59379 | ADG59380 |
| A/chicken/Ningxia/24/2006 | CK/NX/24/06 | ADG59515 | ADG59447 | ADG59408 | ADG59052 | ADG59265 | ADG59229 | HM172136 | ADG59131 | ADG59132 | HM172285 | ADG59333 | ADG59334 |
| A/chicken/Shandong/A-1/2009 | CK/SD/A-1/09 | ADG59486 | ADG59435 | ADG59401 | ADG59069 | ADG59276 | ADG59215 | HM172153 | ADG59165 | ADG59166 | HM172283 | ADG59329 | ADG59330 |
| A/chicken/Shandong/A-10/2006 | CK/SD/A-10/06 | ADG59490 | ADG59448 | ADG59399 | ADG59049 | ADG59268 | ADG59236 | HM172139 | ADG59137 | ADG59138 | HM172274 | ADG59311 | ADG59312 |
| A/chicken/Shandong/A-5/2006 | CK/SD/A-5/06 | ADG59516 | ADG59454 | ADG59388 | ADG59058 | ADG59259 | ADG59218 | HM172164 | ADG59187 | ADG59188 | HM172296 | ADG59355 | ADG59356 |
| A/chicken/Shanxi/10/2006 | CK/SX/10/06 | ADG59512 | ADG59433 | ADG59409 | ADG59089 | ADG59267 | ADG59196 | HM172135 | ADG59129 | ADG59130 | HM172297 | ADG59357 | ADG59358 |
| A/chicken/Shanxi/2/2006 | CK/SX/2/06 | ABK34762 | ABK34761 | ABK34763 | ABK34764 | ABK34765 | ABK34766 | DQ914817 | ABK34768 | ABK34767 | DQ914818 | ABK34770 | ABK34769 |
| A/chicken/Sheny/0606/2008 | CK/sy/0606/08 | AEX30607 | AEX30608 | AEX30610 | AEX30611 | AEX30612 | AEX30613 | JQ277228 | AEX30614 | AEX30615 | JQ277229 | AEX30616 | AEX30617 |
| A/chicken/Sichuan/81/2005 | CK/SC/81/05 | ADG59524 | ADG59429 | ADG59381 | ADG59045 | ADG59238 | ADG59191 | HM172118 | ADG59095 | ADG59096 | HM172261 | ADG59285 | ADG59286 |
| A/chicken/Tibet/6/2008 | CK/TB/6/08 | ADG59498 | ADG59452 | ADG59400 | ADG59062 | ADG59282 | ADG59226 | HM172162 | ADG59183 | ADG59184 | HM172282 | ADG59327 | ADG59328 |
| A/chicken/Xinjiang/16/2005 | CK/XJ/16/05 | ADG59492 | ADG59455 | ADG59423 | ADG59084 | ADG59243 | ADG59232 | HM172125 | ADG59109 | ADG59110 | HM172267 | ADG59297 | ADG59298 |
| A/chicken/Xinjiang/17/2005 | CK/XJ/17/05 | ADG59507 | ADG59471 | ADG59417 | ADG59078 | ADG59249 | ADG59200 | HM172161 | ADG59181 | ADG59182 | HM172276 | ADG59315 | ADG59316 |
| A/chicken/Xinjiang/27/2006 | CK/XJ/27/06 | ADG59520 | ADG59468 | ADG59416 | ADG59067 | ADG59264 | ADG59231 | HM172147 | ADG59153 | ADG59154 | HM172295 | ADG59353 | ADG59354 |
| A/chicken/Xinjiang/28/2006 | CK/XJ/28/06 | ADG59517 | ADG59458 | ADG59418 | ADG59066 | ADG59272 | ADG59217 | HM172144 | ADG59147 | ADG59148 | HM172289 | ADG59341 | ADG59342 |
| A/chicken/Xinjiang/53/2005 | CK/XJ/53/05 | ADG59514 | ADG59467 | ADG59422 | ADG59070 | ADG59275 | ADG59208 | HM172138 | ADG59135 | ADG59136 | HM172301 | ADG59365 | ADG59366 |
| A/chicken/Xinjiang/54/2005 | CK/XJ/54/05 | ADG59511 | ADG59472 | ADG59419 | ADG59087 | ADG59271 | ADG59228 | HM172142 | ADG59143 | ADG59144 | HM172305 | ADG59373 | ADG59374 |
| A/chicken/Xinjiang/67/2005 | CK/XJ/67/05 | ADG59508 | ADG59470 | ADG59421 | ADG59075 | ADG59263 | ADG59223 | HM172154 | ADG59167 | ADG59168 | HM172302 | ADG59367 | ADG59368 |
| A/chicken/Xinjiang/68/2005 | CK/XJ/68/05 | ADG59519 | ADG59464 | ADG59420 | ADG59082 | ADG59280 | ADG59216 | HM172145 | ADG59149 | ADG59150 | HM172299 | ADG59361 | ADG59362 |
| A/chicken/Xinjiang/78/2005 | CK/XJ/78/05 | ADG59518 | ADG59469 | ADG59415 | ADG59079 | ADG59270 | ADG59222 | HM172143 | ADG59145 | ADG59146 | HM172278 | ADG59319 | ADG59320 |
| A/domestic green-winged teal/Hunan/3450/2006 | DGWT/HN/3450/06 | AGH30417 | AGH30418 | AGH30420 | AGH30421 | AGH30422 | AGH30423 | KC690159 | AGH30424 | AGH30425 | KC690160 | AGH30426 | AGH30427 |
| A/domestic green-winged teal/Hunan/67/2005 | DGWT/HN/67/05 | ABZ91694 | ABZ91695 | ABZ91696 | ABZ91691 | ABZ91693 | ABZ91692 | EU430502 | ABZ91698 | ABZ91697 | EU430503 | ABZ91700 | ABZ91699 |
| A/domestic green-winged teal/Hunan/79/2005 | DGWT/HN/79/05 | ABZ91682 | ABZ91688 | ABZ91687 | ABZ91690 | ABZ91681 | ABZ91689 | EU430507 | ABZ91686 | ABZ91685 | EU430506 | ABZ91684 | ABZ91683 |
| A/duck/Anhui/1/06 | DK/AH/1/06 | ADG59481 | ADG59473 | ADG59428 | ADG59091 | ADG59262 | ADG59206 | HM172133 | ADG59125 | ADG59126 | HM172304 | ADG59371 | ADG59372 |
| A/duck/Anhui/56/2005 | DK/AH/56/05 | ADG59513 | ADG59474 | ADG59394 | ADG59083 | ADG59251 | ADG59199 | HM172152 | ADG59163 | ADG59164 | HM172270 | ADG59303 | ADG59304 |
| A/duck/China/E319-2/03 | DK/China/E319-2/03 | AAR99633 | AAR99632 | AAR99631 | AAR99628 | AAR99630 | AAR99629 | AY518361 | AAR99627 | AAR99626 | AY518360 | AAR99625 | AAR99624 |
| A/duck/Eastern China/108/2008 | DK/EC/108/08 | ADD10554 | ADD10555 | ADD10557 | ADD10558 | ADD10559 | ADD10560 | GU727672 | ADD10561 | ADD10562 | GU727673 | ADD10563 | ADD10564 |
| A/duck/Eastern China/909/2009 | DK/EC/909/09 | ADD10543 | ADD10545 | ADD10546 | ADD10547 | ADD10548 | ADD10549 | GU727680 | ADD10551 | ADD10550 | GU727681 | ADD10553 | ADD10552 |
| A/duck/Guangdong/173/04 | DK/GD/173/04 | AAW59405 | AAW59406 | AAW59407 | AAW59408 | AAW59409 | AAW59414 | AY737306 | AAW59410 | AAW59411 | AY737307 | AAW59412 | AAW59413 |
| A/duck/Guangdong/23/2004 | DK/GD/23/04 | ADG59504 | ADG59459 | ADG59393 | ADG59092 | ADG59254 | ADG59234 | HM172140 | ADG59139 | ADG59140 | HM172300 | ADG59363 | ADG59364 |
| A/duck/Guangxi/12/2003 | DK/GX/12/03 | ABW95934 | ABW95935 | ABW95937 | ABW95938 | ABW95939 | ABW95940 | EU263348 | ABW95941 | ABW95942 | EU263349 | ABW95943 | ABW95944 |
| A/duck/Guangxi/13/2004 | DK/GX/13/04 | ABD14795 | ABD14800 | ABD14805 | ABD14810 | ABD14815 | ABD14820 | DQ366341 | ABD14825 |  | DQ366342 | ABD14790 |  |
| A/duck/Guangxi/27/2003 | DK/GX/27/03 | ABW95945 | ABW95946 | ABW95948 | ABW95949 | ABW95950 | ABW95951 | EU263356 | ABW95952 | ABW95953 | EU263357 | ABW95954 | ABW95955 |
| A/duck/Guangxi/xa/2001 | DK/GX/xa/01 | ABJ09473 | ABJ09472 | ABJ09471 | ABJ09466 | ABJ09468 | ABJ09467 | DQ997520 | ABJ09474 | ABJ09475 | DQ997516 | ABJ09469 | ABJ09470 |
| A/duck/Hong Kong/821/2002 | DK/HK/821/02 | AAV97605 | AAV97597 | AAV97629 | AAV97601 | AAV97617 | AAV97613 | AY676045 | AAV97609 |  | AY676049 | AAV97621 | AAV97622 |
| A/duck/Hubei/2911/2007 | DK/HuB/2911/07 | ACN39436 | ACN39462 | ACN39483 | ACN39420 | ACN39499 | ACN39515 | FJ784884 | ACN39367 | ACN39368 | FJ784868 | ACN39399 | ACN39400 |
| A/duck/Hubei/49/2005 | DK/HuB/49/05 | ADG59521 | ADG59432 | ADG59383 | ADG59047 | ADG59240 | ADG59190 | HM172119 | ADG59097 | ADG59098 | HM172263 | ADG59289 | ADG59290 |
| A/duck/Hubei/Hangmei01/2006 | DK/HuB/hm01/06 | ACF16397 | ACF16398 | ACF16399 | ACF16400 | ACF16401 | ACF16402 | EU594352 | ACF16403 | ACF16404 | EU594353 | ACF16405 | ACF16406 |
| A/duck/Hubei/wp/2003 | DK/HuB/wp/03 | ABJ09483 | ABJ09482 | ABJ09481 | ABJ09476 | ABJ09478 | ABJ09477 | DQ997170 | ABJ09484 | ABJ09485 | DQ997166 | ABJ09479 | ABJ09480 |
| A/duck/Hunan/11/2007 | DK/HN/11/07 | ADG59479 | ADG59453 | ADG59403 | ADG59064 | ADG59269 | ADG59227 | HM172160 | ADG59179 | ADG59180 | HM172273 | ADG59309 | ADG59310 |
| A/duck/Hunan/29/2006 | DK/HN/29/06 | ADG59477 | ADG59451 | ADG59406 | ADG59073 | ADG59245 | ADG59201 | HM172129 | ADG59117 | ADG59118 | HM172294 | ADG59351 | ADG59352 |
| A/duck/Hunan/3/2007 | DK/HN/3/07 | ACZ05933 | ACZ05919 | ACZ05905 | ACZ05891 | ACZ05877 | ACZ05863 | GU182153 | ACZ05847 | ACZ05848 | GU182154 | ACZ05819 | ACZ05820 |
| A/duck/Hunan/3315/2006 | DK/HN/3315/06 | ACN39439 | ACN39468 | ACN39486 | ACN39423 | ACN39502 | ACN39518 | FJ784887 | ACN39373 | ACN39374 | FJ784871 | ACN39405 | ACN39406 |
| A/duck/Hunan/3340/2006 | DK/HN/3340/06 | ACN39440 | ACN39470 | ACN39487 | ACN39424 | ACN39503 | ACN39519 | FJ784888 | ACN39375 | ACN39376 | FJ784872 | ACN39407 | ACN39408 |
| A/duck/Hunan/689/2006 | DK/HN/689/06 | ACN39425 | ACN39441 | ACN39472 | ACN39409 | ACN39488 | ACN39504 | FJ784873 | ACN39345 | ACN39346 | FJ784857 | ACN39377 | ACN39378 |
| A/duck/Hunan/69/2004 | DK/HN/69/04 | ADG59494 | ADG59456 | ADG59397 | ADG59074 | ADG59253 | ADG59233 | HM172155 | ADG59169 | ADG59170 | HM172307 | ADG59377 | ADG59378 |
| A/duck/Hunan/70/2004 | DK/HN/70/04 | ADG59488 | ADG59461 | ADG59398 | ADG59081 | ADG59255 | ADG59202 | HM172158 | ADG59175 | ADG59176 | HM172306 | ADG59375 | ADG59376 |
| A/duck/Hunan/8/2008 | DK/HN/8/08 | ACZ05931 | ACZ05917 | ACZ05903 | ACZ05889 | ACZ05875 | ACZ05861 | GU182169 | ACZ05843 | ACZ05844 | GU182170 | ACZ05815 | ACZ05816 |
| A/duck/Jiangxi/80/2005 | DK/JX/80/05 | ADG59509 | ADG59463 | ADG59411 | ADG59076 | ADG59266 | ADG59224 | HM172151 | ADG59161 | ADG59162 | HM172290 | ADG59343 | ADG59344 |
| A/duck/Shandong/009/2008 | DK/SD/009/08 | ADC97011 | ADC97012 | ADC97014 | ADC97015 | ADC97016 | ADC97017 | GU596987 | ADC97018 | ADC97019 | GU596988 | ADC97020 | ADC97021 |
| A/duck/Shandong/093/2004 | DK/SD/093/04 | AAW72228 | AAW72229 | AAW72230 | AAW72226 | AAW72231 | AAW72227 | AY856865 | AAW72232 |  | AY856866 | AAW72233 |  |
| A/duck/Shanghai/xj/2002 | DK/SH/xj/02 | ABJ09487 | ABJ09486 | ABJ09493 | ABJ09488 | ABJ09490 | ABJ09489 | DQ997536 | ABJ09494 | ABJ09495 | DQ997534 | ABJ09491 | ABJ09492 |
| A/duck/Yunnan/47/2006 | DK/YN/47/06 | ADG59484 | ADG59457 | ADG59402 | ADG59085 | ADG59256 | ADG59210 | HM172126 | ADG59111 | ADG59112 | HM172288 | ADG59339 | ADG59340 |
| A/duck/Yunnan/5310/2006 | DK/YN/5310/06 | ACH85384 | ACH85385 | ACH85387 | ACH85388 | ACH85389 | ACH85390 | CY030892 | ACH85391 | ACH85392 | CY030893 | ACH85393 | ACH85394 |
| A/duck/Zhejiang/213/2011 | DK/ZJ/213/11 | AEO52369 | AEO52368 | AEO52367 | AEO52366 | AEO52365 | AEO52364 | JN646734 | AEO52362 | AEO52363 | JN646741 | AEO52360 | AEO52361 |
| A/duck/Zhejiang/224/2011 | DK/ZJ/224/11 | AEO52359 | AEO52358 | AEO52357 | AEO52356 | AEO52355 | AEO52354 | JN646735 | AEO52352 | AEO52353 | JN646742 | AEO52350 | AEO52351 |
| A/duck/Zhejiang/2242/2011 | DK/ZJ/2242/11 | AEO52349 | AEO52348 | AEO52347 | AEO52346 | AEO52345 | AEO52344 | JN646736 | AEO52342 | AEO52343 | JN646743 | AEO52340 | AEO52341 |
| A/duck/Zhejiang/2243/2011 | DK/ZJ/2243/11 | AEO52339 | AEO52338 | AEO52337 | AEO52336 | AEO52335 | AEO52334 | JN646737 | AEO52332 | AEO52333 | JN646744 | AEO52330 | AEO52331 |
| A/duck/Zhejiang/2244/2011 | DK/ZJ/2244/11 | AEO52329 | AEO52328 | AEO52327 | AEO52326 | AEO52325 | AEO52324 | JN646738 | AEO52322 | AEO52323 | JN646745 | AEO52320 | AEO52321 |
| A/duck/Zhejiang/2245/2011 | DK/ZJ/2245/11 | AEO52319 | AEO52318 | AEO52317 | AEO52316 | AEO52315 | AEO52314 | JN646739 | AEO52312 | AEO52313 | JN646746 | AEO52310 | AEO52311 |
| A/duck/Zhejiang/2248/2011 | DK/ZJ/2248/11 | AEO52309 | AEO52308 | AEO52307 | AEO52306 | AEO52305 | AEO52304 | JN646740 | AEO52302 | AEO52303 | JN646747 | AEO52300 | AEO52301 |
| A/duck/Zhejiang/bj/2002 | DK/ZJ/bj/02 | ABJ09505 | ABJ09504 | ABJ09503 | ABJ09498 | ABJ09500 | ABJ09499 | DQ997409 | ABJ09496 | ABJ09497 | DQ997413 | ABJ09501 | ABJ09502 |
| A/egret/Hong Kong/757.2/2003 | ET/HK/757.2/03 | AAV97606 | AAV97598 | AAV97630 | AAV97602 | AAV97618 | AAV97614 | AY676046 | AAV97610 |  | AY676050 | AAV97623 | AAV97624 |
| A/goose/Fujian/bb/2003 | GS/FJ/bb/03 | ABJ09510 | ABJ09509 | ABJ09506 | ABJ09511 | ABJ09513 | ABJ09512 | DQ997402 | ABJ09507 | ABJ09508 | DQ997408 | ABJ09514 | ABJ09515 |
| A/goose/Guangdong/1/1996 | GS/GD/1/96 | AAD51922 | AAD51923 | AAD51924 | AAD51927 | AAD51925 | AAD51926 | AF144306 | AAD51928 | AAD51929 | AF144307 | AAD51930 | AAD51931 |
| A/goose/Guangdong/3/1997 | GS/GD/3/97 | AAK72397 | AAK64188 | AAK55426 | AAK38298 | AAK60145 | AAK38299 | AF359560 | AAK26664 | AAK26663 | AY028445 | AAK38762 | AAK38763 |
| A/goose/Guangdong/72/2004 | GS/GD/72/04 | ADG59501 | ADG59449 | ADG59426 | ADG59065 | ADG59244 | ADG59205 | HM172156 | ADG59171 | ADG59172 | HM172280 | ADG59323 | ADG59324 |
| A/goose/Guangdong/xb/2001 | GS/GD/xb/01 | ABJ09525 | ABJ09524 | ABJ09523 | ABJ09518 | ABJ09520 | ABJ09519 | DQ997521 | ABJ09516 | ABJ09517 | DQ997525 | ABJ09521 | ABJ09522 |
| A/goose/Hubei/65/2005 | GS/HuB/65/05 | ADG59522 | ADG59430 | ADG59384 | ADG59046 | ADG59239 | ADG59192 | HM172117 | ADG59093 | ADG59094 | HM172262 | ADG59287 | ADG59288 |
| A/goose/Jiangsu/k0403/2010 | GS/JS/k0403/10 | AFC98317 | AFC98315 | AFC98313 | AFC98299 | AFC98307 | AFC98305 | JQ638676 | AFC98302 | AFC98303 | JQ638682 | AFC98310 | AFC98311 |
| A/goose/Jilin/hb/2003 | GS/JL/hb/03 | ABJ09535 | ABJ09534 | ABJ09533 | ABJ09528 | ABJ09530 | ABJ09529 | DQ997275 | ABJ09526 | ABJ09527 | DQ997279 | ABJ09531 | ABJ09532 |
| A/goose/Yunnan/3798/2006 | GS/YN/3798/06 | ACH85406 | ACH85407 | ACH85409 | ACH85410 | ACH85411 | ACH85412 | CY030908 | ACH85413 | ACH85414 |  | ACH85415 | ACH85416 |
| A/goose/Yunnan/4371/2006 | GS/YN/4371/06 | ACH85417 | ACH85418 | ACH85420 | ACH85421 | ACH85422 | ACH85423 | CY030916 | ACH85424 | ACH85425 | CY030917 | ACH85426 | ACH85427 |
| A/GC/Tibet/12/2006 | GC/TB/12/06 | ADG59480 |  | ADG59389 | ADG59059 | ADG59277 | ADG59203 | HM172121 | ADG59101 | ADG59102 | HM172292 | ADG59347 | ADG59348 |
| A/great crested-grebe/Qinghai/1/2009 | GCG/QH/1/09 | ADG44984 | ADG44985 | ADG44987 | ADG44988 | ADG44989 | ADG44990 | CY063321 | ADG44991 | ADG44992 | CY063322 | ADG44993 | ADG44994 |
| A/great egret/Hong Kong/807/2008 | GE/HK/807/08 | ACJ26326 | ACJ26327 | ACJ26329 | ACJ26330 | ACJ26331 | ACJ26332 | CY036240 | ACJ26333 | ACJ26334 | CY036241 | ACJ26335 | ACJ26336 |
| A/grey heron/Hong Kong/1046/2008 | GH/HK/1046/08 | ACJ26337 | ACJ26338 | ACJ26340 | ACJ26341 | ACJ26342 | ACJ26343 | CY036248 | ACJ26344 | ACJ26345 | CY036249 | ACJ26346 | ACJ26347 |
| A/grey heron/Hong Kong/3088/2007 | GH/HK/3088/07 | ACJ26293 | ACJ26294 | ACJ26296 | ACJ26297 | ACJ26298 | ACJ26299 | CY036216 | ACJ26300 | ACJ26301 | CY036217 | ACJ26302 | ACJ26303 |
| A/little heron/Hong Kong/8550/2007 | LH/HK/8550/07 | ACJ26271 | ACJ26272 | ACJ26274 | ACJ26275 | ACJ26276 | ACJ26277 | CY036200 | ACJ26278 | ACJ26279 | CY036201 | ACJ26280 | ACJ26281 |
| A/magpie robin/Hong Kong/1097/2008 | MR/HK/1097/08 | ACJ26348 | ACJ26349 | ACJ26351 | ACJ26352 | ACJ26353 | ACJ26354 | CY036256 | ACJ26355 | ACJ26356 | CY036257 | ACJ26357 | ACJ26358 |
| A/magpie robin/Hong Kong/1897/2008 | MR/HK/1897/08 | ACJ26359 | ACJ26360 | ACJ26362 | ACJ26363 | ACJ26364 | ACJ26365 | CY036264 | ACJ26366 | ACJ26367 | CY036265 | ACJ26368 | ACJ26369 |
| A/mallard/Guangxi/wt/2004 | ML/GX/wt/04 | ABJ09544 | ABJ09543 | ABJ09542 | ABJ09545 | ABJ09537 | ABJ09536 | DQ997214 | ABJ09540 | ABJ09541 | DQ997213 | ABJ09538 | ABJ09539 |
| A/mallard/Huadong/S/2005 | ML/hd/S/05 | ABW21644 | ABW21645 | ABW21646 | ABW21647 | ABW21648 | ABW21649 | EU195395 | ABW21651 | ABW21650 | EU195396 | ABW21653 | ABW21652 |
| A/mallard/Huadong/Y/2003 | ML/hd/Y/03 | ABW21674 | ABW21675 | ABW21676 | ABW21677 | ABW21678 | ABW21679 | EU195419 | ABW21681 | ABW21680 | EU195420 | ABW21683 | ABW21682 |
| A/mallard/Huadong/hn/2005 | ML/hd/hn/05 | ABW21664 | ABW21665 | ABW21666 | ABW21667 | ABW21668 | ABW21669 | EU195411 | ABW21671 | ABW21670 | EU195412 | ABW21673 | ABW21672 |
| A/mallard/Huadong/lk/2005 | ML/hd/lk/05 | ABW21654 | ABW21655 | ABW21656 | ABW21657 | ABW21658 | ABW21659 | EU195403 | ABW21661 | ABW21660 | EU195404 | ABW21663 | ABW21662 |
| A/ostrich/Suzhou/097/2003 | OT/sz/097/03 | AEB26723 | AEB26725 | AEB26720 | AEB26717 | AEB26724 | AEB26716 | JF732740 | AEB26718 | AEB26719 | JF732742 | AEB26721 | AEB26722 |
| A/parrot/Guangdong/C99/2005 | PT/GD/C99/05 | AFK73126 | AFK73127 | AFK73128 | AFK73124 | AFK73129 | AFK73125 | JX013491 | AFK73130 | AFK73131 | JX013492 | AFK73132 | AFK73133 |
| A/peregrine falcon/Hong Kong/2142/2008 | PF/HK/2142/08 | ACJ26370 | ACJ26371 | ACJ26373 | ACJ26374 | ACJ26375 | ACJ26376 | CY036272 | ACJ26377 | ACJ26378 | CY036273 | ACJ26379 | ACJ26380 |
| A/pied magpie/Liaoning/7/2006 | PM/LN/7/06 | ADG59564 | ADG59560 | ADG59556 | ADG59528 | ADG59544 | ADG59540 | HM172460 | ADG59535 | ADG59536 | HM172472 | ADG59551 | ADG59552 |
| A/shrike/Tibet/13/2006 | SE/TB/13/06 | ADG59482 | ADG59436 | ADG59413 | ADG59077 | ADG59246 | ADG59198 | HM172131 | ADG59121 | ADG59122 | HM172281 | ADG59325 | ADG59326 |
| A/swan/Guangxi/307/2004 | SN/GX/307/04 | AAX53570 | AAX53564 | AAX53558 | AAX53510 | AAX53537 | AAX53530 | AY950243 | AAX53523 | AAX53524 | AY950264 | AAX53551 | AAX53550 |
| A/swan/Shanghai/10/2009 | SN/SH/10/09 | AEG20976 | AEG20977 | AEG20979 | AEG20980 | AEG20981 | AEG20982 | JF975564 | AEG20983 | AEG20984 | JF975565 | AEG20985 | AEG20986 |
| A/swine/Fujian/1/2003 | SW/FJ/1/03 | AAV30835 | AAV30834 | AAV30833 | AAV30828 | AAV30830 | AAV30829 | AY747612 | AAV30831 |  | AY747613 | AAV30832 |  |
| A/swine/Fujian/F1/2001 | SW/FJ/F1/01 | AAV30843 | AAV30842 | AAV30841 | AAV30836 | AAV30838 | AAV30837 | AY747620 | AAV30839 |  | AY747621 | AAV30840 |  |
| A/swine/Henan/wy/2004 | SW/HeN/wy/04 | ABJ16472 | ABJ16471 | ABJ16478 | ABJ16473 | ABJ16475 | ABJ16474 | DQ997258 | ABJ16479 | ABJ16480 | DQ997256 | ABJ16476 | ABJ16477 |
| A/swine/Shandong/2/03 | SW/SD/2/03 | AAU05323 | AAU05322 | AAU05321 | AAT72505 | AAT72507 | AAT72506 | AY700217 | AAU05325 |  | AY700216 | AAU05324 |  |
| A/waterfowl/Hong Kong/378.5/2001 | WF/HK/378.5/01 | ACZ36504 | ACZ36549 | ACZ36594 | ACZ36882 | ACZ36715 | ACZ36758 | GU186693 | ACZ36811 | ACZ36812 | GU186696 | ACZ36651 | ACZ36652 |
| A/wild duck/Guangdong/314/2004 | WDK/GD/314/04 | AAX53569 | AAX53563 | AAX53557 | AAX53509 | AAX53536 |  | AY950242 | AAX53521 | AAX53522 | AY950263 | AAX53549 | AAX53548 |
| A/wild duck/Hunan/021/2005 | WDK/HN/021/05 | ABX83935 | ABX83936 | ABX83937 | ABX83938 | ABX83939 | ABX83940 | EU329180 | ABX83941 | ABX83942 | EU329181 | ABX83943 | ABX83944 |
| A/wild duck/Hunan/211/2005 | WDK/HN/211/05 | ABX83954 | ABX83953 | ABX83952 | ABX83951 | ABX83950 | ABX83949 | EU329183 | ABX83947 | ABX83948 | EU329182 | ABX83945 | ABX83946 |
| A/wild duck/Liaoning/8/2006 | WDK/LN/8/06 | ADG59495 | ADG59475 | ADG59386 | ADG59060 | ADG59250 | ADG59214 | HM172146 | ADG59151 | ADG59152 | HM172269 | ADG59301 | ADG59302 |
