# Supplementary material for: Genetic Dynamic Analysis of the Influenza A H5N1 NS1 Gene in China
Source: PLoS One. 2014 Jul 8;9(7):e101384. doi: 10.1371/journal.pone.0101384 (PMC4086889; doi:10.1371/journal.pone.0101384)
Supplement: Table S2 — Pairwise intergroup distance of HA gene. (DOC) [file pone.0101384.s005.doc]

| **Table S2 Pairwise intergroup distance of HA gene.** | | | | |
| --- | --- | --- | --- | --- |
|  | Group1 | Group2 | Group3 | Group4 |
| Group1 |  | [0.004]^a^ | [0.005] | [0.004] |
| Group2 | 0.056 |  | [0.004] | [0.003] |
| Group3 | 0.075 | 0.052 |  | [0.004] |
| Group4 | 0.059 | 0.037 | 0.049 |  |
| ^a^Standard error. | | | | |
