# Supplementary material for: Genetic Dynamic Analysis of the Influenza A H5N1 NS1 Gene in China
Source: PLoS One. 2014 Jul 8;9(7):e101384. doi: 10.1371/journal.pone.0101384 (PMC4086889; doi:10.1371/journal.pone.0101384)
Supplement: Table S3 — Sequence information and phylogenetic groupings of sequences used in this study. (DOC) [file pone.0101384.s006.doc]

| **Table S3 Sequence information and phylogenetic groupings of sequences used in this study.** | | | | | | | | | |
| --- | --- | --- | --- | --- | --- | --- | --- | --- | --- |
| Strain name | PB2 | PB1 | PA | HA | NP | NA | MP | NS | REF |
| GS/GD/1/96 | X | X | X | X | X | X | X | X |  |
| GS/GD/3/97 | X | X | X | X | X | X | X | X |  |
| CK/HuB/wj/97 | X | X | X | X | X | X | X | X |  |
| CK/HuB/wl/97* | X |  | X | X | X | X | X | X |  |
| SW/SD/2/03 | X | X | X | X | X | X | X | X |  |
| CK/JL/xw/03 | X | X | X | X | X | X | X | X |  |
| GS/GD/xb/01 | X | X | X | X | X | X | X | X |  |
| DK/SD/093/04 | X | X | X | X | X | X | X | X |  |
| OT/sz/097/03 | X | X | X | X | X | X | X | X |  |
| DK/GX/12/03 | X | X | X | X | X | X | X | X |  |
| DK/GX/27/03 | X | X | X | X | X | X | X | X |  |
| CK/HB/108/02* | X | X | X | X | X | X | X | X |  |
| CK/JL/ha/03 | X | X | X | X | X | X |  | X |  |
| WF/HK/378.5/01 | X | X | X | X | X | X | X | X |  |
| CK/HK/409.1/02 | X | X | X | X | X | X | X | X |  |
| GS/FJ/bb/03 | X | X | X | X | X | X | X | X |  |
| CK/HB/718/01* | X | X | X | X | X | X | X | X |  |
| DK/GX/xa/01 | X | X | X | X | X | X | X | X |  |
| CK/XJ/16/05 | 1A | 1A | 1A | 1A | 1A | 1A | 1A | 1A |  |
| CK/XJ/17/05 | 1A | 1A | 1A | 1A | 1A | 1A | 1A | 1A |  |
| CK/XJ/68/05 | 1A | 1A | 1A | 1A | 1A | 1A | 1A | 1A |  |
| CK/XJ/54/05 | 1A | 1A | 1A | 1A | 1A | 1A | 1A | 1A |  |
| CK/sy/0606/08 | 1B | 1B | 1B | 1A | 1B | 1B | 1B | 1B |  |
| CK/XJ/78/05 | 1A | 1A | 1A | 1A | 1A | 1A | 1A | 1A |  |
| DGWT/HN/67/05 | 1A | 1A | 1A | 1A | 1A | 1A | 1A | 1A |  |
| DGWT/HN/79/05 | 1A | 1A | 1A | 1A | 1A | 1A | X | 1A |  |
| CK/XJ/53/05 | 1A | 1A | 1A | 1A | 1A | 1A | 1A | 1A |  |
| CK/XJ/67/05 | 1A | 1A | 1A | 1A | 1A | 1A | 1A | 1A |  |
| CK/XJ/27/06 | 1A | 1A | 1A | 1A | 1A | 1A | 1A | 1A |  |
| CK/XJ/28/06 | 1A | 1A | 1A | 1A | 1A | 1A | 1A | 1A |  |
| BJ/01/03 | 1 | 1 | 1B | 1B | 1 | 1B | 1 | 1B |  |
| CK/HB/326/05 | 1B | 1B | 1B | 1B | 1B | 1B | 1B | 1B |  |
| CK/SX/10/06 | 1B | 1B | 1B | 1B | 1B | 1B | 1B | 1B |  |
| CK/LN/A-1/07 | 1B | 1B | 1B | 1B | 1B | 1B | 1B |  |  |
| ML/hd/hn/05 | 1B | 1B | 1B | 1B | 1B | 1B | 1B | 1B |  |
| CK/HB/126/05 | 1B | 1B | 1B | 1B | 1B | 1B | 1B | 1B |  |
| CK/HeN/A-7/06 | 1B | 1B | 1B | 1B | 1B | 1B | 1B | 1B |  |
| CK/SX/2/06 | 1B | 1B | 1B | 1B | 1B | 1B | 1B | 1B |  |
| CK/SD/A-5/06 | 1B | 1B | 1B | 1B | 1B | 1B | 1B | 1B |  |
| CK/NX/24/06 | 1B | 1B | 1B | 1B | 1B | 1B | 1B | 1B |  |
| CK/SD/A-10/06 | 1B | 1B | 1B | 1B | 1B | 1B | 1B | 1B |  |
| CK/LN/A-11/06 | 1B |  | 1B | 1B | 1B | 1B | 1B | 1B |  |
| CK/HB/A-8/09 | 1B | 1 | 1B | 1B | X | 1 | 1B | 1B |  |
| CK/hd/4/08 | 1B | 1 | 1B | 1B | X | 1 | 1B | 1B |  |
| CK/JS/18/08 | 1B | 1 | 1B | 1B | X | 1 | 1B | 1B |  |
| CK/HB/102/05 | 1B | 1B | 1B |  | 1B | 1B | 1B | 1B |  |
| DK/HK/380.5/01 | X | X | X | X | X | X | X | X |  |
| DK/ZJ/bj/02* | X | X | X | X | X | X | X | X |  |
| SW/FJ/1/03 | X | X | X | X | X | X | X | X |  |
| SW/FJ/F1/01 | X | X | X | X | X | X | X | X |  |
| CK/JS/cz1/02 | X | X | X | X | X | X | X | X |  |
| CK/JL/9/04 | 1 | X | X | X | X | X | X | X |  |
| CK/JL/hd/02 | 1 | X | X | X | X | X | X | X |  |
| DK/SH/xj/02 | X | X | X | X | X | X | X | X |  |
| CK/HeN/13/04 | X | X | X | X | X | X | X | X |  |
| CK/HeN/12/04 | X | X | X | X | X | X | X | X |  |
| CK/HeN/01/04 | X | X | X | X | X | X | X | X |  |
| CK/HeN/16/04 | X | X | X | X | X | X | X | X |  |
| SW/HeN/wy/04 | X | X | X | X | X | X | X | X |  |
| CK/AH/39/04 | X | X | X | X | X | X | X | X |  |
| CK/GS/44/04* | X | X | X | X | X | X | X | X |  |
| CK/HN/41/04 | X | X | X | X | X | X | X | X |  |
| GS/GD/72/04 | 2 | 2 | 2 | X | X | X | 2 | 2 |  |
| ML/GX/wt/04* | X | X | X | X | X | X | X | X |  |
| WDK/GD/314/04 | X | X | X | X | X |  | X | X |  |
| CK/HuB/327/04 | X | X | X | X | X | X | X | X |  |
| SN/GX/307/04 | X | X | X | X | X | X | X | X |  |
| CK/HuB/489/04 | X |  | X | X | X | X | X | X |  |
| DK/GD/173/04 | X | X | X | X | X | X | X | X |  |
| PT/GD/C99/05 | X | X | X | X | X | X | X | X |  |
| DK/HK/821/02 | X | X | X | X | X | X | X | X |  |
| HK/213/03 | X | X | X | X | X | X | X | X |  |
| ET/HK/757.2/03 | X | X | X | X | X | X | X | X |  |
| CK/GX/12/04 | X | X | X | X | X | X | X | X |  |
| DK/GX/13/04 | X | X | X | X | X | X | X | X |  |
| ML/hd/Y/03 | 1 | X | 1 | 2 | 2 | 2 | 1 | 1 |  |
| CK/GD/174/04 | 1 | 1 | X | 2 | 2 | 2 | X | 1 |  |
| DK/HuB/wp/03 | 1 | 1 | 1 | 2 | 2 | 2 | 1 | 1 |  |
| GS/JL/hb/03 | 1 | X | X | 2 | X | X | X | 1 |  |
| CK/JX/25/04* | 1 | 1 | X | 2 | 2 | 2 | 1 | 1 | This study |
| BHG/QH/3/05 | 2 | 2 | 2 | 2 | 2 | 2 | 2 | 2 |  |
| PM/LN/7/06 | 2 | 2 | 2 | 2 | 2 | 2 | 2 | 2 |  |
| WDK/LN/8/06 | 2 | 2 | 2 | 2 | 2 | 2 | 2 | 2 |  |
| CK/LN/23/05 | 2 | 2 | 2 | 2 | 2 | 2 | 2 | 2 |  |
| GC/TB/12/06 | 2 | 2 | 2 | 2 | 2 | 2 | 2 | 2 |  |
| BHG/TB/8/06 | 2 | 2 | 2 | 2 | 2 | 2 | 2 | 2 |  |
| BHG/QH/1-HVRI/06 | 2 | 2 | 2 | 2 | 2 | 2 | 2 | 2 |  |
| CK/GD/191/04 | X | X | X | 3 | X | X | X | X |  |
| DK/HN/70/04 | X | X | X | 3 | X | X | X | X |  |
| DK/China/E319-2/03 | X | X | X | 3 | X | 4 | X | X |  |
| CK/GD/178/04 | X | X | X | 3 | X | 4 | X | X |  |
| DK/GD/23/04 | X | X | X | 3 | X | 4 | X | X |  |
| CK/GD/1/05 | 2 | 2 | 2 | 3 | X | X | 2 | 2 |  |
| LH/HK/8550/07 | 3 | X | 3 | 3 | 3 | 3 | 3 | 3 |  |
| CK/HN/8/08 | 3 | X | X | 3 | 3 | 3 | 3 | 3 |  |
| CK/HN/3/07 | 3 | 3 | 3 | 3 | 3 | 3 | 3 | 3 |  |
| DK/HN/3/07 | 3 | 3 | 3 | 3 | 3 | 3 | 3 | 3 |  |
| BCNH/HK/659/08 | 3 | 3 | 3 | 3 | 3 | 3 | 3 | 3 |  |
| DK/HN/8/08* | 3 | X | X | 3 | 3 | 3 | X | X |  |
| SN/SH/10/09 | 3 | B | 3 | 3 | 3 | 3 | 3 | 3 |  |
| HuB/1/10 | 3 | 3 | 3 | 3 | 3 | 3 | 3 | 3 |  |
| GCG/QH/1/09 | 3 | 3 | X | 3 | 3 | 3 | 3 | 3 |  |
| CK/JS/k0402/10 | 3 | X | X | 3 | 3 | 3 | 3 | 3 |  |
| GS/JS/k0403/10 | 3 | X | X | 3 | 3 | 3 | 3 | 3 |  |
| MR/HK/1897/08 | 3 | 3 | 3 | 3 | 3 | 3 | 3 | 3 |  |
| GE/HK/807/08 | 3 | 3 | 3 | 3 | 3 | 3 | 3 | 3 |  |
| GH/HK/3088/07 | 3 | 3 | 3 | 3 | 3 | 3 | 3 | 3 |  |
| MR/HK/1097/08 | 3 | 3 | 3 | 3 | 3 | 3 | 3 | 3 |  |
| GH/HK/1046/08 | 3 | 3 | 3 | 3 | 3 | 3 | 3 | 3 |  |
| DK/ZJ/213/11 | X | 3 | 3 | 3 | 3 | 3 | 3 | 3 |  |
| DK/ZJ/2245/11 | X | X | X | 3 | X | X | X | X |  |
| DK/ZJ/2243/11 | 3 | 3 | 3 | 3 | 3 | 3 | 3 | 3 |  |
| DK/ZJ/2248/11 | 3 | 3 | 3 | 3 | 3 | 3 | 3 | 3 |  |
| DK/ZJ/2242/11 | 3 | 3 | 3 | 3 | 3 | 3 | 3 | 3 |  |
| DK/ZJ/224/11 | 3 | 3 | 3 | 3 | 3 | 3 | 3 | 3 |  |
| DK/ZJ/2244/11 | 3 | 3 | 3 | 3 | 3 | 3 | 3 | 3 |  |
| DK/HN/69/04 | X | X | X | X | X | X | X | X |  |
| DK/AH/56/05 | X | 4 | X | 4 | 4 | 4 | X | 4 |  |
| ML/hd/S/05 | X | X | X | 4 | 4 | 4 | X | 4 |  |
| WDK/HN/021/05 | 4 | 4 | 4 | 4 | 4 | 4 | 4 | 4 |  |
| DK/EC/108/08* | X | X | 4 | 4 | X | 3 | 4 | 4 | This study |
| WDK/HN/211/05 | X | X | X | 4 | X | X | X | 4 |  |
| DK/JX/80/05 | 1 | 1 | 4 | 4 | 4 | 4 | 4 | 4 |  |
| ML/hd/lk/05 | 4 | 4 | 4 | 4 | 4 | 4 | 4 | 4 |  |
| China/GD01/06 | X | 4 | 4 | 4 | 4 | 4 | 4 | 4 |  |
| SH/1/06 | X | 4 | 4 | 4 | 4 | 4 | 4 | 4 |  |
| AV/HK/0828/07 | 3 | 3 | 3 | 4 | 3 | 4 | 3 | 3 |  |
| AV/HK/0719/07 | 3 | 3 | 3 | 4 | 3 | 4 | 3 | 3 |  |
| AV/HK/1993/07 | 3 | 3 | 3 | 4 | 3 | 4 | 3 | 3 |  |
| AV/HK/2372/07 | 3 | 3 | 3 | 4 | 3 | 4 | 3 | 3 |  |
| GX/1/05 | 3 | 3 | 3 | 4 | 3 | 3 | 3 | 3 |  |
| CK/GZ/7/08* | 2 | X | 4 | 4 | 4 | 3 | 3 | 3 | This study |
| China/GD02/06 | 3 | 3 | 3 | 4 | 3 | 3 | 3 | 3 |  |
| GS/YN/4371/06 | 3 | 3 | 3 | 4 | 3 | 4 | 3 | 3 |  |
| DK/YN/47/06 | 3 | 4 | 3 | 4 | 3 | 4 | 3 | 3 |  |
| GS/YN/3798/06 | 3 | 3 | 3 | 4 | 3 | 4 | 3 |  |  |
| DK/HN/11/07 | 3 | 3 | 3 | 4 | 3 | 4 | 3 | 3 |  |
| CK/FJ/1/07 | 3 | 3 | 3 | 4 | 3 | 4 | 3 | 3 |  |
| DK/HN/29/06 | 3 | 3 | 3 | 4 | 3 | 4 | 3 | 3 |  |
| PF/HK/2142/08 | 3 | X | 3 | 4 | 4 | 4 | X | 3 |  |
| CK/HN/21/05 | 4 | 4 | 4 | 4 | 4 | 4 | 4 | 4 |  |
| CK/HuB/2856/07 | X | 4 | 4 | 4 | 4 | 4 | 4 | 4 |  |
| DK/EC/909/09 | X | X | X | 4 | X | 4 | 4 | 4 |  |
| DK/SD/009/08 | X | X | X | 4 | 4 | 4 | X | 4 |  |
| JS/1/07 | X | 4 | 4 | 4 | 4 | 4 | 4 | 4 |  |
| JS/2/07 | X | 4 | 4 | 4 | 4 | 4 | 4 | 4 |  |
| CK/HN/1/09 | X | 4 | 4 | 4 | 4 | 4 | 4 | 4 |  |
| CK/SD/A-1/09* | X | 4 | 4 | 4 | 4 | 1 | 4 | 4 | This study |
| GS/HuB/65/05 | 4 | 4 | 4 | 4 | 4 | 4 | 4 | 4 |  |
| DK/HuB/49/05 | 4 | 4 | 4 | 4 | 4 | 4 | 4 | 4 |  |
| AH/1/05 | 4 | 4 | 4 | 4 | 4 | 4 | 4 | 4 |  |
| DK/HuB/hm01/06 | 4 | 4 | 4 | 4 | 4 | 4 | 4 | 4 |  |
| AH/2/05 | 4 | 4 | 4 | 4 | 4 | 4 | 4 | 4 |  |
| DK/AH/1/06 | 1B | 4 | 4 | 4 | 4 | 4 | 4 | 4 |  |
| CK/HN/3157/06 | X | 4 | 4 | 4 | 4 | 4 | 4 | 4 |  |
| DGWT/HN/3450/06 | X | X | X | 4 | X | 4 | X | X |  |
| DK/HuB/2911/07 | X | 4 | 4 | 4 | 4 | 4 | 4 | 4 |  |
| DK/HN/689/06 | X | 4 | 4 | 4 | 4 | 4 | 4 | 4 |  |
| DK/HN/3315/06 | X | 4 | 4 | 4 | 4 | 4 | 4 | 4 |  |
| CK/HN/1793/07 | X | 4 | X | 4 | 4 | 4 | 4 | 4 |  |
| DK/HN/3340/06 | X | 4 | X | 4 | 4 | 4 | 4 | 4 |  |
| SE/TB/13/06 | 4 | 4 | 4 | 4 | 4 | 4 | 4 | 4 |  |
| CK/SC/81/05 | 4 | 4 | 4 | 4 | 4 | 4 | 4 | 4 |  |
| CK/TB/6/08 | 2 | 4 | 4 | 4 | 4 | 4 | 4 | 4 |  |
| DK/YN/5310/06 | 4 | 4 | 4 | 4 | 4 | 4 | 4 | 4 |  |

*: The [asterisk](javascript:void(0);)s denote the putative recombinant viruses

Lam, T.T., Chong, Y.L., Shi, M., Hon, C.C., Li, J., Martin, D.P. et al. (2013) Systematic phylogenetic analysis of influenza A virus reveals many novel mosaic genome segments. *Infect Genet Evol* **18**: 367-378.

Zhao, Z.M., Shortridge, K.F., Garcia, M., Guan, Y., and Wan, X.F. (2008) Genotypic diversity of H5N1 highly pathogenic avian influenza viruses. *J Gen Virol* **89**: 2182-2193.

Zhou, H., Zhang, A., Chen, H., and Jin, M. (2011) Emergence of novel reassortant H3N2 influenza viruses among ducks in China. *Arch Virol* **156**: 1045-1048.
