# Supplementary material for: Genetic Dynamic Analysis of the Influenza A H5N1 NS1 Gene in China
Source: PLoS One. 2014 Jul 8;9(7):e101384. doi: 10.1371/journal.pone.0101384 (PMC4086889; doi:10.1371/journal.pone.0101384)
Supplement: Table S4 — Estimates of polymorphism and neutrality tests. (DOC) [file pone.0101384.s007.doc]

| **Table S4 Estimates of polymorphism and neutrality tests.** | | | | | | | | | | | | | | |
| --- | --- | --- | --- | --- | --- | --- | --- | --- | --- | --- | --- | --- | --- | --- |
| Segment | Seq | Hp | K | S | η | Pa | Ka | Ks | Ka/Ks | θ^(a)^ | π^(b)^ | Tajima's | D* | F* |
| PB2 | 165 | 157 | 114.239 | 1036 | 1277 | 758 | 0.0092 | 0.247 | 0.037 | 183.90 ^(32.110)^ | 0.05292 ^(0.00313)^ | -1.6086 | -2.67812* | -2.56862* |
| PB1 | 163 | 149 | 50.0828 | 531 | 645 | 378 | 0.0131 | 0.2751 | 0.048 | 93.686 ^(16.529)^ | 0.04197 ^(0.00272)^ | -1.82669* | -3.15561* | -2.99676** |
| PA | 165 | 155 | 123.094 | 1013 | 1274 | 673 | 0.0074 | 0.1928 | 0.038 | 178.341 ^(31.397)^ | 0.06056 ^(0.00155)^ | -1.47556 | -4.11089** | -3.3661** |
| HA | 164 | 155 | 66.7233 | 741 | 880 | 474 | 0.0214 | 0.1248 | 0.171 | 131.595 ^(23.016)^ | 0.04223 ^(0.00146)^ | -1.86142* | -39.96761** | -3.50953** |
| NP | 165 | 158 | 57.1921 | 582 | 706 | 407 | 0.0061 | 0.177 | 0.035 | 102.462 ^(18.039)^ | 0.04027 ^(0.00251)^ | -1.76068 | -3.26082* | -3.01973** |
| NA | 164 | 151 | 52.3312 | 612 | 738 | 393 | 0.0157 | 0.1417 | 0.111 | 107.860 ^(19.009)^ | 0.04122 ^(0.00158)^ | -1.95061* | -4.17417** | -3.69115** |
| MP | 164 | 149 | 27.1824 | 340 | 397 | 228 | 0.010 | 0.0948 | 0.105 | 59.922 ^(10.561)^ | 0.02875 ^(0.00211)^ | -1.98571* | -3.29939** | -3.18524** |
| M1 | 164 | 139 | 21.6339 | 258 | 303 | 180 | 0.061 | 0.114 | 0.054 | 45.470 ^(8.014)^ | 0.02977 ^(0.00236)^ | -1.92522* | -2.8118* | -2.85317* |
| M2 | 156 | 103 | 6.46394 | 99 | 113 | 53 | 0.0160 | 0.0477 | 0.335 | 17.604 ^(3.130)^ | 0.02272 ^(0.00159)^ | -2.1536** | -4.68409** | -4.23597** |
| NS | 163 | 146 | 31.3672 | 419 | 563 | 333 | 0.0302 | 0.0891 | 0.339 | 73.925 ^(13.043)^ | 0.0428 ^(0.00548)^ | -2.23007** | -2.15755 | -2.61457* |
| NS1 | 164 | 142 | 26.1452 | 342 | 464 | 273 | 0.0288 | 0.1256 | 0.229 | 60.275 ^(10.623)^ | 0.04667 ^(0.00582)^ | -2.21265** | -2.18877 | -2.62458* |
| NS2 | 157 | 115 | 11.6924 | 152 | 197 | 114 | 0.0236 | 0.0923 | 0.255 | 26.997 ^(4.795)^ | 0.03572 ^(0.00383)^ | -2.14611** | -2.22819 | -2.61989* |

Hp: Haplotypes

K: Average number of pairwise difference

S:Number of polymorphic (segregating) sites

η:Total number of mutations

Pa: Parsimony informative sites

Ka: Rate of non-synonymous substitutions

Ks: Rate of synonymous substitutions

π:Nucleotide diversity (Jukes and Cantor)

θ:Watterson’s mutation parameter (per sequence calculated from S)

(a):Variance of θ (free recombination)

(b):Standard deviation of π

**:Statistical significance *P*<0.02 (for Fu and Li's test), *P*<0.01（for Tajima's test）

*:Statistical significance *P*<0.05 (for all tests)
