# Supplementary material for: Genetic Dynamic Analysis of the Influenza A H5N1 NS1 Gene in China
Source: PLoS One. 2014 Jul 8;9(7):e101384. doi: 10.1371/journal.pone.0101384 (PMC4086889; doi:10.1371/journal.pone.0101384)
Supplement: Table S5 — Accession numbers of 462 NS1 sequences used in this study. (DOC) [file pone.0101384.s008.doc]

| **Table S5 Accession numbers of 462 NS1 sequences used in this study.** | | | |
| --- | --- | --- | --- |
| **Virus** | **Abbreviation** | **Allele** | **accession** |
| A/Anhui/1/2005 | AH/1/05 | A | AEO88942 |
| A/Anhui/1/2006 | AH/1/06 | A | AEO89068 |
| A/Anhui/1/2007 | AH/1/07 | A | AEO89085 |
| A/Anhui/2/2005 | AH/2/05 | A | AEO88950 |
| A/Bar-headed_Goose/Qinghai/59/05 | BHG/QH/59/05 | A | AAZ14171 |
| A/Bar-headed_Goose/Qinghai/60/05 | BHG/QH/60/05 | A | AAZ14177 |
| A/Bar-headed_Goose/Qinghai/68/05 | BHG/QH/68/05 | A | AAZ14173 |
| A/Beijing/01/2003 | BJ/01/03 | A | ABQ58984 |
| A/Beijing/1/2009 | BJ/1/09 | A | AEO89121 |
| A/Chicken/Hong_Kong/220/97 | CK/HK/220/97 | A | AAC32082 |
| A/Chicken/Hong_Kong/258/97 | CK/HK/258/97 | A | AAF02341 |
| A/Chicken/Hong_Kong/728/97 | CK/HK/728/97 | A | AAF02345 |
| A/Chicken/Hong_Kong/786/97 | CK/HK/786/97 | A | AAF02347 |
| A/Chicken/Hong_Kong/829.2/01 | CK/HK/829.2/01 | A | AAO52921 |
| A/Chicken/Hong_Kong/858.3/01 | CK/HK/858.3/01 | A | AAO52923 |
| A/Chicken/Hong_Kong/866.3/01 | CK/HK/866.3/01 | A | AAO52924 |
| A/Chicken/Hong_Kong/879.1/01 | CK/HK/879.1/01 | A | AAO52926 |
| A/Chicken/Hong_Kong/891.1/01 | CK/HK/891.1/01 | A | AAO52929 |
| A/Chicken/Hong_Kong/y388/97 | CK/HK/y388/97 | A | AAF02343 |
| A/Chicken/HongKong/FY150/01-MB | CK/HK/FY150/01 | A | AAO46835 |
| A/Chicken/HongKong/NT873.3/01-MB | CK/HK/NT873.3/01 | A | AAO46833 |
| A/Chicken/HongKong/YU822.2/01-MB | CK/HK/YU822.2/01 | A | AAO46839 |
| A/Chicken/Shantou/810/05 | CK/ST/810/05 | A | AAZ14199 |
| A/Chicken/Yunnan/447/05 | CK/YN/447/05 | A | AAZ14195 |
| A/Chinese_pond_heron/Hong_Kong/18/2005 | CPH/HK/18/05 | A | ABC68573 |
| A/Ck/HK/2133.1/2003 | CK/HK/2133.1/03 | A | AAT73435 |
| A/Ck/HK/31.2/2002 | CK/HK/31.2/02 | A | AAT73419 |
| A/Ck/HK/31.4/02 | CK/HK/31.4/02 | A | AAT39023 |
| A/Ck/HK/61.9/02 | CK/HK/61.9/02 | A | AAT39024 |
| A/Ck/HK/FY157/2003 | CK/HK/FY157/03 | A | AAT73431 |
| A/Ck/HK/NT93/2003 | CK/HK/NT93/03 | A | AAT73437 |
| A/Ck/HK/WF157/2003 | CK/HK/WF157/03 | A | AAT73441 |
| A/Ck/HK/YU324/2003 | CK/HK/YU324/03 | A | AAT73433 |
| A/Ck/ST/4231/2003 | CK/ST/4231/03 | A | AAT73459 |
| A/Daurian_starling/Hong_Kong/1532/2007 | DT/HK/1532/07 | A | ACJ26258 |
| A/Dk/HN/101/2004 | DK/HN/101/04 | A | AAT73473 |
| A/Dk/HN/303/2004 | DK/HN/303/04 | A | AAT73471 |
| A/Dk/HN/5806/2003 | DK/HN/5806/03 | A | AAT73455 |
| A/Dk/ST/4003/2003 | DK/ST/4003/03 | A | AAT73457 |
| A/Dk/YN/6255/2003 | DK/YN/6255/03 | A | AAT73461 |
| A/Dk/YN/6445/2003 | DK/YN/6445/03 | A | AAT73463 |
| A/Duck/Anyang/AVL-1/2001 | DK/ay/AVL-1/01 | A | AAM49563 |
| A/Duck/Fujian/1734/05 | DK/FJ/1734/05 | A | AAZ14205 |
| A/Duck/Hong_Kong/2986.1/2000 | DK/HK/2986.1/00 | A | AAL31410 |
| A/Duck/Hong_Kong/380.5/2001 | DK/HK/380.5/01 | A | AAL75851 |
| A/Duck/Hong_Kong/573.4/01 | DK/HK/573.4/01 | A | AAO52933 |
| A/Duck/Hong_Kong/646.3/01 | DK/HK/646.3/01 | A | AAO52934 |
| A/Duck/Hong_Kong/p46/97 | DK/HK/p46/97 | A | AAF02351 |
| A/Duck/Hong_Kong/ww381/2000 | DK/HK/ww381/00 | B | AAL31407 |
| A/Duck/Hong_Kong/ww461/2000 | DK/HK/ww461/00 | B | AAL31408 |
| A/Fujian/1/2005 | FJ/1/05 | A | AEO88974 |
| A/G.H/HK/793.1/02 | G.H/HK/793.1/02 | A | AAT39021 |
| A/Gf/HK/38/2002 | Gf/HK/38/02 | A | AAT73417 |
| A/Goose/Hong_Kong/3014.8/2000 | GS/HK/3014.8/00 | A | AAL31411 |
| A/Goose/Hong_Kong/76.1/01 | GS/HK/76.1/01 | A | AAO52931 |
| A/Goose/Hong_Kong/ww100/01 | GS/HK/ww100/01 | A | AAO52932 |
| A/Goose/Hong_Kong/ww26/2000 | GS/HK/ww26/00 | B | AAL31405 |
| A/Goose/Hong_Kong/ww491/2000 | GS/HK/ww491/00 | B | AAL31409 |
| A/Goose/Shantou/1621/05 | GS/ST/1621/05 | A | AAZ14203 |
| A/Great_black-headed_gull/Qinghai/2/2005 | GBHG/QH/2/05 | A | AAZ14175 |
| A/Guangdong/01/2006 | GD/01/06 | A | AEO89015 |
| A/Guangdong/1/2008 | GD/1/08 | A | AEO89112 |
| A/Guangdong/2/2006 | GD/2/06 | A | AEO89051 |
| A/Guangxi/1/2005 | GX/1/05 | A | AEO88958 |
| A/Guangxi/1/2008 | GX/1/08 | A | AEO89103 |
| A/Guangxi/1/2009 | GX/1/09 | A | AEO89166 |
| A/Guinea_fowl/Shantou/1341/2006 | GF/st/1341/06 | A | ABL08783 |
| A/Guizhou/1/2009 | GZ/1/09 | A | AEO89157 |
| A/HK/212/03 | HK/212/03 | A | AAT39113 |
| A/Hong_Kong/156/97 | HK/156/97 | A | CAC04091 |
| A/Hong_Kong/213/2003 | HK/213/03 | A | AAT39114 |
| A/Hong_Kong/378.1/2001 | HK/378.1/01 | A | ACZ47221 |
| A/Hong_Kong/481/97 | HK/481/97 | A | AAF75115 |
| A/Hong_Kong/482/97 | HK/482/97 | A | AAK49303 |
| A/Hong_Kong/483/1997 | HK/483/1997 | A | AAK49305 |
| A/Hong_Kong/485/1997 | HK/485/1997 | A | AAF74342 |
| A/Hong_Kong/486/97 | HK/486/97 | A | AAF75117 |
| A/Hong_Kong/514/97 | HK/514/97 | A | AAK49309 |
| A/Hong_Kong/532/1997 | HK/532/1997 | A | AAK49311 |
| A/Hong_Kong/538/97 | HK/538/97 | A | AAK49313 |
| A/Hong_Kong/542/97 | HK/542/97 | A | AAK49315 |
| A/Hong_Kong/6841/2010 | HK/6841/10 | A | ADR30458 |
| A/HongKong/97/98 | HK/97/98 | A | AAK49317 |
| A/Hubei/1/2006 | HuB/1/06 | A | AEO89033 |
| A/Hubei/1/2010 | HuB/1/10 | A | AEO89186 |
| A/Hunan/1/2006 | HN/1/06 | A | AEO88999 |
| A/Hunan/1/2008 | HN/1/08 | A | AEO89094 |
| A/Hunan/1/2009 | HN/1/09 | A | AEO89139 |
| A/Hunan/2/2009 | HN/2/09 | A | AEO89175 |
| A/Japanese_white_eye/Hong_Kong/1038/2006 | JHE/HK/1038/06 | A | ABL08545 |
| A/Jiangsu/2/2007 | JS2/07 | A | ACB87568 |
| A/Jiangxi/1/2005 | JX/1/05 | A | AEO88966 |
| A/Pheasant/HongKong/FY155/01-MB | PhT/HK/FY155/01 | A | AAO46837 |
| A/Quail/Shantou/911/05 | QL/ST/911/05 | A | AAZ14200 |
| A/SCk/HK/YU100/2002 | SCk/HK/YU100/02 | A | AAT73423 |
| A/Shandong/1/2009 | SD/1/09 | A | AEO89130 |
| A/Sichuan/1/2006 | SC/1/06 | A | AEO88991 |
| A/Sichuan/3/2006 | SC/3/06 | A | AEO89042 |
| A/Silky_Chicken/Hong_Kong/SF189/01 | SCK/HK/SF189/01 | A | AAO52914 |
| A/Xinjiang/1/2009 | XJ/1/09 | A | AEO89148 |
| A/Zhejiang/1/2006 | ZJ/1/06 | A | AEO89007 |
| A/bar-headed_goose/Qinghai/1-HVRI/2006 | BHG/QH/1-HVRI/06 | A | ADG59331 |
| A/bar-headed_goose/Tibet/8/2006 | BHG/Tibet/8/06 | A | ADG59317 |
| A/black_headed_gull/HK/12.1/2003 | BHGL/HK/12.1/03 | A | AAT73443 |
| A/black-crowned_night_heron/Hong_Kong/659/2008 | BCNH/HK/659/08 | A | ACJ26324 |
| A/blue_magpie/Hong_Kong/1993/2007 | BM/HK/1993/07 | A | ACJ26159 |
| A/chicken/Anhui/1089/2007 | CK/AH/1089/07 | A | ACN39379 |
| A/chicken/Anhui/T6/2006 | CK/AH/T6/06 | A | ABW24491 |
| A/chicken/Fujian/1042/2005 | CK/FJ/1042/05 | A | ABC68524 |
| A/chicken/Fujian/11933/2005 | CK/FJ/11933/05 | A | ABL08697 |
| A/chicken/Fujian/9821/2005 | CK/FJ/9821/05 | A | ABL08683 |
| A/chicken/Gansu/44/2004 | CK/GS/44/04 | A | ADG59335 |
| A/chicken/Guangdong/174/04 | CK/GD/174/04 | A | AAT37568 |
| A/chicken/Guangdong/178/04 | CK/GD/178/04 | A | AAW59404 |
| A/chicken/Guangdong/191/04 | CK/GD/191/04 | A | AAW59385 |
| A/chicken/Guangxi/12/2004 | CK/GX/12/04 | A | ABD14789 |
| A/chicken/Guangxi/2439/2004 | CK/GX/2439/04 | A | ABC68541 |
| A/chicken/Guangxi/2461/2004 | CK/GX/2461/04 | A | ABC68543 |
| A/chicken/Guangxi/3154/2005 | CK/GX/3154/05 | A | ABL08723 |
| A/chicken/Guangxi/4989/2005 | CK/GX/4989/05 | A | ABL08611 |
| A/chicken/Guangxi/604/2005 | CK/GX/604/05 | A | ABC68546 |
| A/chicken/Guiyang/2147/2005 | CK/gy/2147/05 | A | ABL08585 |
| A/chicken/Guiyang/2173/2005 | CK/gy/2173/05 | A | ABL08587 |
| A/chicken/Guiyang/29/2006 | CK/gy/29/06 | A | ABL08643 |
| A/chicken/Guiyang/3055/2005 | CK/gy/3055/05 | A | ABL08775 |
| A/chicken/Guiyang/3721/2005 | CK/gy/3721/05 | A | ABL08639 |
| A/chicken/Guiyang/441/2006 | CK/gy/441/06 | A | ABL08581 |
| A/chicken/Guizhou/7/2008 | CK/GZ/7/08 | A | ADG59321 |
| A/chicken/Hebei/102/2005 | CK/HB/102/05 | A | ABX10500 |
| A/chicken/Hebei/108/02 | CK/HB/108/02 | A | ABC74399 |
| A/chicken/Hebei/126/2005 | CK/HB/126/05 | A | ABX10492 |
| A/chicken/Hebei/326/2005 | CK/HB/326/05 | A | ABC74401 |
| A/chicken/Hebei/706/2005 | CK/HB/706/05 | A | ABX10507 |
| A/chicken/Hebei/718/2001 | CK/HB/718/01 | A | ABC74403 |
| A/chicken/Hebei/A-8/2009 | CK/HB/A-8/09 | A | ADG59305 |
| A/chicken/Henan/01/2004 | CK/HeN/01/04 | A | AAX53538 |
| A/chicken/Henan/12/2004 | CK/HeN/12/04 | A | AAX53543 |
| A/chicken/Henan/13/2004 | CK/HeN/13/04 | A | AAX53545 |
| A/chicken/Henan/1362/2006 | CK/HeN/1362/06 | A | ACN39381 |
| A/chicken/Henan/A-7/2006 | CK/HeN/A-7/06 | A | ADG59345 |
| A/chicken/Hong_Kong/3123.1/2002 | CK/HK/3123.1/02 | A | ABC68574 |
| A/chicken/Hong_Kong/409.1/2002 | CK/HK/409.1/02 | A | AAT39027 |
| A/chicken/Hong_Kong/86.3/2002 | CK/HK/86.3/02 | A | ABC68575 |
| A/chicken/Hong_Kong/915/97 | CK/HK/915/97 | A | AAF02349 |
| A/chicken/Hong_Kong/YU562/2001 | CK/HK/YU562/01 | A | AAO46841 |
| A/chicken/Huadong/4/2008 | CK/Hd/4/08 | A | AFR53952 |
| A/chicken/Hubei/2856/2007 | CK/HuB/2856/07 | A | ACN39397 |
| A/chicken/Hubei/3002/2007 | CK/HuB/3002/07 | A | ACN39401 |
| A/chicken/Hubei/327/2004 | CK/HuB/327/04 | A | AAT90838 |
| A/chicken/Hubei/489/2004 | CK/HuB/489/04 | A | AAV48547 |
| A/chicken/Hubei/wf/2002 | CK/HuB/wf/02 | A | ABI94745 |
| A/chicken/Hubei/wh/1997 | CK/HuB/wh/1997 | A | ABI94757 |
| A/chicken/Hubei/wj/1997 | CK/HuB/wj/1997 | A | ABI96722 |
| A/chicken/Hubei/wk/1997 | CK/HuB/wk/1997 | A | ABI96735 |
| A/chicken/Hubei/wm/1997 | CK/HuB/wm/1997 | A | ABI96753 |
| A/chicken/Hubei/wo/2003 | CK/HuB/wo/03 | A | ABI98905 |
| A/chicken/Hunan/1/2009 | CK/HN/1/09 | A | ADG59299 |
| A/chicken/Hunan/1793/2007 | CK/HN/1793/07 | A | ACN39389 |
| A/chicken/Hunan/21/2005 | CK/HN/21/05 | A | ADG59349 |
| A/chicken/Hunan/23/2002 | CK/HN/23/02 | A | ACA47829 |
| A/chicken/Hunan/3157/2006 | CK/HN/3157/06 | A | ACN39403 |
| A/chicken/Hunan/41/2004 | CK/HN/41/04 | A | ADG59369 |
| A/chicken/Hunan/8/2008 | CK/HN/8/08 | A | ACZ05817 |
| A/chicken/Jiangsu/18/2008 | CK/JS18/08 | A | ADG59313 |
| A/chicken/Jiangsu/cz1/2002 | CK/JS/cz1/02 | A | ABI96771 |
| A/chicken/Jiangsu/k0402/2010 | CK/JS/k0402/10 | A | AFC98308 |
| A/chicken/Jiangxi/25/2004 | CK/JX/25/04 | A | ADG59549 |
| A/chicken/Jilin/ha/2003 | CK/JL/ha/03 | A | ABI98916 |
| A/chicken/Jilin/hd/2002 | CK/JL/hd/02 | A | ABI98922 |
| A/chicken/Jilin/he/2002 | CK/JL/he/02 | A | ABI97317 |
| A/chicken/Jilin/hf/2002 | CK/JL/hf/02 | A | ABI98932 |
| A/chicken/Jilin/hg/2002 | CK/JL/hg/02 | A | ABI97326 |
| A/chicken/Jilin/hh/2002 | CK/JL/hh/02 | A | ABI97338 |
| A/chicken/Jilin/hl/2004 | CK/JL/hl/04 | A | ABJ52570 |
| A/chicken/Jilin/hn/2003 | CK/JL/hn/03 | A | ABJ80588 |
| A/chicken/Jilin/ho/2003 | CK/JL/ho/03 | A | ABK00084 |
| A/chicken/Jilin/hp/2003 | CK/JL/hp/03 | A | ABK00091 |
| A/chicken/Jilin/xv/2002 | CK/JL/xv/02 | A | ABK00107 |
| A/chicken/Jilin/xw/2003 | CK/JL/xw/03 | A | ABI98941 |
| A/chicken/Liaoning/23/2005 | CK/LN/23/05 | A | ADG59545 |
| A/chicken/Liaoning/A-11/2006 | CK/LN/A-11/06 | A | ADG59379 |
| A/chicken/Ningxia/24/2006 | CK/NX/24/06 | A | ADG59333 |
| A/chicken/Shandong/A-1/2009 | CK/SD/A-1/09 | A | ADG59329 |
| A/chicken/Shandong/A-10/2006 | CK/SD/A-10/06 | A | ADG59311 |
| A/chicken/Shandong/A-5/2006 | CK/SD/A-5/06 | A | ADG59355 |
| A/chicken/Shantou/1233/2006 | CK/st/1233/06 | A | ABL08655 |
| A/chicken/Shantou/2535/2001 | CK/st/2535/01 | A | ACA47466 |
| A/chicken/Shantou/3744/2003 | CK/st/3744/03 | A | ACA47796 |
| A/chicken/Shantou/3840/2006 | CK/st/3840/06 | A | ABL08661 |
| A/chicken/Shantou/3900/2002 | CK/st/3900/02 | A | ACA47686 |
| A/chicken/Shantou/5746/2001 | CK/st/5746/01 | A | ACA47565 |
| A/chicken/Shantou/904/2001 | CK/st/904/01 | A | ACA47422 |
| A/chicken/Shanxi/10/2006 | CK/SX/10/06 | A | ADG59357 |
| A/chicken/Shanxi/2/2006 | CK/SX/2/06 | A | ABK34770 |
| A/chicken/Sheny/0606/2008 | CK/sy/0606/08 | A | AEX30616 |
| A/chicken/Sichuan/81/2005 | CK/SC/81/05 | A | ADG59285 |
| A/chicken/Tibet/6/2008 | CK/TB/6/08 | A | ADG59327 |
| A/chicken/Xinjiang/16/2005 | CK/XJ/16/05 | A | ADG59297 |
| A/chicken/Xinjiang/17/2005 | CK/XJ/17/05 | A | ADG59315 |
| A/chicken/Xinjiang/27/2006 | CK/XJ/27/06 | A | ADG59353 |
| A/chicken/Xinjiang/28/2006 | CK/XJ/28/06 | A | ADG59341 |
| A/chicken/Xinjiang/53/2005 | CK/XJ/53/05 | A | ADG59365 |
| A/chicken/Xinjiang/54/2005 | CK/XJ/54/05 | A | ADG59373 |
| A/chicken/Xinjiang/68/2005 | CK/XJ/68/05 | A | ADG59361 |
| A/chicken/Xinjiang/78/2005 | CK/XJ/78/05 | A | ADG59319 |
| A/chicken/Yunnan/1215/2002 | CK/YN/1215/02 | A | ACA47950 |
| A/chicken/Yunnan/1628/2003 | CK/YN/1628/03 | A | ACA48038 |
| A/chicken/Yunnan/207/2004 | CK/YN/207/04 | A | ACA48170 |
| A/chicken/Yunnan/6797/2003 | CK/YN/6797/03 | A | ACA48137 |
| A/chukar/Shantou/4690/2003 | chukar/st/4690/03 | A | ACA47807 |
| A/common_buzzard/Hong_Kong/9213/2007 | CB/HK/9213/07 | A | ACJ26313 |
| A/common_kestrel/Hong_Kong/2372/2007 | CKL/HK/2372/07 | A | ACJ26181 |
| A/common_magpie/Hong_Kong/2125/2006 | CM/HK/2125/06 | A | ABL08547 |
| A/common_magpie/Hong_Kong/645/2006 | CM/HK/645/06 | A | ABL08571 |
| A/condor/Guangdong/139/2003 | condor/GD/139/03 | A | AFK13867 |
| A/crested_goshawk/Hong_Kong/458/2007 | CG/HK/458/07 | A | ACJ26093 |
| A/crested_myna/Hong_Kong/540/2006 | CMA/HK/540/06 | A | ABL08569 |
| A/domestic_green-winged_teal/Hunan/3450/2006 | DGWT/HN/3450/06 | B | AGH30426 |
| A/domestic_green-winged_teal/Hunan/67/2005 | DGWT/HN/67/05 | A | ABZ91700 |
| A/domestic_green-winged_teal/Hunan/79/2005 | DGWT/HN/79/05 | A | ABZ91684 |
| A/duck/Anhui/1/06 | DK/AH/1/06 | A | ADG59371 |
| A/duck/Anhui/56/2005 | DK/AH/56/05 | A | ADG59303 |
| A/duck/China/E319-2/03 | DK/China/E319-2/03 | A | AAR99625 |
| A/duck/Eastern_China/108/2008 | DK/EC/108/08 | A | ADD10563 |
| A/duck/Eastern_China/909/2009 | DK/EC/909/09 | A | ADD10553 |
| A/duck/Eastern_China/S115/2008 | DK/EC/S115/08 | A | ADU58434 |
| A/duck/Eastern_China/Yzd/2009 | DK/EC/Yzd/09 | A | ADU58462 |
| A/duck/Fujian/10160/2005 | DK/FJ/10160/05 | A | ABL08795 |
| A/duck/Fujian/11311/2005 | DK/FJ/11311/05 | A | ABL08695 |
| A/duck/Fujian/12032/2005 | DK/FJ/12032/05 | A | ABL08699 |
| A/duck/Fujian/13/2002 | DK/FJ/13/02 | A | AAT12107 |
| A/duck/Fujian/17/2001 | DK/FJ/17/01 | A | AAT12108 |
| A/duck/Fujian/19/2000 | DK/FJ/19/00 | A | AAT12109 |
| A/duck/Fujian/668/2006 | DK/FJ/668/06 | A | ABL08703 |
| A/duck/Fujian/671/2006 | DK/FJ/671/06 | A | ABL08705 |
| A/duck/Fujian/897/2005 | DK/FJ/897/05 | A | ABC68523 |
| A/duck/Fujian/9651/2005 | DK/FJ/9651/05 | A | ABL08679 |
| A/duck/Guangdong/01/2001 | DK/GD/01/01 | A | AAT12110 |
| A/duck/Guangdong/07/2000 | DK/GD/07/00 | B | AAT12111 |
| A/duck/Guangdong/12/2000 | DK/GD/12/00 | B | AAT12112 |
| A/duck/Guangdong/173/04 | DK/GD/173/04 | A | AAW59412 |
| A/duck/Guangdong/22/2002 | DK/GD/22/02 | A | AAT12113 |
| A/duck/Guangdong/40/2000 | DK/GD/40/00 | A | AAT12114 |
| A/duck/Guangdong/DG-01/2005 | DK/GD/DG-01/05 | A | ABE96873 |
| A/duck/Guangxi/07/1999 | DK/GX/07/1999 | A | AAT12115 |
| A/duck/Guangxi/12/2003 | DK/GX/12/03 | A | ABW95943 |
| A/duck/Guangxi/13/2004 | DK/GX/13/04 | A | ABD14790 |
| A/duck/Guangxi/1378/2004 | DK/GX/1378/04 | A | ABC68532 |
| A/duck/Guangxi/1436/2006 | DK/GX/1436/06 | A | ABL08829 |
| A/duck/Guangxi/1793/2004 | DK/GX/1793/04 | A | ABC68535 |
| A/duck/Guangxi/1830/2006 | DK/GX/1830/06 | A | ABL08837 |
| A/duck/Guangxi/2143/2006 | DK/GX/2143/06 | A | ABL08843 |
| A/duck/Guangxi/22/2001 | DK/GX/22/01 | A | AAT12116 |
| A/duck/Guangxi/2291/2004 | DK/GX/2291/04 | A | ABC68538 |
| A/duck/Guangxi/27/2003 | DK/GX/27/03 | A | ABW95954 |
| A/duck/Guangxi/2775/2005 | DK/GX/2775/05 | A | ABL08715 |
| A/duck/Guangxi/288/2006 | DK/GX/288/06 | A | ABL08627 |
| A/duck/Guangxi/3364/2005 | DK/GX/3364/05 | A | ABL08727 |
| A/duck/Guangxi/35/2001 | DK/GX/35/01 | A | AAT12117 |
| A/duck/Guangxi/3548/2005 | DK/GX/3548/05 | A | ABL08729 |
| A/duck/Guangxi/380/2004 | DK/GX/380/04 | A | ABC68526 |
| A/duck/Guangxi/3819/2005 | DK/GX/3819/05 | A | ABL08737 |
| A/duck/Guangxi/4016/2005 | DK/GX/4016/05 | A | ABL08739 |
| A/duck/Guangxi/4184/2005 | DK/GX/4184/05 | A | ABL08741 |
| A/duck/Guangxi/4428/2005 | DK/GX/4428/05 | A | ABL08605 |
| A/duck/Guangxi/50/2001 | DK/GX/50/01 | A | AAT12118 |
| A/duck/Guangxi/5075/2005 | DK/GX/5075/05 | A | ABL08613 |
| A/duck/Guangxi/53/2002 | DK/GX/53/02 | A | AAT12119 |
| A/duck/Guangxi/668/2004 | DK/GX/668/04 | A | ABC68527 |
| A/duck/Guangxi/744/2006 | DK/GX/744/06 | A | ABL08635 |
| A/duck/Guangxi/793/2005 | DK/GX/793/05 | A | ABC68547 |
| A/duck/Guangxi/89/2006 | DK/GX/89/06 | A | ABL08583 |
| A/duck/Guangxi/951/2005 | DK/GX/951/05 | A | ABC68548 |
| A/duck/Guangxi/xa/2001 | DK/GX/xa/01 | A | ABJ09469 |
| A/duck/Guangzhou/20/2005 | DK/GZ/20/05 | A | ABC68549 |
| A/duck/Guiyang/1722/2006 | DK/gy/1722/06 | A | ABL08805 |
| A/duck/Guiyang/2231/2005 | DK/gy/2231/05 | A | ABL08577 |
| A/duck/Guiyang/293/2006 | DK/gy/293/06 | A | ABL08645 |
| A/duck/Guiyang/3009/2005 | DK/gy/3009/05 | A | ABL08773 |
| A/duck/Guiyang/3834/2005 | DK/gy/3834/05 | A | ABL08769 |
| A/duck/Henan/1647/2006 | DK/HeN/1647/06 | A | ACN39383 |
| A/duck/Henan/1652/2006 | DK/HeN/1652/06 | A | ACN39387 |
| A/duck/Hong_Kong/140/1998 | DK/HK/140/1998 | A | ACZ47323 |
| A/duck/Hong_Kong/2986.1-2/2000 | DK/HK/2986.1-2/00 | A | ACZ47215 |
| A/duck/Hubei/2911/2007 | DK/HuB/2911/07 | A | ACN39399 |
| A/duck/Hubei/49/2005 | DK/HuB/49/05 | A | ADG59289 |
| A/duck/Hubei/Hangmei01/2006 | DK/HuB/Hangmei01/06 | A | ACF16405 |
| A/duck/Hubei/wp/2003 | DK/HuB/wp/03 | A | ABJ09479 |
| A/duck/Hunan/11/2007 | DK/HN/11/07 | A | ADG59309 |
| A/duck/Hunan/114/05 | DK/HN/114/05 | A | AAZ14207 |
| A/duck/Hunan/1204/2006 | DK/HN/1204/06 | A | ABL08809 |
| A/duck/Hunan/1340/2002 | DK/HN/1340/02 | A | ACA47851 |
| A/duck/Hunan/1608/2005 | DK/HN/1608/05 | A | ABC68560 |
| A/duck/Hunan/1964/2007 | DK/HN/1964/07 | A | ACN39393 |
| A/duck/Hunan/1994/2007 | DK/HN/1994/07 | A | ACN39395 |
| A/duck/Hunan/29/2006 | DK/HN/29/06 | A | ADG59351 |
| A/duck/Hunan/3/2007 | DK/HN/3/07 | A | ACZ05819 |
| A/duck/Hunan/300/2003 | DK/HN/300/03 | A | ACA47862 |
| A/duck/Hunan/324/2006 | DK/HN/324/06 | A | ABL08671 |
| A/duck/Hunan/3315/2006 | DK/HN/3315/06 | A | ACN39405 |
| A/duck/Hunan/3340/2006 | DK/HN/3340/06 | A | ACN39407 |
| A/duck/Hunan/533/2004 | DK/HN/533/04 | A | ACA47906 |
| A/duck/Hunan/5472/2005 | DK/HN/5472/05 | A | ABL08669 |
| A/duck/Hunan/689/2006 | DK/HN/689/06 | A | ACN39377 |
| A/duck/Hunan/69/2004 | DK/HN/69/04 | A | ADG59377 |
| A/duck/Hunan/70/2004 | DK/HN/70/04 | A | ADG59375 |
| A/duck/Hunan/733/2004 | DK/HN/733/04 | A | ACA47917 |
| A/duck/Hunan/782/2003 | DK/HN/782/03 | A | ACA47884 |
| A/duck/Hunan/795/2002 | DK/HN/795/02 | A | ACA47840 |
| A/duck/Hunan/8/2008 | DK/HN/8/08 | A | ACZ05815 |
| A/duck/Hunan/856/2006 | DK/HN/856/06 | A | ABL08675 |
| A/duck/Hunan/S4030/2011 | DK/HN/S4030/2011 | A | AGO87176 |
| A/duck/Hunan/S4150/2011 | DK/HN/S4150/2011 | A | AGO87248 |
| A/duck/Hunan/S4220/2011 | DK/HN/S4220/2011 | A | AGO87260 |
| A/duck/Hunan/S4234/2011 | DK/HN/S4234/2011 | A | AGO87272 |
| A/duck/Jiangxi/80/2005 | DK/JX/80/05 | A | ADG59343 |
| A/duck/Shandong/009/2008 | DK/SD/009/08 | A | ADC97020 |
| A/duck/Shandong/093/2004 | DK/SD/093/04 | A | AAW72233 |
| A/duck/Shanghai/08/2001 | DK/SH/08/01 | B | AAT12120 |
| A/duck/Shanghai/13/2001 | DK/SH/13/01 | A | AAT12121 |
| A/duck/Shanghai/35/2002 | DK/SH/35/02 | A | AAT12122 |
| A/duck/Shanghai/37/2002 | DK/SH/37/02 | A | AAT12123 |
| A/duck/Shanghai/38/2001 | DK/SH/38/01 | A | AAT12124 |
| A/duck/Shanghai/xj/2002 | DK/SH/xj/02 | A | ABJ09491 |
| A/duck/Shantou/13323/2005 | DK/ST/13323/05 | A | ABL08651 |
| A/duck/Shantou/1437/2001 | DK/ST/1437/01 | A | ACA47444 |
| A/duck/Shantou/195/2001 | DK/ST/195/01 | B | ACA47411 |
| A/duck/Shantou/4610/2003 | DK/ST/4610/03 | A | ABC68562 |
| A/duck/Shantou/5526/2001 | DK/ST/5526/01 | A | ACA47543 |
| A/duck/Shantou/700/2002 | DK/ST/700/02 | A | ACA47620 |
| A/duck/Sheyang/1/2005 | DK/sy/1/05 | A | ABD23023 |
| A/duck/Yunnan/215/2003 | DK/YN/215/03 | A | ACA47972 |
| A/duck/Yunnan/4072/2003 | DK/YN/4072/03 | A | ACA48049 |
| A/duck/Yunnan/4400/2005 | DK/YN/4400/05 | A | ABL08751 |
| A/duck/Yunnan/47/2006 | DK/YN/47/06 | A | ADG59339 |
| A/duck/Yunnan/4873/2006 | DK/YN/4873/06 | A | ACH85382 |
| A/duck/Yunnan/5133/2005 | DK/YN/5133/05 | A | ABL08599 |
| A/duck/Yunnan/5251/2005 | DK/YN/5251/05 | A | ABL08591 |
| A/duck/Yunnan/5310/2006 | DK/YN/5310/06 | A | ACH85393 |
| A/duck/Yunnan/5820/2005 | DK/YN/5820/05 | A | ABL08593 |
| A/duck/Yunnan/5877/2005 | DK/YN/5877/05 | A | ABL08759 |
| A/duck/Yunnan/6332/2005 | DK/YN/6332/05 | A | ABL08763 |
| A/duck/Yunnan/6490/2006 | DK/YN/6490/06 | A | ACH85404 |
| A/duck/Yunnan/862/2002 | DK/YN/862/02 | A | ACA47928 |
| A/duck/Zhejiang/11/2000 | DK/ZJ/11/00 | B | AAT12125 |
| A/duck/Zhejiang/213/2011 | DK/ZJ/213/2011 | A | AEO52360 |
| A/duck/Zhejiang/2245/2011 | DK/ZJ/2245/2011 | A | AEO52310 |
| A/duck/Zhejiang/2248/2011 | DK/ZJ/2248/2011 | A | AEO52300 |
| A/duck/Zhejiang/52/2000 | DK/ZJ/52/00 | A | AAT12126 |
| A/duck/Zhejiang/bj/2002 | DK/ZJ/bj/02 | A | ABJ09501 |
| A/feral_pigeon/HK/862.7/2002 | FP/HK/862.7/02 | A | AAT73447 |
| A/goose/Fujian/bb/2003 | GS/FJ/bb/03 | A | ABJ09514 |
| A/goose/Guangdong/1/1996 | GS/GD/1/96 | B | AAD51930 |
| A/goose/Guangdong/3/1997 | GS/GD/3/1997 | B | AAK38762 |
| A/goose/Guangdong/72/2004 | GS/GD/72/04 | A | ADG59323 |
| A/goose/Guangdong/xb/2001 | GS/GD/xb/01 | A | ABJ09521 |
| A/goose/Guangxi/1097/2004 | GS/GX/1097/04 | A | ABC68529 |
| A/goose/Guangxi/1198/2004 | GS/GX/1198/04 | A | ABC68530 |
| A/goose/Guangxi/1458/2006 | GS/GX/1458/06 | A | ABL08831 |
| A/goose/Guangxi/1898/2006 | GS/GX/1898/06 | A | ABL08839 |
| A/goose/Guangxi/2112/2004 | GS/GX/2112/04 | A | ABC68537 |
| A/goose/Guangxi/224/2006 | GS/GX/224/06 | A | ABL08625 |
| A/goose/Guangxi/2383/2004 | GS/GX/2383/04 | A | ABC68539 |
| A/goose/Guangxi/3316/2005 | GS/GX/3316/05 | A | ABL08725 |
| A/goose/Guangxi/3714/2005 | GS/GX/3714/05 | A | ABL08731 |
| A/goose/Guangxi/4289/2005 | GS/GX/4289/05 | A | ABL08603 |
| A/goose/Guangxi/4513/2005 | GS/GX/4513/05 | A | ABL08607 |
| A/goose/Guangxi/532/2006 | GS/GX/532/06 | A | ABL08711 |
| A/goose/Guangxi/5414/2005 | GS/GX/5414/05 | A | ABL08619 |
| A/goose/Guangxi/582/2006 | GS/GX/582/06 | A | ABL08631 |
| A/goose/Guangxi/914/2004 | GS/GX/914/04 | A | ABC68528 |
| A/goose/Guiyang/1461/2006 | GS/GY/1461/06 | A | ABL08799 |
| A/goose/Guiyang/1636/2006 | GS/GY/1636/06 | A | ABL08803 |
| A/goose/Guiyang/337/2006 | GS/GY/337/06 | A | ABL08579 |
| A/goose/Guiyang/3422/2005 | GS/GY/3422/05 | A | ABL08779 |
| A/goose/Guiyang/4180/2005 | GS/GY/4180/05 | A | ABL08821 |
| A/goose/Guiyang/538/2006 | GS/GY/538/06 | A | ABL08649 |
| A/goose/Guiyang/765/2006 | GS/GY/765/06 | A | ABL08811 |
| A/goose/Hong_Kong/1032.6/2000 | GS/HK/1032.6/00 | B | ACZ47211 |
| A/goose/Hong_Kong/485.3/2000 | GS/HK/485.3/00 | B | ACZ47209 |
| A/goose/Hong_Kong/668.1/2001 | GS/HK/668.1/01 | A | ACZ47319 |
| A/goose/Hong_Kong/739.2/2002 | GS/HK/739.2/02 | A | ACZ47317 |
| A/goose/Hubei/65/2005 | GS/HuB/65/05 | A | ADG59287 |
| A/goose/Jiangsu/220/2003 | GS/JS/220/03 | A | ABD23024 |
| A/goose/Jiangsu/k0403/2010 | GS/JS/k0403/10 | A | AFC98310 |
| A/goose/Jilin/hb/2003 | GS/JL/hb/03 | A | ABJ09531 |
| A/goose/Shantou/157/2002 | GS/st/157/02 | A | ACA47598 |
| A/goose/Shantou/18442/2005 | GS/st/18442/05 | A | ABL08653 |
| A/goose/Shantou/2216/2005 | GS/st/2216/05 | A | ABC68563 |
| A/goose/Shantou/239/2006 | GS/st/239/06 | A | ABL08787 |
| A/goose/Shantou/3265/2006 | GS/st/3265/06 | A | ABL08657 |
| A/goose/Shantou/3624/2006 | GS/st/3624/06 | A | ABL08815 |
| A/goose/Shantou/5456/2001 | GS/st/5456/01 | A | ACA47532 |
| A/goose/Shantou/753/2002 | GS/st/753/02 | A | ACA47631 |
| A/goose/Yunnan/3315/2005 | GS/YN/3315/05 | A | ABL08785 |
| A/goose/Yunnan/3720/2005 | GS/YN/3720/05 | A | ABL08747 |
| A/goose/Yunnan/4129/2005 | GS/YN/4129/05 | A | ABL08749 |
| A/goose/Yunnan/4371/2006 | GS/YN/4371/06 | A | ACH85426 |
| A/goose/Yunnan/5141/2006 | GS/YN/5141/06 | A | ACH85459 |
| A/goose/Yunnan/5299/2005 | GS/YN/5299/05 | A | ABL08767 |
| A/goose/Yunnan/5540/2006 | GS/YN/5540/06 | A | ACH85470 |
| A/goose/Yunnan/5599/2006 | GS/YN/5599/06 | A | ACH85481 |
| A/goose/Yunnan/5769/2006 | GS/YN/5769/06 | A | ACH85492 |
| A/goose/Yunnan/6368/2005 | GS/YN/6368/05 | A | ABL08595 |
| A/great_cormorant/Tibet/12/2006 | GC/Tibet/12/06 | A | ADG59347 |
| A/great_crested-grebe/Qinghai/1/2009 | GCG/QH/1/09 | A | ADG44993 |
| A/great_egret/Hong_Kong/807/2008 | GE/HK/807/08 | A | ACJ26335 |
| A/grey_heron/HK/861.1/2002 | GH/HK/861.1/02 | A | AAT73445 |
| A/grey_heron/Hong_Kong/1046/2008 | GH/HK/1046/08 | A | ACJ26346 |
| A/grey_heron/Hong_Kong/3088/2007 | GH/HK/3088/07 | A | ACJ26302 |
| A/grey_heron/Hong_Kong/728/2004 | GH/HK/728/04 | A | ABC68571 |
| A/grey_heron/Hong_Kong/837/2004 | GH/HK/837/04 | A | ABC68572 |
| A/house_crow/Hong_Kong/719/2007 | HC/HK/719/07 | A | ACJ26104 |
| A/lesser_kestrel/Heilongjiang/194/2007 | LK/HLJ/194/07 | A | ACZ54015 |
| A/little_egret/Hong_Kong/8550/2007 | LE/HK/8550/07 | A | ACJ26280 |
| A/little_egret/Hong_Kong/8863/2007 | LE/HK/8863/07 | A | ACJ26291 |
| A/long-tailed_shrike/Hong_Kong/2762/2007 | LTS/HK/2762/07 | A | ACJ26225 |
| A/magpie_robin/Hong_Kong/1897/2008 | MR/HK/1897/08 | A | ACJ26368 |
| A/magpie_robin/Hong_Kong/366/2006 | MR/HK/366/06 | A | ABL08567 |
| A/magpie_robin/Hong_Kong/75/2006 | MR/HK/75/06 | A | ABL08563 |
| A/mallard/Huadong/hn/2005 | MR/Hd/hn/05 | A | ABW21673 |
| A/mallard/Huadong/lk/2005 | MR/Hd/lk/05 | A | ABW21663 |
| A/migratory_duck/Jiangxi/2136/2005 | MDK/JX/2136/05 | A | ABC68567 |
| A/migratory_duck/Jiangxi/2295/2005 | MDK/JX/2295/05 | A | ABC68568 |
| A/ostrich/Suzhou/097/2003 | OT/sz/097/03 | A | AEB26721 |
| A/partridge/Shantou/1075/2002 | PE/st/1075/02 | A | ACA47642 |
| A/partridge/Shantou/478/2002 | PE/st/478/02 | A | ACA47609 |
| A/peregrine_falcon/HK/D0028/2004 | PF/HK/D0028/04 | A | AAT73453 |
| A/peregrine_falcon/Hong_Kong/1143/2007 | PF/HK/1143/07 | A | ACJ26137 |
| A/peregrine_falcon/Hong_Kong/2142/2008 | PF/HK/2142/08 | A | ACJ26379 |
| A/peregrine_falcon/Hong_Kong/5211/2006 | PF/HK/5211/06 | A | ACJ26071 |
| A/pheasant/Shantou/2239/2006 | PhT/st2239/06 | A | ABL08589 |
| A/pheasant/Shantou/3535/2003 | PhT/st/3535/03 | A | ACA47785 |
| A/pheasant/Shantou/40/2003 | PhT/st/40/03 | A | ACA47730 |
| A/pheasant/Shantou/4567/2002 | PhT/st/4567/02 | A | ACA47719 |
| A/pika/Qinghai/BI/2007 | PKQH/BI/07 | A | ACT31472 |
| A/pika/Qinghai/HMH/2007 | PK/QH/HMH/07 | A | ACT31486 |
| A/pika/Qinghai/QW/2007 | PK/QH/QW/07 | A | ACT31516 |
| A/pika/Qinghai/SHK/2007 | PK/QH/SHK/07 | A | ACT31505 |
| A/quail/Guangxi/575/2005 | QL/GX/575/05 | A | ABC68545 |
| A/quail/Shantou/3846/2002 | QL/st/3846/02 | A | ACA47675 |
| A/quail/Shantou/5164/2001 | QL/st/5164/01 | A | ACA47521 |
| A/quail/yunnan/092/2002 | QL/YN/092/02 | A | ABD23025 |
| A/raccoon_dog/Shandong/sd2/2005 | RD/SD/sd2/05 | A | ACB59329 |
| A/scaly-breasted_munia/Hong_Kong/2433/2007 | SBM/HK/2433/07 | A | ACJ26192 |
| A/scaly-breasted_munia/Hong_Kong/45/2007 | SBM/HK/45/07 | A | ACJ26082 |
| A/shrike/Tibet/13/2006 | SE/TB/13/06 | A | ADG59325 |
| A/silky_chicken/Shantou/475/2004 | SCK/st/475/04 | A | ACA47818 |
| A/swan/Shanghai/10/2009 | SN/10/09 | A | AEG20985 |
| A/swine/Fujian/1/2003 | SW/FJ/1/03 | A | AAV30832 |
| A/swine/Fujian/F1/2001 | SW/FJ/F1/01 | A | AAV30840 |
| A/swine/Henan/wy/2004 | SW/HeN/wy/04 | A | ABJ16476 |
| A/swine/Jiangsu/1/2008 | SW/JS1/08 | A | AGH25297 |
| A/swine/Jiangsu/2/2009 | SW/JS2/09 | A | AGH25299 |
| A/tiger/Shanghai/01/2005 | TG/SH/01/05 | A | ACB54712 |
| A/tree_sparrow/HK/864/2002 | TS/HK/864/02 | A | AAT73449 |
| A/tree_sparrow/Jiangsu/1/2008 | TS/JS/1/08 | A | ACR48931 |
| A/waterfowl/Hong_Kong/378.5/2001 | WFL/HK/378.5/01 | A | ACZ36651 |
| A/wild_duck/Guangdong/314/2004 | WDKGD/314/04 | A | AAX53549 |
| A/wild_duck/Hunan/021/2005 | WDKHN/021/05 | A | ABX83943 |
| A/wild_duck/Hunan/211/2005 | WDKHN/211/05 | A | ABX83945 |
| A/wild_duck/Liaoning/8/2006 | WDKLN/8/06 | A | ADG59301 |
