# Supplementary material for: Genetic Dynamic Analysis of the Influenza A H5N1 NS1 Gene in China
Source: PLoS One. 2014 Jul 8;9(7):e101384. doi: 10.1371/journal.pone.0101384 (PMC4086889; doi:10.1371/journal.pone.0101384)
Supplement: Table S7 — Distribution of PL motifs in 462 influenza NS1 protein sequences. PDZ-domain ligand sequences are listed in the PL column and the distribution of each PL sequence in avian and mammalian isolates is shown. (DOC) [file pone.0101384.s010.doc]

| **Table S7 Distribution of PL motifs in 462 influenza NS1 protein sequences.** PDZ-domain ligand sequences are listed in the PL column and the distribution of each PL sequence in avian and mammalian isolates is shown. | | | | | | | | |
| --- | --- | --- | --- | --- | --- | --- | --- | --- |
| PL | Avian | | | | Mammal | | | |
|  | duck | chicken | goose | wild birds | human | swine | raccoon dog | tiger |
| ESKV | 10 | 14 | 4 | 15 | 0 | 0 | 0 | 0 |
| ESEV | 101 | 89 | 41 | 56 | 30 | 5 | 1 | 1 |
| GSEV | 1 | 1 | 0 | 7 | 1 | 0 | 0 | 0 |
| ESEI | 0 | 2 | 1 | 2 | 0 | 0 | 0 | 0 |
| ES- - | 24 | 6 | 12 | 2 | 0 | 0 | 0 | 0 |
| - - - - | 2 | 6 | 1 | 0 | 0 | 0 | 0 | 0 |
| ESE- | 3 | 0 | 0 | 0 | 0 | 0 | 0 | 0 |
| EPEV | 4 | 7 | 0 | 0 | 11 | 0 | 0 | 0 |
| KSEV | 0 | 2 | 0 | 0 | 0 | 0 | 0 | 0 |
| Totals | 145 | 127 | 59 | 82 | 42 | 5 | 1 | 1 |
