# Supplementary material for: Genetic Dynamic Analysis of the Influenza A H5N1 NS1 Gene in China
Source: PLoS One. 2014 Jul 8;9(7):e101384. doi: 10.1371/journal.pone.0101384 (PMC4086889; doi:10.1371/journal.pone.0101384)
Supplement: Table S8 — A list of selected genes involved in PI3K signaling. (DOC) [file pone.0101384.s011.doc]

| **Table S8 A list of selected genes involved in PI3K signaling.** | | | |
| --- | --- | --- | --- |
| Symbol | Description | Gene Name | GeneBank |
| ADAR | Adenosine deaminase, RNA-specific | ADAR1, DRADA, DSH, DSRAD, G1P1, IFI-4, IFI4, K88DSRBP, P136 | XM_001111902.2 |
| AKT1 | V-akt murine thymoma viral oncogene homolog 1 | AKT, MGC99656, PKB, PKB-ALPHA, PRKBA, RAC, RAC-ALPHA | NM_001261625.1 |
| AKT3 | V-akt murine thymoma viral oncogene homolog 3 (protein kinase B, gamma) | DKFZp434N0250, PKB-GAMMA, PKBG, PRKBG, RAC-PK-gamma, RAC-gamma, STK-2 | NM_001266640.1 |
| BAD | BCL2-associated agonist of cell death | BBC2, BCL2L8 | NM_001265956.1 |
| BTK | Bruton agammaglobulinemia tyrosine kinase | AGMX1, AT, ATK, BPK, IMD1, MGC126261, MGC126262, PSCTK1, XLA | XM_002806330.1 |
| CASP9 | Caspase 9, apoptosis-related cysteine peptidase | APAF-3, APAF3, CASPASE-9c, ICE-LAP6, MCH6 | XM_001082859.2 |
| CCND1 | Cyclin D1 | BCL1, D11S287E, PRAD1, U21B31 | NM_001278446.1 |
| CHUK | Conserved helix-loop-helix ubiquitous kinase | IKBKA, IKK-alpha, IKK1, IKKA, NFKBIKA, TCF16 | NM_001265946.1 |
| CSNK2A1 | Casein kinase 2, alpha 1 polypeptide | CK2A1, CKII | XM_001101458.2 |
| CTNNB1 | Catenin (cadherin-associated protein), beta 1, 88kDa | CTNNB, DKFZp686D02253, FLJ25606, FLJ37923 | NM_001266127.2 |
| EIF2AK2 | Eukaryotic translation initiation factor 2-alpha kinase 2 | EIF2AK1, MGC126524, PKR, PRKR | NM_001083948.1 |
| EIF4E | Eukaryotic translation initiation factor 4E | CBP, EIF4E1, EIF4EL1, EIF4F, MGC111573 | NM_001266219.2 |
| EIF4EBP1 | Eukaryotic translation initiation factor 4E binding protein 1 | 4E-BP1, 4EBP1, BP-1, MGC4316, PHAS-I | NM_001257910.1 |
| EIF4G1 | Eukaryotic translation initiation factor 4 gamma, 1 | DKFZp686A1451, EIF-4G1, EIF4F, EIF4G, EIF4GI, P220 | XM_002802215.1 |
| FASLG | Fas ligand (TNF superfamily, member 6) | APT1LG1, CD178, CD95-L, CD95L, FASL, TNFSF6 | NM_001032838.1 |
| FKBP1A | FK506 binding protein 1A, 12kDa | FKBP-12, FKBP1, FKBP12, PKC12, PKCI2, PPIASE | XM_001086691.2 |
| FOXO3 | Forkhead box O3 | AF6q21, DKFZp781A0677, FKHRL1, FKHRL1P2, FOXO2, FOXO3A, MGC12739, MGC31925 | XM_001093593.2 |
| GRB10 | Growth factor receptor-bound protein 10 | GRB-IR, Grb-10, IRBP, KIAA0207, MEG1, RSS | NM_001257428.1 |
| GSK3B | Glycogen synthase kinase 3 beta | - | NM_001266636.2 |
| HRAS | V-Ha-ras Harvey rat sarcoma viral oncogene homolog | C-BAS, HAS, C-H-RAS, C-HA-RAS1, CTLO, H-RASIDX, HAMSV, HRAS1, K-RAS, N-RAS, RASH1 | NM_001266421.1 |
| KRAS | v-Ki-ras2 Kirsten rat sarcoma viral oncogene homolog |  | NM_001261512.2 |
| NRAS | neuroblastoma RAS viral (v-ras) oncogene homolog |  | XM_001111729.2 |
| IGF1 | Insulin-like growth factor 1 (somatomedin C) | IGF-I, IGF1A, IGFI | NM_001260726.1 |
| IGF1R | Insulin-like growth factor 1 receptor | CD221, IGFIR, IGFR, JTK13, MGC142170, MGC142172, MGC18216 | NM_001261352.1 |
| ILK | Integrin-linked kinase | DKFZp686F1765, ILK-2, P59 | NM_001195713.1 |
| ITGB1 | Integrin, beta 1 (fibronectin receptor, beta polypeptide, antigen CD29 includes MDF2, MSK12) | CD29, FNRB, GPIIA, MDF2, MSK12, VLA-BETA, VLAB | XM_002805609.1 |
| JUN | Jun proto-oncogene | AP-1, AP1, c-Jun | NM_001265850.2 |
| MAP2K1 | Mitogen-activated protein kinase kinase 1 | MAPKK1, MEK1, MKK1, PRKMK1 | NM_001257549.1 |
| MAPK1 | Mitogen-activated protein kinase 1 | ERK, ERK2, ERT1, MAPK2, P42MAPK, PRKM1, PRKM2, p38, p40, p41, p41mapk | XM_001089600.2 |
| MAPK14 | Mitogen-activated protein kinase 14 | CSBP, CSBP1, CSBP2, CSPB1, EXIP, Mxi2, PRKM14, PRKM15, RK, SAPK2A, p38, p38ALPHA | XM_001112524.2 |
| MAPK3 | Mitogen-activated protein kinase 3 | ERK1, HS44KDAP, HUMKER1A, MGC20180, P44ERK1, P44MAPK, PRKM3 | XM_001089600.2 |
| MAPK8 | Mitogen-activated protein kinase 8 | JNK, JNK1, JNK1A2, JNK21B1, 2, PRKM8, SAPK1 | XM_001108815.2 |
| MTOR | Mechanistic target of rapamycin (serine/threonine kinase) | FLJ44809, FRAP, FRAP1, FRAP2, RAFT1, RAPT1 | XM_002808289.1 |
| NFKB1 | Nuclear factor of kappa light polypeptide gene enhancer in B-cells 1 | DKFZp686C01211, EBP-1, KBF1, MGC54151, NF-kappa-B, NF-kappaB, NFKB-p105, NFKB-p50, NFkappaB, p105, p50 | NM_001266053.1 |
| NFKBIA | Nuclear factor of kappa light polypeptide gene enhancer in B-cells inhibitor, alpha | IKBA, MAD-3, NFKBI | NM_001257750.1 |
| PABPC1 | Poly(A) binding protein, cytoplasmic 1 | PAB1, PABP, PABP1, PABPC2, PABPL1 | NM_001257897.1 |
| PAK1 | P21 protein (Cdc42/Rac)-activated kinase 1 | MGC130000, MGC130001, PAKalpha | XM_001100062.2 |
| PDK1 | Pyruvate dehydrogenase kinase, isozyme 1 | - | XM_001093471.2 |
| PDK2 | Pyruvate dehydrogenase kinase, isozyme 2 | PDHK2 | XM_001093471.2 |
| PIK3CA | Phosphoinositide-3-kinase, catalytic, alpha polypeptide | MGC142161, MGC142163, PI3K, p110-alpha | NM_001260668.1 |
| PIK3CG | Phosphoinositide-3-kinase, catalytic, gamma polypeptide | PI3CG, PI3K, PI3Kgamma, PIK3 | NM_001266758.1 |
| PIK3R1 | Phosphoinositide-3-kinase, regulatory subunit 1 (alpha) | GRB1, p85, p85-ALPHA | NM_001261197.1 |
| PRKCB | Protein kinase C, beta | MGC41878, PKC-beta, PKCB, PRKCB1, PRKCB2 | NM_001257514.1 |
| PRKCZ | Protein kinase C, zeta | PKC-ZETA, PKC2 | XM_001089400.2 |
| PTEN | Phosphatase and tensin homolog | 10q23del, BZS, DEC, GLM2, MGC11227, MHAM, MMAC1, PTEN1, TEP1 | NM_001260965.1 |
| PTK2 | PTK2 protein tyrosine kinase 2 | FADK, FAK, FAK1, FRNK, pp125FAK | XM_001093060.2 |
| PTPN11 | Protein tyrosine phosphatase, non-receptor type 11 | BPTP3, CFC, MGC14433, NS1, PTP-1D, PTP2C, SH-PTP2, SH-PTP3, SHP2 | NM_001261109.1 |
| RAC1 | Ras-related C3 botulinum toxin substrate 1 (rho family, small GTP binding protein Rac1) | MGC111543, Rac-1, TC-25, p21-Rac1 | NM_001261377.1 |
| RAF1 | V-raf-1 murine leukemia viral oncogene homolog 1 | CRAF, NS5, Raf-1, c-Raf | NM_001266302.1 |
| RASA1 | RAS p21 protein activator (GTPase activating protein) 1 | CM-AVM, CMAVM, DKFZp434N071, GAP, PKWS, RASA, RASGAP, p120GAP, p120RASGAP | XM_001084074.2 |
| RBL2 | Retinoblastoma-like 2 (p130) | FLJ26459, P130, Rb2 | XM_002802476.1 |
| RHEB | Ras homolog enriched in brain | MGC111559, RHEB2 | XM_001105885.1 |
| RHOA | Ras homolog gene family, member A | ARH12, ARHA, RHO12, RHOH12 | XM_001096298.2 |
| RPS6KA1 | Ribosomal protein S6 kinase, 90kDa, polypeptide 1 | HU-1, MAPKAPK1A, RSK, RSK1 | XM_002808278.1 |
| RPS6KB1 | Ribosomal protein S6 kinase, 70kDa, polypeptide 1 | PS6K, S6K, S6K1, STK14A, p70(S6K)-alpha, p70-S6K, p70-alpha | XM_001109701.2 |
| SHC1 | SHC (Src homology 2 domain containing) transforming protein 1 | FLJ26504, SHC, SHCA | NM_001261487.1 |
| SOS1 | Son of sevenless homolog 1 (Drosophila) | GF1, GGF1, GINGF, HGF, NS4 | NM_001260585.1 |
| TLR4 | Toll-like receptor 4 | ARMD10, CD284, TOLL, hToll | NM_001037092.1 |
| TOLLIP | Toll interacting protein | FLJ33531, IL-1RAcPIP | NM_001261271.1 |
| TSC1 | Tuberous sclerosis 1 | KIAA0243, LAM, MGC86987, TSC | XM_001103846.2 |
| TSC2 | Tuberous sclerosis 2 | FLJ43106, LAM, TSC4 | XM_001083256.2 |
| WASL | Wiskott-Aldrich syndrome-like | DKFZp779G0847, MGC48327, N-WASP, NWASP | XM_001104246.2 |
| ATF4 | cyclic AMP-dependent transcription factor ATF-4 | ATF4, CREB-2, CREB2, TAXREB67, TXREB | NM_001266632.2 |
| ATF6B | cyclic AMP-dependent transcription factor ATF-6 beta-like | ATF6B, CREB-RP, CREBL1, G13 | XM_002803682.1 |
| BCL2 | bcl-2-like protein 1 | BCL2, Bcl-2, PPP1R50 | NM_001260717.1 |
| Bim | bcl-2-like protein 11 | BCL2L1, BCL-XL/S, BCL2L, BCLX, BCLXL, BCLXS, Bcl-X, PPP1R52, bcl-xL, bcl-xS | XM_001086237.2 |
| CHN1 | n-chimaerin isoform 5 | ATF2, CRE-BP1, CREB2, HB16, TREB7 | XM_001093000.2 |
| CREB1 | cyclic AMP-responsive element-binding protein 1 | CREB1, CREB | NM_001261120.1 |
| CREB3 | cyclic AMP-responsive element-binding protein 3 | CREB3, LUMAN, LZIP | NM_001265647.1 |
| CREB3L4 | cyclic AMP-responsive element-binding protein 3-like protein 4" | CREB3L4, AIBZIP, ATCE1, CREB3, CREB4, JAL, hJAL | NM_001265999.1 |
| G6PC2 | glucose-6-phosphatase 2-like | G6PC2, IGRP | XM_001114903.1 |
| G6PC3 | glucose-6-phosphatase 3 | G6PC3, SCN4, UGRP | NM_001257733.1 |
| GYS1 | glycogen [starch] synthase, muscle | GYS1, GSY, GYS | NM_001032886.1 |
| IRF-3 | interferon regulatory factor 3 |  | NM_001135797.1 |
| MCL1 | myeloid cell leukemia sequence 1 (BCL2-related | MCL1, BCL2L3, EAT, MCL1-ES, MCL1L, MCL1S, Mcl-1, TM, bcl2-L-3, mcl1/EAT | NM_001266501.2 |
| MYB | transcriptional activator Myb-like | MYB, Cmyb, c-myb, c-myb_CDS, efg | XM_001101267.2 |
| PCK1 | phosphoenolpyruvate carboxykinase, cytosolic [GTP] |  | XM_001086710.2 |
| PCK2 | phosphoenolpyruvate carboxykinase [GTP],mitochondrial |  | NM_001265937.2 |
| PIK3CB | phosphatidylinositol-4,5-bisphosphate 3-kinase catalytic subunit beta isoform isoform 2 | PIK3CB, P110BETA, PI3K, PI3KBETA, PIK3C1 | XM_001113960.2 |
| S6 | 40S ribosomal protein S6-like isoform 6 | RPS6, S6 | XM_001109421.2 |
| SGK1 | phosphoenolpyruvate carboxykinase 2 (mitochondrial) | SGK1, SGK | XM_001102277.2 |
| SGK2 | serine/threonine-protein kinase Sgk2-like | SGK2, H-SGK2, dJ138B7.2 | XM_001083986.2 |
| SGK3 | serine/threonine-protein kinase Sgk3-like | SGK3, CISK, SGK2, SGKL | XM_002805366.1 |
| YWHAB | 14-3-3 protein beta/alpha | YWHAB, GW128, HS1, KCIP-1, YWHAA | NM_001258344.1 |
| YWHAQ | 14-3-3 protein theta isoform 1 | YWHAQ, 14-3-3, 1C5, HS1 | XM_001097635.2 |
