# Supplementary material for: Genetic Dynamic Analysis of the Influenza A H5N1 NS1 Gene in China
Source: PLoS One. 2014 Jul 8;9(7):e101384. doi: 10.1371/journal.pone.0101384 (PMC4086889; doi:10.1371/journal.pone.0101384)

Figure S3 Phylogenetic analysis of the NS1 gene based on 462 nucleotide sequences of HPAI H5N1 isolates. A small branch of the A allele contained the sequence AIASS at position 80-84 is highlighted in green.

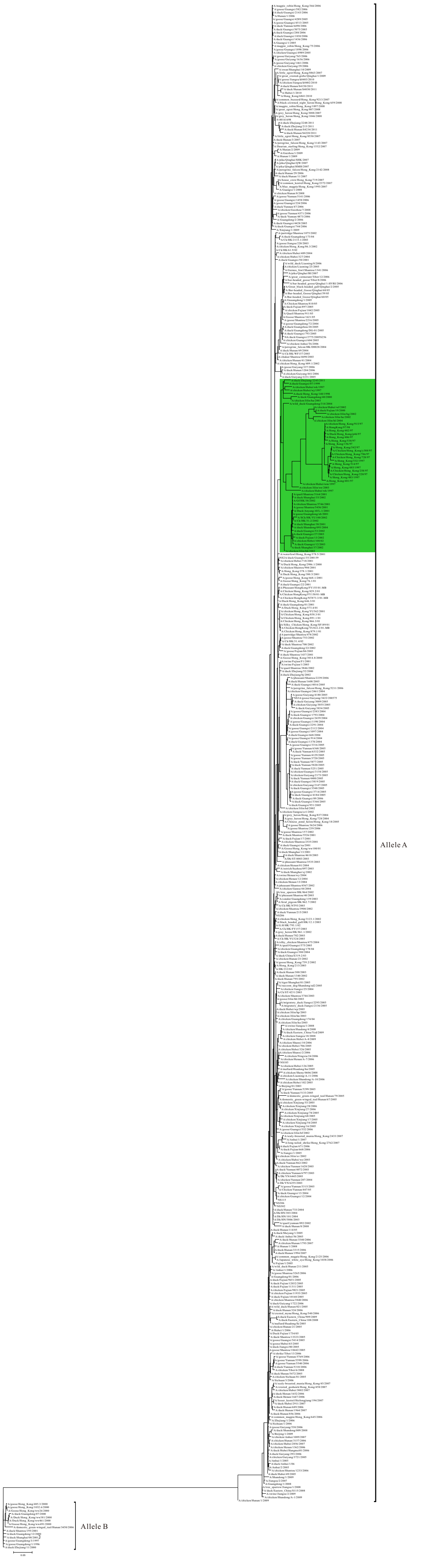

Supplement: Figure S3 — Phylogenetic analysis of the NS1 gene based on 462 nucleotide sequences of HPAI H5N1 isolates. A small branch of the A allele contained the sequence AIASS at position 80–84 is highlighted in green. (PDF) [file pone.0101384.s003.pdf]
